# Midkine noncanonically suppresses AMPK activation through disrupting the LKB1-STRAD-Mo25 complex

**DOI:** 10.1038/s41419-022-04801-0

**Published:** 2022-04-29

**Authors:** Tian Xia, Di Chen, Xiaolong Liu, Huan Qi, Wen Wang, Huan Chen, Ting Ling, Wuxiyar Otkur, Chen-Song Zhang, Jongchan Kim, Sheng-Cai Lin, Hai-long Piao

**Affiliations:** 1 CAS Key Laboratory of Separation Science for Analytical Chemistry, Dalian Institute of Chemical Physics, Chinese Academy of Sciences, Dalian, 116023 China; 2 State Key Laboratory of Cellular Stress Biology, School of Life Sciences, Xiamen University, Fujian, 361102 China; 3 Department of Life Sciences, Sogang University, Seoul, 04107 Republic of Korea

**Keywords:** Cancer, Oncogenes

## Abstract

Midkine (MDK), a secreted growth factor, regulates signal transduction and cancer progression by interacting with receptors, and it can be internalized into the cytoplasm by endocytosis. However, its intracellular function and signaling regulation remain unclear. Here, we show that intracellular MDK interacts with LKB1 and STRAD to disrupt the LKB1-STRAD-Mo25 complex. Consequently, MDK decreases the activity of LKB1 to dampen both the basal and stress-induced activation of AMPK by glucose starvation or treatment of 2-DG. We also found that MDK accelerates cancer cell proliferation by inhibiting the activation of the LKB1-AMPK axis. In human cancers, compared to other well-known growth factors, MDK expression is most significantly upregulated in cancers, especially in liver, kidney and breast cancers, correlating with clinical outcomes and inversely correlating with phosphorylated AMPK levels. Our study elucidates an inhibitory mechanism for AMPK activation, which is mediated by the intracellular MDK through disrupting the LKB1-STRAD-Mo25 complex.

## Introduction

AMP-activated protein kinase (AMPK), consisting of catalytic subunit α and regulatory subunit β and γ [1], is the core cellular energy sensor and regulator [2]. Phosphorylation at Thr172 of α subunit leads to the activation of AMPK [3, 4], which directly phosphorylates a series of substrates to postpone energy-consuming processes, such as cell proliferation and fatty acid synthesis, and to promote energy-producing procedures, including catabolism and autophagy [5, 6]. Although other phosphorylation sites of AMPKα have been reported [7, 8], the phosphorylation of AMPKα Thr172 is primary activation of AMPK [9]. AMPK is closely related to diverse diseases [10], including dual and controversial roles in cancer [11–13]. Although defined as a tumor suppressor by many studies [14–17], AMPK promotes cancer progression under certain conditions by rescuing cancer cells from nutrient deficiency [18–21].

LKB1, CAMKKβ, and TAK1 are upstream kinases of AMPK that all phosphorylate AMPKα at the Thr172 site [5]. Among them, the serine/threonine kinase LKB1 mediates the best-characterized classical AMPK activation route [22], especially in cancer cells. LKB1 is well-known to form a heterotrimer with pseudokinase STRAD and scaffolding protein Mo25 and then undergoes self-phosphorylation at multiple amino acids to self-induce its kinase activity [23–25]. Some studies have demonstrated that disruption of the LKB1-STRAD-Mo25 complex decreases AMPKα Thr172 phosphorylation levels and attenuates AMPK activity [26].

Midkine (MDK, encoded by the *MDK* gene) is a pleiotrophin family growth factor that plays vital roles in different physiological processes, such as embryo and nerve development, blood pressure control, inflammation, and immune response [27–30]. MDK is highly expressed in different types of cancer [31–33] and promotes tumor progression by positively regulating cell proliferation, anti-apoptosis, metastasis, angiogenesis and immune-resistance [34–42]. As a secreted protein, MDK binds transmembrane receptors [43] to activate intracellular signaling [43–47]. It has also been reported that extracellular MDK can be transported into the cytosol by endocytosis and then enter the nuclei where it undergoes proteasomal degradation, but the intracellular functions of MDK are still unclear [48–50].

Here, we report that intracellular MDK suppresses AMPK activation by interacting with LKB1 and STRAD to depolymerize the LKB1-STRAD-Mo25 complex and reduce LKB1 activity, consequently decreasing the phosphorylation of AMPKα. Decreasing the cellular MDK expression level or maintaining the extracellular localization of MDK elevates AMPKα phosphorylation. Our results clarify that MDK promotes cancer cell proliferation by suppressing the LKB1-AMPK axis, and MDK expression correlates with clinical outcomes and inversely correlates with LKB1/AMPK signaling pathway activation.

## Results

### Midkine suppresses AMPK activation in an intracellular localization-dependent manner

**Fig. 1:**
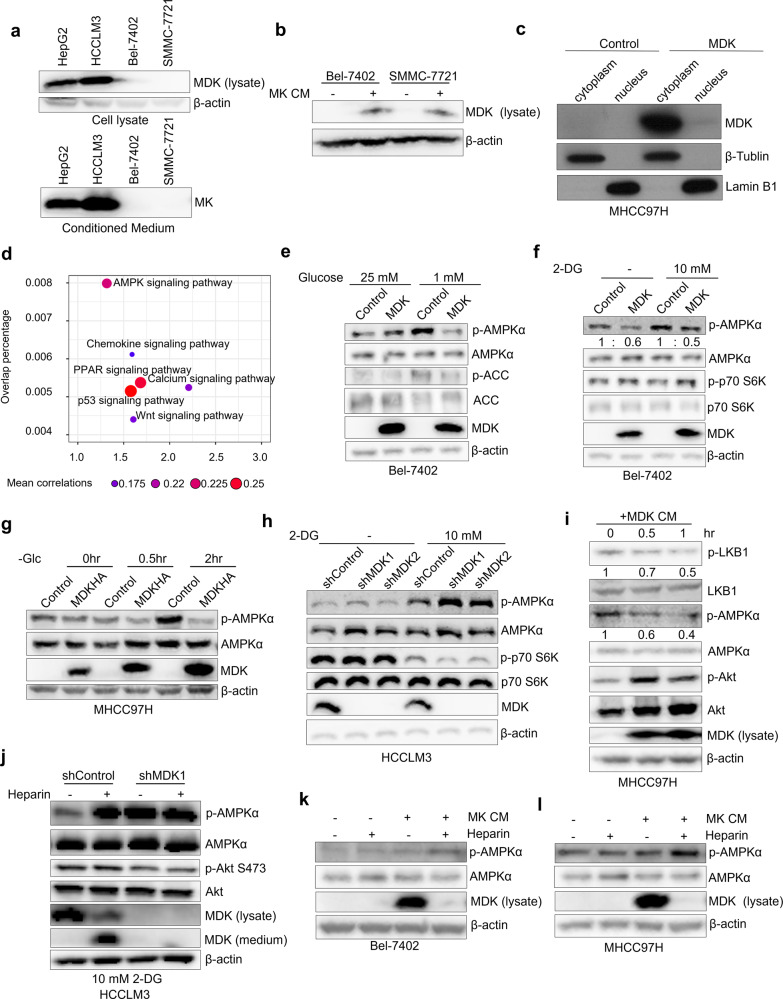
Midkine suppresses AMPK activation in an intracellular localization-dependent manner. **a** MDK expression in the cell lysate and conditioned medium (CM) of HepG2, HCCLM3, Bel-7402, and SMMC-7721 cells was tested by immunoblotting. **b** CM from MDK-overexpressing MHCC97H cells was harvested and used to culture Bel-7402 and SMMC-7721 cells for 2 h, intracellular MDK was tested by western blotting. **c** Cytoplasmic and nuclear fractions of the MDK overexpressing MHCC97H cells and the control cells was prepared and the distribution of MDK was detected by immunoblotting. **d** Signaling pathway enrichment assay with MDK-correlated genes based on the TCGA database. We first defined MDK correlated genes by calculating the Pearson correlation coefficients between MDK and all other genes according to their mRNA expression levels (data from the TCGA RNA-seq datasets). Then the overlap significance between MDK correlated genes and pathway genes (data from KEGG) was examined. Then the GSEA-based pathway enrichment was performed. **e**–**f** Analysis of p-AMPKα Thr172 level affected by MDK. MDK overexpressing Bel-7402 cells and control cells were treated at different glucose concentration (25 mM and 1 mM) for 2 h (**e**), or with 10 mM 2DG for 4 h (**f**), then the cells was lysed and the indicated proteins were tested with immunoblotting. **g** MDK overexpressing cells and control cells were subjected to glucose starvation for the indicated time, then the cells were harvested for western blotting. **h** Knock-down of MDK in HCCLM3 cells with two independent shRNAs resulted in elevated p-AMPKα Thr172 level after 4 h treatment of 10 mM 2DG. **i** Western blotting of the MHCC97H cells at different time points of CM treatment. CM is from MDK-overexpressing MHCC97H cells. **j** MDK knocked-down HCCLM3 cells and the control cells were treated with or without heparin treatment (30 μg/ml) for 4 h under 10 mM 2DG culture conditions, following by immunoblotting to test the indicated protein level. **k**–**l** The Bel-7402 (**k**) and MHCC97H (**l**) cells were subjected to a combination treatment with or without MDK- CM and heparin (30 μg/ml) for 2 h, then western blotting was performed to test the p-AMPKα Thr172 level.

Although usually MDK is considered as a secreted growth factor, but there are still arguments in its localization [48, 51]. To confirm this localization, we examined the transport and relocalization of MDK. Consistent with previous studies, MDK was secreted into the cell medium of HepG2 and HCCLM3 cells expressing high levels of MDK (Fig. 1a), and this secreted MDK was internalized into Bel-7402, SMMC-7721 and MHCC97H cells not expressing MDK (Fig. 1b, Supplementary Fig. S1a, b). However, intracellular MDK was mostly localized in the cytoplasm, not in the nucleus (Fig. 1c, Supplementary Fig. S1c, d). This phenomenon indicated that MDK may possess an unexplored function in the cytoplasm.

The MDK-regulated cell signaling pathway enrichment analysis showed that the AMPK signaling pathway was the most highly correlated with MDK among the pathways (Fig. 1d). To further examine the relationship between MDK and the AMPK pathway, we tested the level of phosphorylated AMPKα at Thr172 (only against this AMPK phosphorylation site in this study) in MDK-knockdown and MDK-overexpressing cells. In both Bel-7402 and MHCC97H cells, where MDK is low-expressed (Supplementary Fig. S5a), the overexpression of MDK inhibited the level of phosphorylated AMPKα during glucose starvation, 2-DG stimulation, or FBS deprivation (Fig. 1e-g, Supplementary Fig. S1e). In contrast, knocking down MDK expression by shRNA led to elevated levels of AMPKα phosphorylation (Fig. 1h, Supplementary Fig. S1f, g), and restoring MDK expression decreased AMPKα phosphorylation (Supplementary Fig. S1f, g). Taken together, these results suggest that MDK suppresses AMPK activation in human cancer cells.

MDK is well known to be secreted after the cleavage of signal peptide, and we have observed the internalization of MDK (Fig. 1b). Therefore, to understand whether internalized cellular MDK is critical for AMPK repression, we investigated the effect of extracellular MDK on AMPK activation. We collected CM containing secreted MDK from MDK-overexpressing MHCC97H cells and then used it to culture MDK-deficient MHCC97H parental cells. Upon the application of CM from MDK-overexpressing cells, the intracellular MDK level increased in a time-dependent manner, and this treatment, which triggered AKT phosphorylation as previously reported [52], decreased LKB1 and AMPKα phosphorylation (Fig. 1i). In addition, Bel-7402 cells cultured with control CM exhibited increased AMPK activation after 2-DG treatment, however, the cells cultured with CM from MDK-overexpressing cells showed decreased AMPK activation, even after 2-DG treatment (Supplementary Fig. S1h).

Next, to further clarify whether the intracellular relocalization of MDK is indispensable for AMPK suppression, we interfered the transportation of MDK into the cytoplasm. MDK is a heparin-binding protein [43, 53]. By adding heparin to the medium of the HCCLM3 cells, the intracellular MDK level decreased dramatically, and MDK significantly accumulated in the cell culture medium (Fig. 1j). Heparin reduced intracellular MDK and obviously elevated AMPKα phosphorylation in the HCCLM3 cells (Fig. 1j). In contrast, knocking down MDK did not alter AMPKα phosphorylation levels in cells during heparin application (Fig. 1j), excluding the possibility that AMPK is activated by heparin. Similarly, heparin decreased the intracellular MDK level in the MDK-overexpressing cells and promoted AMPKα phosphorylation in the Bel-7402 control cells (Supplementary Fig. S1i). Additionally, heparin caused decreased intracellular MDK levels and elevated AMPKα phosphorylation in MDK-CM-treated Bel-7402 and MHCC97H cells (Fig. 1k, l). These results indicate that intracellular MDK suppresses AMPK phosphorylation in a cytosol-dependent manner and corroborates findings indicating an intracellular function for MDK.

### Midkine associates with AMPK subunits and its upstream regulating factors

**Fig. 2:**
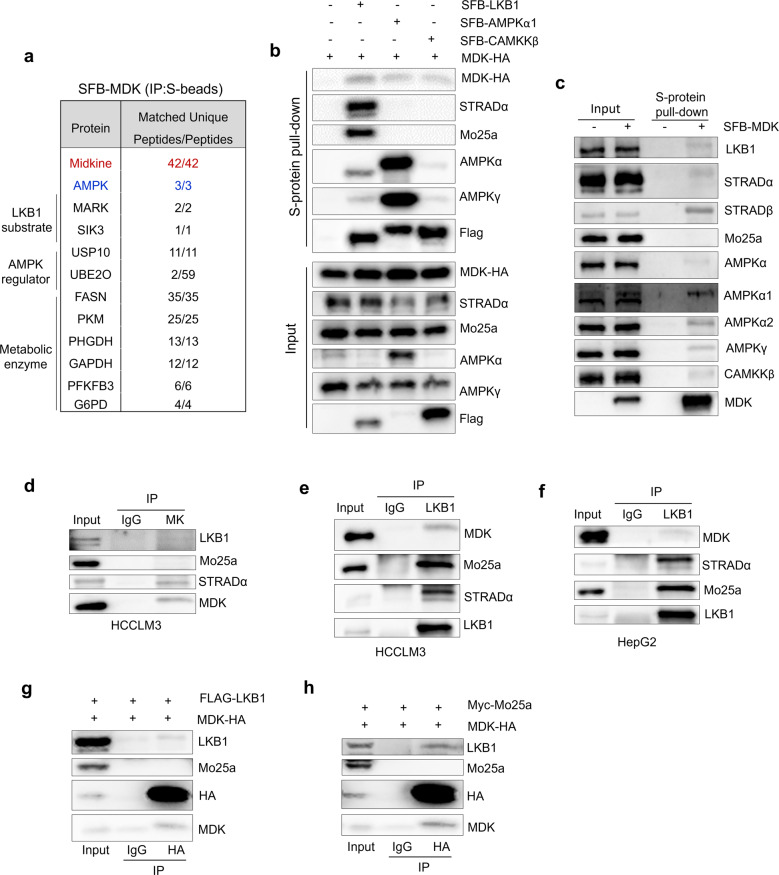
Midkine associates with AMPK subunits and its upstream regulating factors. **a** AMPK kinase subunit and LKB1 substrates were identified as MDK-associated proteins in HEK293A cells by tandem affinity purification–mass spectrometry. Bait MDK protein is marked in red. AMPKα is marked in blue. The right column of numbers represents unique peptide number/total peptide number. **b**, **c** MDK associates with LKB1, CAMKKβ, and AMPK subunits. The indicated constructs were expressed in the HEK293T cells for 24 h, and cell lysates were subjected to pull-down assays with S protein beads. **d** MDK was immunoprecipitated from HCCLM3 cells and subjected to Western blot analysis with antibodies against LKB1, Mo25a, STRADα, and MDK. **e**–**f** LKB1 immunoprecipitated from HCCLM3 (**e**) and HepG2 (**f**) cells and subjected to Western blot analysis with antibodies against MDK, Mo25a, STRADα, and LKB1. **g**–**h** HEK293T cells were cotransfected with MDK-HA and Flag-tagged LKB1 (**g**) or Myc-tagged Mo25a (**h**) and coimmunoprecipitated with HA primary antibody and subjected to Western blot analysis with antibodies against LKB1, HA, Mo25a and MDK.

Since MDK regulates the phosphorylation of LKB1 and AMPK, we tried to reveal the underlying mechanisms by which MDK functions. First, we isolated MDK-associated protein complexes in HEK293T cells through tandem affinity purification followed by mass spectrometry (MS) analysis. We found that some MDK-associated proteins belonged to LKB1 substrates, AMPK regulators, or metabolic regulation factors (Fig. 2a, Supplementary Fig S2a). Interestingly, MDK associated with the LKB1 substrates MARK and SIK3 of AMPK family proteins, and the well-studied AMPK ubiquitination regulators USP10 and UBE2O were also added to the prey list (Fig. 2a).

The specific interaction between MDK and the AMPK α subunit, as well as the well-studied AMPK upstream kinases LKB1 and CAMKKβ, was confirmed by pull-down assays (Fig. 2b, Supplementary Fig. S2b, c). Additionally, MDK formed complex with endogenous AMPK signaling components such as LKB1, STRADα/β, AMPKα1/2, AMPKγ, and CAMKKβ in MDK-transfected HEK293T cells (Fig. 2c). Furthermore, coimmunoprecipitation (co-IP) assays showed that LKB1 can be detected in endogenous MDK immunoprecipitates from HCCLM3 cells (Fig. 2d) and that endogenous MDK is pulled down with endogenous LKB1 immunoprecipitates from HCCLM3 and HepG2 cells (Fig. 2e, f). Interestingly, we noticed that STRADα and Mo25a, which bind LKB1 and facilitate LKB1 activation, showed different interaction ability with MDK (Fig. 2d-h, Supplementary Fig. S2b, c). STRADα was associated with both endogenous and exogenous MDK; however, Mo25a was not detected in either the endogenous co-IP or exogenous pull-down assays (Fig. 2c, d, g, h, Supplementary Fig. S2b, d). These results suggested that MDK may inhibit the protein machinery of LKB1-Mo25-STRAD.

Next, we wondered whether MDK interacts with LKB1 and AMPKα directly or indirectly via the kinase-substrate reaction. To test this hypothesis, we stably expressed MDK in LKB1-deficient A549 cells and found that AMPKα was detected only in the MDK coprecipitates from LKB1-reconstituted A549 cells (Supplementary Fig. S2d). This result indicated that the interaction between MDK and AMPK may be dependent on LKB1. LKB1 contains a kinase domain in the middle of its amino acid sequence (Fig. S2e). Although AMPKα was associated with different forms of LKB1 (the full-length protein, the N-terminus with the kinase domain only, and the C-terminus only), MDK interacted only with the NK domain (amino acids 1-309), which was similar to STRAD and Mo25 (Fig. 2e, f). In summary, we discovered that both MDK and AMPKα are physically associated with LKB1 through its N-terminal kinase domains in cells.

### Midkine suppresses AMPKα activation through LKB1

**Fig. 3:**
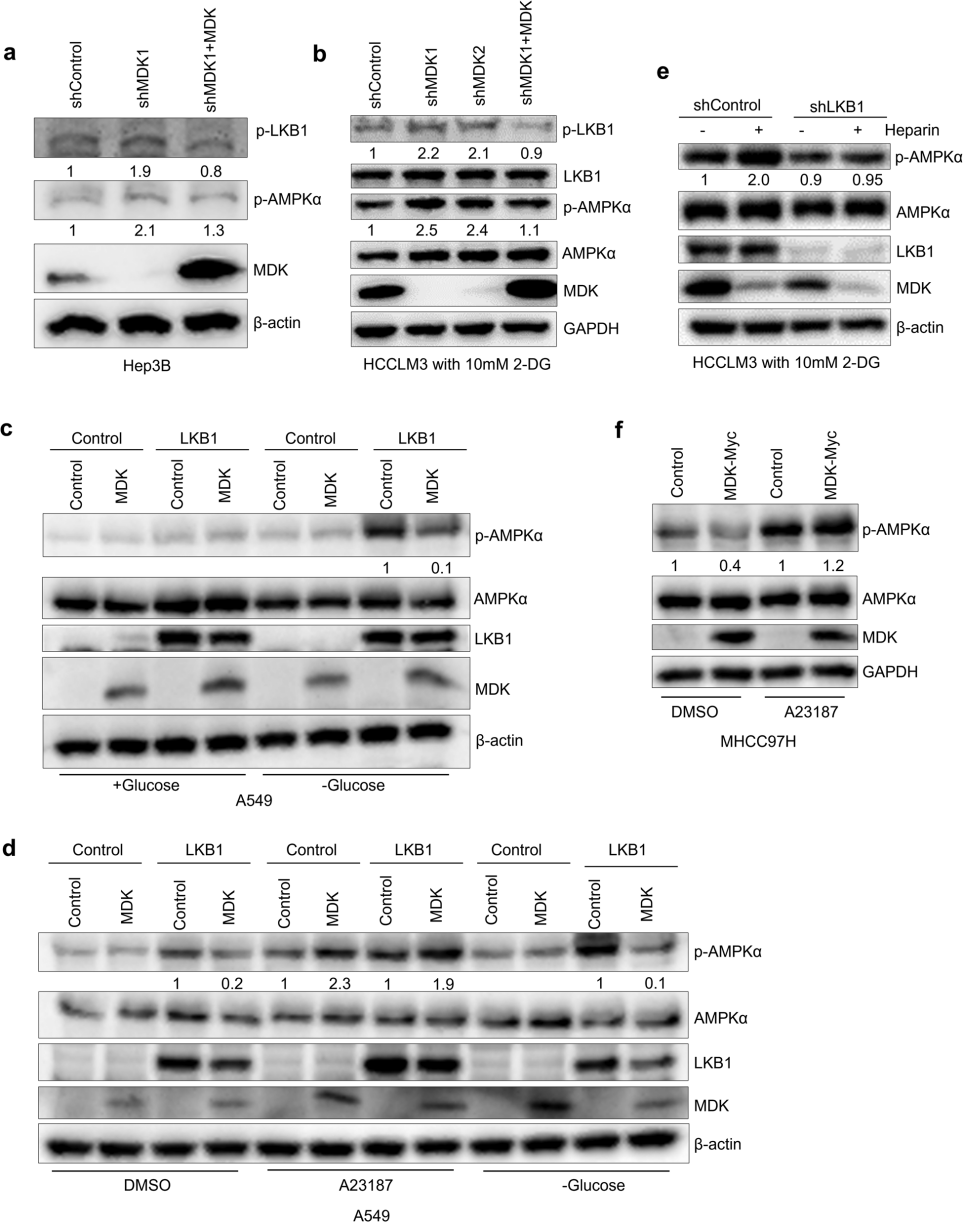
Midkine suppresses AMPKα activation through LKB1. **a**–**b** Western blot analysis of the Hep3B cells transduced with MDK shRNA1 and restored MDK in the MDK-knockdown cells (**a**) and the HCCLM3 cells transduced with two independent MDK shRNAs and restored MDK in the MDK-knockdown cells after 4 h of 10 mM 2-DG treatment (**b**). **c**–**d** Western blot analysis was performed with antibodies against p-LKB1, LKB1, p-AMPKα, AMPKα, MDK, and β-actin. Western blot analysis of the A549 cells transduced with LKB1 alone or in combination with MDK and the control cells with glucose starvation for 2 h (**c**) and the A549 cells transduced with LKB1 alone or in combination with MDK and the control cells with DMSO or A23187 (10 μg/ml) treatment or glucose starvation for 2 h (**d**). Western blot analysis was performed with antibodies against p-AMPKα, AMPKα, LKB1, MDK, and β-actin. **e** Western blot analysis of p-AMPKα, AMPKα, LKB1, MDK, and β-actin in the HCCLM3 cells transduced with LKB1 shRNA and the control cells with or without heparin (30 μg/ml) treatment and 10 mM 2-DG treatment for 4 h. **f** Western blot analysis of p-AMPKα, AMPKα, MDK, and β-actin from the MHCC97H cells transduced with MDK-Myc and the control cells treated with DMSO or A23187 (10 μg/ml).

LKB1 and calcium/calmodulin-dependent protein kinase kinase β (CAMKKβ) are well-known AMPK upstream kinases, and both kinases can phosphorylate the AMPK α subunit at the Thr172 site [22, 54]. Although MDK associates with LKB1 and CAMKKβ (Fig. 2b, c), whether MDK regulates AMPKα activities through LKB1 or CAMKKβ remains unclear. Interestingly, the activity of LKB1 and AMPKα was increased in MDK-knockdown Hep3B and HCCLM3 cells (Fig. 3a, b). In contrast, restoring MDK expression in these knockdown cells recovered LKB1 activity and AMPKα phosphorylation (Fig. 3a, b), suggesting that MDK contributed to the LKB1-modulated AMPKα suppression. Furthermore, in LKB1-deficient A549 cells, MDK overexpression did not alter AMPKα phosphorylation levels until LKB1 expression was restored (Fig. 3c, d), indicating that MDK suppressed AMPKα activation through LKB1. In HCCLM3 cells, AMPKα phosphorylation was elevated when the intracellular MDK levels were decreased by heparin treatment, but the effect of heparin was attenuated in LKB1-knockdown cells (Fig. 3e). These results indicated that LKB1 is involved in the MDK-induced regulation of AMPK activity.

To understand whether MDK mediates AMPK signaling through CAMKKβ, we used CAMKKβ activator A23187 to treat MDK-restoring MHCC97H cells. The overexpression of MDK suppressed AMPK activation upon DMSO treatment (control group); however, AMPKα phosphorylation was elevated regardless of the MDK expression after A23187 treatment (Fig. 3f). In agreement with this, A23187 stimulated AMPKα activation regardless of the level of MDK or LKB1 expression, while MDK suppressed AMPKα phosphorylation in the presence of LKB1 during glucose starvation (Fig. 3c, d). Considering these results, we speculated that MDK mediates AMPK activity through LKB1.

### Midkine disrupts LKB1-STRAD-Mo25 complex

Previously, LKB1 activation is dependent on the association of STRAD and Mo25 [24]. Our results demonstrated that MDK-mediated repression of AMPK activation relied on LKB1 (Fig. 3c–e), and the level of MDK expression was correlated with LKB1 phosphorylation (Fig. 3a, b). In addition, we found that MDK physically interacts with LKB1 and STRAD (Fig. 1b–h). Considering that the formation of the LKB1-STRAD-Mo25a complex is necessary for LKB1 activity, we surmised that MDK affects the stability of the LKB1-STRAD-Mo25 heterotrimer.

**Fig. 4:**
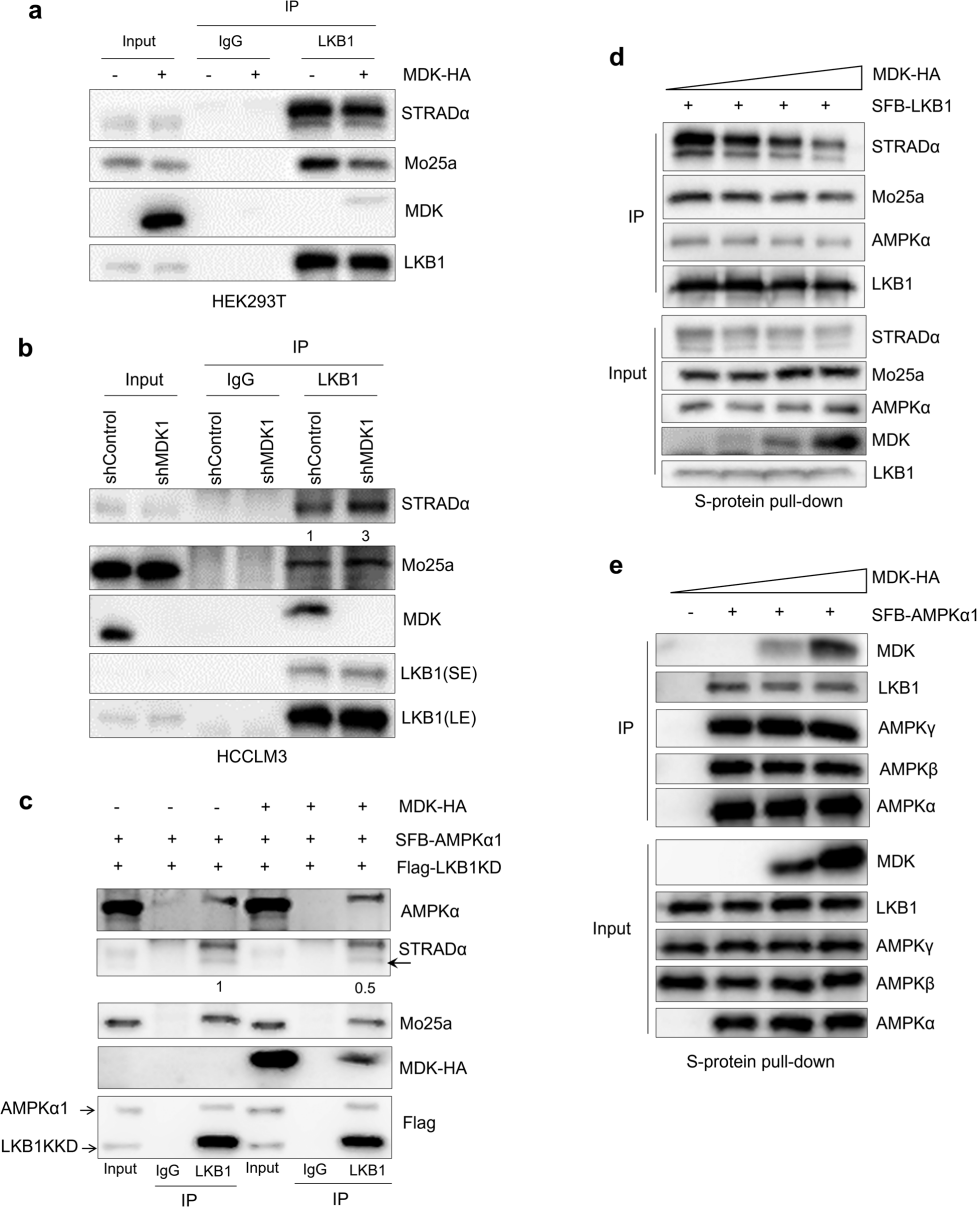
Midkine depolymerized LKB1-STRAD-Mo25 complex. **a**–**b** LKB1 was immunoprecipitated from the HEK293T cells transduced with MDK-HA (**a**) and the HCCLM3 cells transduced with MDK shRNA1 (**b**), and then, Western blot analysis was performed with antibodies against STRADα, Mo25a, MDK and LKB1. **c** LKB1 was immunoprecipitated from the HEK293T cells transduced with MDK-HA and the control cells followed by Western blotting with antibodies against AMPKα, STRADα, Mo25a, HA, and FLAG. The indicated constructs were expressed in the HEK293T cells for 48 h, and the cell lysates were subjected to coimmunoprecipitation with primary LKB1 and IgG antibodies. **d**–**e** HEK293T cells were cotransfected with different doses of MDK-HA and SFB-tagged LKB1 (**d**) or SFB-tagged AMPKα1 (**e**), pulled down with S protein beads, and subjected to Western blot analysis with antibodies against STRADα, Mo25a, LKB1, MDK and AMPK subunits.

To investigate this hypothesis, we performed coimmunoprecipitation assays with LKB1 from MDK-transduced cells. The overexpression of MDK significantly inhibited the formation of the LKB1-STRAD-Mo25a complex in endogenous LKB1 immunoprecipitates from HEK293T cells (Fig. 4a). In contrast, knocking down MDK increased the level of STRAD and Mo25 in the endogenous LKB1 immunoprecipitates from HCCLM3 cells (Fig. 4b). Furthermore, to prevent the phosphorylation of LKB1 from affecting this interaction, we expressed FLAG-tagged wild-type (WT) and kinase deleted (KD, K78L) LKB1 in HEK293T cells, and both of them bound strongly to STRAD and Mo25a (Supplementary Fig. S3a). Next, we simultaneously expressed FLAG-tagged LKB1-KD and SFB-tagged AMPKα1 in HEK293T cells. The coimmunoprecipitation assays showed that MDK overexpression decreased the binding of STRAD and Mo25a to LKB1; however, it did not affect the LKB1-AMPK interaction (Fig. 4c, Supplementary Fig. S3b). Moreover, gradually increasing MDK in the HEK293T cells was accompanied by gradually decreased LKB1-STRAD-Mo25a association, although the LKB1-AMPKα interaction was not affected (Fig. 4d). In contrast, gradually increasing MDK expression affected neither the association of AMPKα with the regulatory β and γ subunits nor the LKB1-AMPKα interaction (Fig. 4e, Supplementary Fig. S3c). To further evaluate the impact of secretion on MDK function, we deleted the three amino acids from 20 to 22 in MDK signal peptide, and named MDK-Del. MDK-Del showed a strong defect in the cleavage of signal peptide and could not detected in the medium (Supplementary Fig S3d). Similar to the wild type MDK, MDK-Del interacts with LKB1 in cells and attenuated the interaction of LKB1 to STRAD and Mo25 (Supplementary Fig. S3e, f). Taken together with the association of MDK with LKB1 and STRAD, these results suggest a mechanism by which MDK binds to LKB1 and STRAD and inhibits the formation of the LKB1-STRAD-Mo25a complex, leading to a decrease in LKB1 activity and AMPKα phosphorylation.

### Midkine expression is upregulated in cancers

**Fig. 5:**
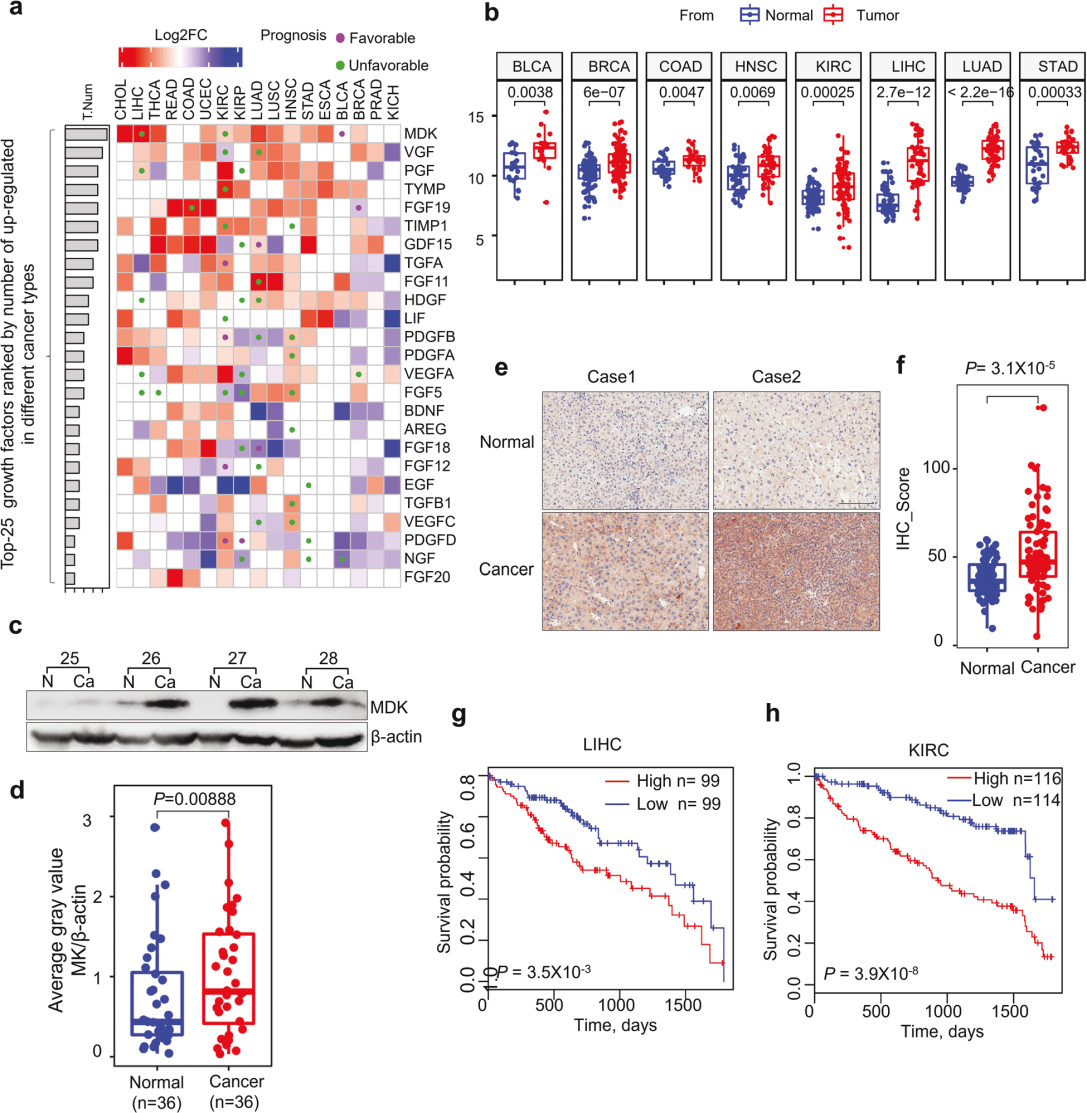
Midkine expression is upregulated in cancer. **a** Pan-cancer evaluation of the expression and prognostic impact of growth factors. The color of each rectangle represents the log2 transformed fold change (Log2FC) of the mRNA expression for the corresponding growth factor between tumor and normal tissues, and the white rectangles indicate either a Log2FC value equal 0 or differences between tumor and normal tissues that are not significant (linear model approach of limma, *P* > 0.01). The purple and green circles represent high gene expression correlated with good and poor prognosis, respectively (log rank test, *P* < 0.01). The left bars represent the numbers of cancer types in which a growth factor is upregulated in the tumor tissues compared with the normal tissues (*P* < 0.01, log2FC > 1). The growth factors are ranked by the numbers, and only the top 25 factors are shown. **b** Boxplots of the differences in MDK expression in paired normal and tumor tissues of eight types of cancers. The centers of the boxes represent the median values. The bottom and top boundaries of the boxes represent the 25th and 75th percentiles, respectively. The whiskers indicate 1.5-fold of the interquartile range. The dots represent points falling outside this range. The paired P-values were calculated based on Wilcox tests. **c**–**d** The expression of MDK in 36 pairs of matched adjacent nontumor (NT) and cancer (Ca) tissues as detected by Western blotting (**c**), and the distribution of MDK expression in both the NT and Ca samples as represented by boxplots with the expression value normalized by ImageJ software (**d**). **e**–**f** Immunohistochemical staining of MDK in representative adjacent nontumor and HCC specimens (**e**) and boxplots of the distributions of MDK expression status in 75 paired paraffin-embedded tissues (**f**). Scale bar, 200 μm. **g**–**h** Kaplan–Meier survival curves of LIHC (**g**) and KIRC (**h**) patients with data stratified by the expression levels obtained from the TCGA database.

To better clarify the biological function of MDK-mediated LKB1-AMPK axis suppression, we performed a disease enrichment analysis of MDK (Supplementary Fig. S4a). The result demonstrated that MDK was most frequently related to cancer (Supplementary Fig. S4a), while the LKB1-AMPK axis was usually considered to be an important tumor suppressor [55, 56] indicating that cancer would be an appropriate system to elucidate the biological function of MDK and LKB1-AMPK. In The Cancer Genome Atlas (TCGA) database, we found MDK is the highest upregulated growth factor in different types of cancer, even higher than that of established growth factors such as VGF, EGF, and TGFB1 (Fig. 5a, b, Supplementary Fig. S4b).

To further examine the expression of MDK in practical samples, we collected 36 pairs of liver cancer tissues and adjacent noncancer tissues. MDK expression was significantly upregulated in the cancer tissues compared to its expression in the adjacent normal tissues (Fig. 5c, d, Supplementary Fig. S4c). Similar result was obtained from immunohistochemistry (IHC) assay to the tissue microarray assay (TMA), containing 75 pairs of liver cancer and adjacent normal tissue samples (Fig. 5e, f, Supplementary Fig. S4d). Notably, higher MDK expression correlated with poor prognosis in both the TCGA liver hepatocellular carcinoma (LIHC) and kidney renal clear cell carcinoma (KIRC) cohorts (Fig. 5g, h). Taken together, MDK is highly expressed in most cancers, which suggests that MDK plays important functions in cancer progression.

### Midkine promotes cancer cell proliferation, invasion and tumorigenesis

To clarify the function of MDK in tumorigenesis, we performed both loss-of-function and gain-of-function analyses of MDK in different cancer cell lines. MDK was highly expressed in most cancer cell lines compared to its expression in immortalized normal human liver cells and mammary epithelial cells; however, in some cancer cell lines, MDK expression was almost negligible (Supplementary Fig. S5a, b). This negative expression of MDK may be due to gene methylation in the *MDK* genomic region or transcriptional regulation in particular cancer cells instead of genome deletion (Supplementary Fig. S5c).

**Fig. 6:**
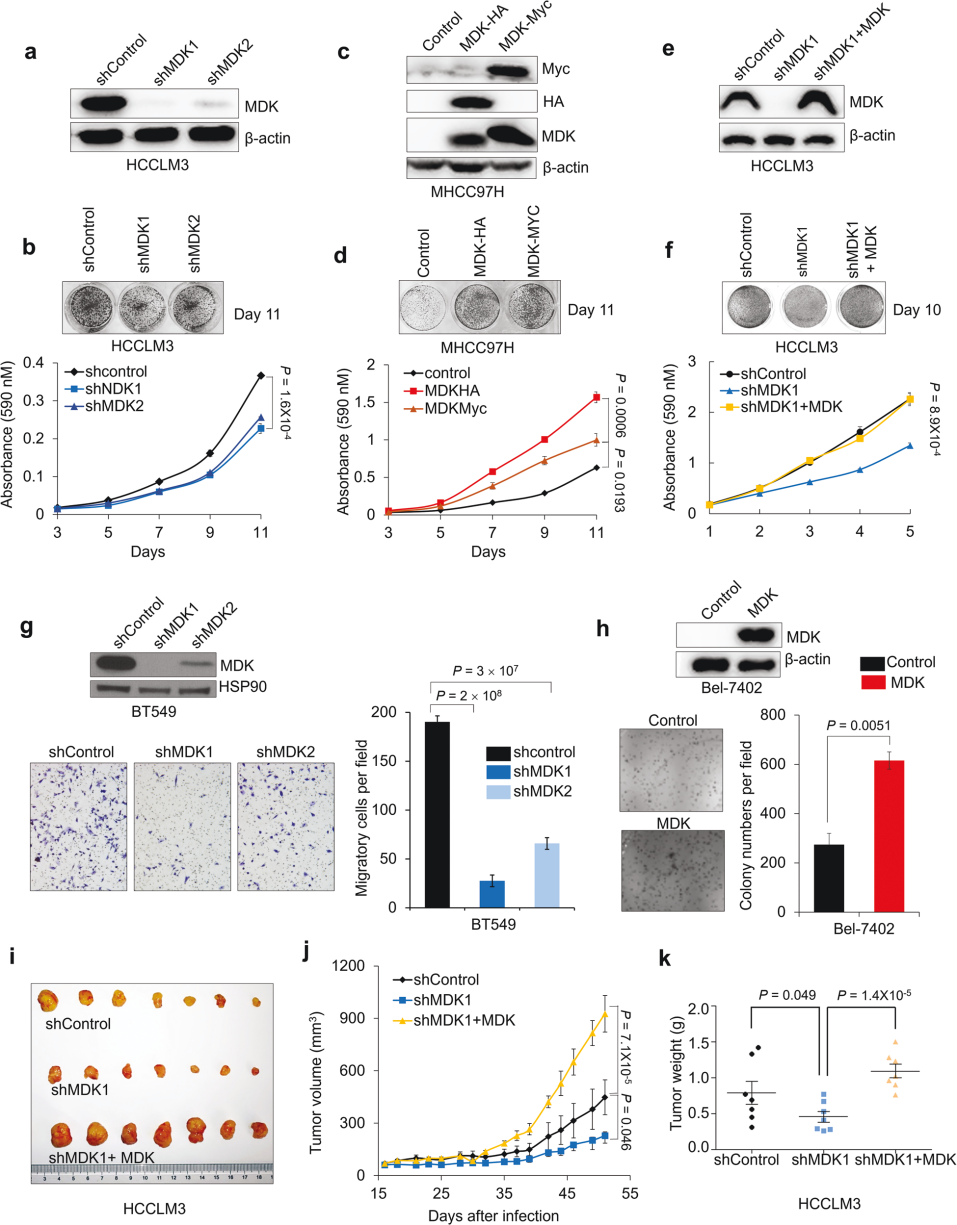
Midkine promotes cancer cell proliferation, invasion and tumorigenesis. **a**–**b** Western blotting of MDK and β-actin in HCCLM3 cells transduced with two independent MDK shRNAs (**a**) and representative images and growth curves of the HCCLM3 cells with MDK knocked down (**b**). **c**–**d** Western blot analysis of MDK, Myc, HA, and β-actin in the MDK-transduced MHCC97H cells (**c**) and representative images and cell growth curves of MHCC97H cells overexpressing MDK (d). **e**–**f** Western blot analysis of MDK and β-actin in the HCCLM3 cells transduced with MDK shRNA1 and restored MDK in the MDK-knockdown cells (**e**) and representative images and cell growth curves of the HCCLM3 cells transduced with MDK shRNA1 and restored MDK in the MDK-knockdown cells (**f**). **g** Western blotting of MDK and HSP90 in the BT549 cells transduced with two independent MDK shRNAs and representative images and invaded cell numbers of BT549 cells with MDK knocked down. *n* = 3 wells per group. Scale bar, 200 μm. **h** Western blotting of MDK and β-actin in the MDK-overexpressing Bel-7402 cells and representative images and clone numbers of the Bel-7402 cells with restored MDK expression. *n* = 3 wells per group, Bar = 200 μm. **i**–**k** Tumor images (**i**), growth curve (**j**) and weight (**k**) after subcutaneously injecting mice with HCCLM3 cells transduced with MDK shRNA or reconstituted MDK in MDK-knockdown cells.

Considering these expression assessment results, we generated MDK-transduced and short hairpin RNA (shRNA) knockdown cell lines (Fig. 6a, e–g, Supplementary Fig. S5d). Two independent MDK shRNAs both decreased the proliferation of HCCLM3 and HepG2 cells (Fig. 6b, f, Supplementary Fig. S5e). In contrast, transducing MDK expression vector in MHCC97H and Bel-7402 cells increased their proliferation (Fig. 6c, d, Supplementary Fig S5f). In addition, restoring MDK expression in MDK-knockdown HCCLM3 and HepG2 cells recovered their proliferation ability (Fig. 6e, f, Supplementary Fig. S5d, e). We also examined the effect of MDK on cell motility and anchorage-independent growth. Knocking down MDK expression significantly decreased the invasion ability of BT549 cells (Fig. 6g), and the overexpression of MDK increased the colony-forming ability of Bel-7402 cells in soft agar (Fig. 6h). However, MDK-overexpressing cells did not affect wound healing migratory ability (Supplementary Fig. S5g). To explore the function of MDK in tumor growth in vivo, and mice with MDK shRNA-expressing cancer cells produced smaller tumor, measured by volume, and lighter tumor, measured by weight, throughout the experiment than mice transplanted with the control shRNA-infected cells or MDK-reconstituted HCCLM3 cells (Fig. 6i–k). In contrast, MDK-overexpressing MHCC97H cells accelerated tumor growth and tumor weight in vivo (Supplementary Fig. S5h, i). Furthermore, the tumors formed by MHCC97H cells overexpressing MDK exhibited downregulated AMPKα phosphorylation compared with the tumors formed by control MHCC97H cells (Supplementary Fig. S5j). Taken together, these results indicate that MDK promotes the proliferation and tumorigenesis of human cancer cells.

### Midkine promotes cancer progression by negatively regulating LKB1-AMPK signaling

To investigate the role of AMPK in MDK-modulated cell proliferation, we performed a colony-forming assay under normal and low-glucose conditions. As expected, MDK-overexpressing cells showed an accelerated proliferation rate under both conditions, and we also observed that, compared to the effects under normal conditions, the overexpression of MDK significantly promoted cell proliferation under low-glucose conditions (Supplementary Fig. S6a, b). However, knocking down MDK did not alter the mitochondrial oxygen consumption rate (OCR) or extracellular acidification rates (ECARs) (Supplementary Fig. S6c, d), thus indicating that MDK did not affect glycolysis or mitochondrial oxidative phosphorylation.

**Fig. 7:**
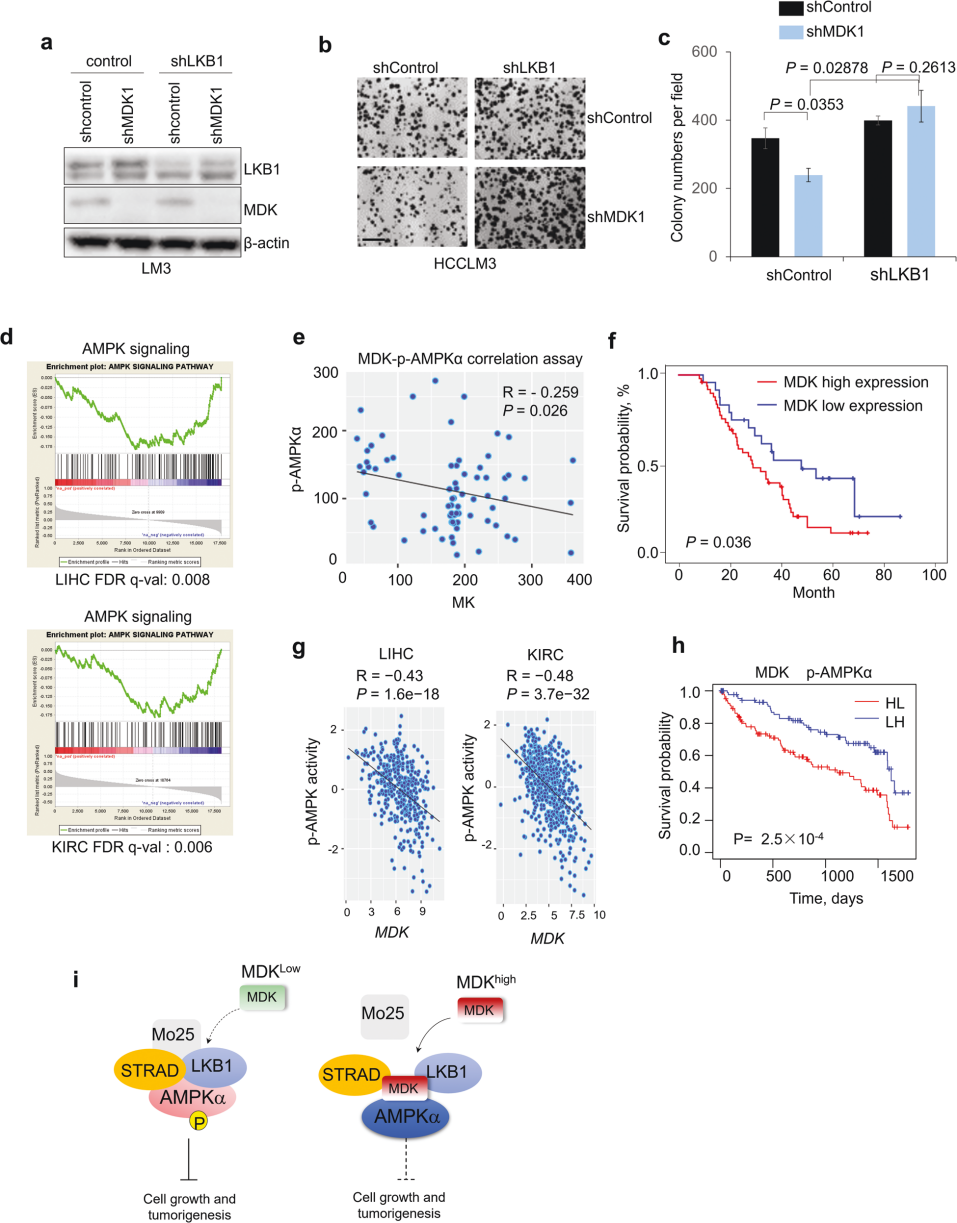
Midkine promotes cancer progression by negatively regulating AMPK signaling. **a** Western blotting of MDK, LKB1 and β-actin from HCCLM3 cells transduced with LKB1 shRNA or in combination with MDK shRNA. **b**–**c** Colony-forming assay of the HCCLM3 cells transduced with LKB1 shRNA or in combination with MDK shRNA. Images (**b**) and quantification (**c**) of colony formation. *n* = 3 wells per group. Scale bar, 200 μm. **d** GSEA results showing the negative correlations between MDK and the AMPK signaling pathway based on the TCGA LIHC and KIRC cohorts. Genes in the RNA-seq data were ranked by the Pearson coefficients of the correlations between the genes and *MDK*, and the ranked gene list was utilized as the input for the GSEA software program. **e**–**f** Scatter plots showing the inverse correlation of MDK with p-AMPKα expression and MDK expression in human hepatocellular carcinoma tumors (**e**), Kaplan–Meier survival curves of HCC patients with data stratified by MDK expression levels (**f**) (*n* = 74). **g** Scatter plots showing the inverse correlation of MDK AMPK activity in the TCGA LIHC and KIRC cohorts (LIHC n = 371, KIRC n = 531). Gene expression was obtained from RNA-seq data from the TCGA. AMPK activity was estimated by the expression of their downstream target genes. Statistical significance in (e-g) was determined by Pearson correlation test. R: Pearson correlation coefficient. R, Spearman rank correlation coefficient. **h** Kaplan–Meier survival curves of the TCGA KIRC patients with data stratified by the expression of *MDK* and the activity of AMPK. HL: MDK is high, AMPK activity is low*;* LH: MDK is low, AMPK activity is high. Median expression/activity levels were utilized as the thresholds for high and low separation. **i**. Schematic illustration of the MDK mechanisms of action: high MDK expression depolymerizes the LKB1-STRAD-Mo25 complex and subsequently suppresses the activity of AMPK signaling in human cancers.

Furthermore, to determine whether MDK decreases LKB1 activity to suppress AMPK activation and thus contributes to cell proliferation, we expressed LKB1 shRNA and CAMKKβ shRNA in MDK-depleted HCCLM3 cells. Knocking down LKB1, but not CAMKKβ, reversed the inhibitory effects of MDK shRNA on cell proliferation and colony formation (Fig. 7a–c, Supplementary Fig. S6e–g). In addition, reconstitution of LKB1 in its deficient Hela cells, significantly inhibited the cell proliferation caused by MDK overexpression (Supplementary Fig. S6h–j).

To understand whether MDK affects AMPK-related signaling pathways in different cancers, we performed a gene expression correlation-based gene set enrichment analysis (GSEA). The results showed that MDK expression is negatively correlated with the AMPK signaling pathway in several different cancers, such as liver cancer, kidney cancer, and breast cancer (Fig. 7d, Supplementary Fig S7a, b). The IHC analysis of the HCC tissues revealed that MDK protein expression is negatively correlated with p-AMPKα expression (Fig. 7e), and high MDK expression correlates with poor prognosis for HCC patients (Fig. 7f and Supplementary Table 1).

Next, to investigate the relevance of our findings to human cancer, we analyzed gene expression data from TCGA and Gene Expression Omnibus (GEO) datasets. We found a significant negative correlation between *MDK* and the activity of AMPK in the TCGA database (Fig. 7g, h, Supplementary Fig. S7b, c; see the Methods section for the estimation of AMPK activity). Next, we examined the prognostic value of *MDK* with AMPK activity using the TCGA dataset of KIRC tumors from 531 patients. The patients with simultaneous high expression levels of *MDK* and low AMPK activity had shorter overall survival in the KIRC cohort (Fig. 7h). In summary, targeting the high expression of MDK may provide therapeutic benefits in human cancers.

## Discussion

MDK is a growth factor belonging to the pleiotrophin family. This definition, most of study in MDK inspired seeking for receptor proteins and resulted in several receptors identified including ALK, LRP1, Notch2 and PTPξ [45, 46, 57–60]. MDK was also found to be internalized by cells after secretion, but this point has been largely ignored in previous MDK functional studies. In the present study, we not only confirmed the intracellular transport of MDK (Fig. 1b, i–l) but also show the high efficiency of this translocation (Supplementary Fig. S1a, b). In addition, heparin treatment caused a large decrease in intracellular MDK (Fig. 1j–l), indicating that most intracellular MDK was the result of extracellular transport. These results suggested that MDK has highly efficient transportability, which enables it to play important roles in the cytoplasm, which is supported by our finding that MDK suppresses AMPK activity in an intracellular dependent manner (Fig. 1i-l) Besides, the detailed internalization mechanism of MDK also deserves future investigation.

In this study, we reveal previously undescribed functions of intracellular MDK to modulate LKB1 activity by disrupting the formation of the LKB1-STRAD-Mo25 complex via directly associating with LKB1 and STRAD (Figs. 2b-h, 4a-e, Supplementary Fig. S3a, b, e, f) and decrease the activity of LKB1 substrate, AMPK (Fig. 1e–h) [23]. Thus, we elucidated a new intracellular function and molecular mechanism of MDK (Fig. 7i), which will inform us as we further investigate whether this mechanism commonly mediates the activity of other proteins. Except AMPK, LKB1 phosphorylates other AMPK family proteins, such as MARKs and SIKs to inhibit tumor progression [61, 62]. It deserves to further explore whether MDK regulates other AMPK family members through LKB1. Besides, several posttranslational enzymes are in the MDK associated protein complex, such as USP10, indicating posttranslational modification of MDK to regulate its stability and activity. Indeed, a recent study reported that USP12 deubiquitinates and stabilizes MDK and this stabilization promotes breast cancer angiogenesis [42]. These results may inspire new function and modulation mechanism study of MDK, and clarify more comprehensively how MDK regulates diverse physiological processes.

The expression of MDK has been reported to be upregulated in diverse types of cancer [63], which was confirmed in our study (Fig. 5a–f, Supplementary Fig. S4). The elevated expression of MDK was accompanied by decreased patient survivals (Fig. 5g, h, i, j), suggesting that MDK serves as a prognostic marker. In this study, we found that MDK suppresses the activation of AMPK (Fig. 1d–i, Fig. 3a–f). Although AMPK is usually considered to be a tumor suppressor, several studies have also reported that AMPK promotes tumor progression by protecting tumor cells under energy stress conditions [18]. These findings indicate that some individual cases may manifest specific regulatory mechanisms. Here, we also found that the expression of MDK was upregulated overall, but some individuals showed the opposite expression trend (Fig. 5b–f, Supplementary Fig. S4c, d). It will be interesting to determine the AMPK phosphorylation level and tumor development stage in low-MDK tumors. MDK has been widely presumed to be a diagnostic marker of several different cancers, but the ambiguous regulatory molecular mechanism has postponed its utilization. Here, we elucidate the molecular mechanism by which MDK modulates tumor progression, including the suppression of AMPK signaling, to provide more clues to advance the clinical application of MDK in cancer diagnosis and prognosis.

## Supplementary information

Supplementary File

Supplementary Figure S1

Supplementary Figure S2

Supplementary Figure S3

Supplementary Figure S4

Supplementary Figure S5

Supplementary Figure S6

Supplementary Figure S7

Original WB images

Checklist

## Data Availability

All data needed to evaluate the conclusions in the paper are present in the paper. Additional data related to this paper may be requested from the corresponding author.
