# Supplementary material for: Midkine noncanonically suppresses AMPK activation through disrupting the LKB1-STRAD-Mo25 complex
Source: Cell Death Dis. 2022 Apr 29;13(4):414. doi: 10.1038/s41419-022-04801-0 (PMC9054788; doi:10.1038/s41419-022-04801-0)
Supplement: Supplementary file 1 — Supplementary File [file 41419_2022_4801_MOESM1_ESM.pdf]

Supplementary Information

Midkine noncanonically suppresses AMPK activation through disrupting the LKB1-STRAD-Mo25 complex

Tian Xia[1], Di Chen[1], Xiaolong Liu[1], Huan Qi[1], Wen Wang[1], Huan Chen[1,4], Ting Ling[1,4], Wuxiyar Otkur[1], Chen-Song Zhang[2], Jongchan Kim[3], Sheng-Cai Lin[2] & Hai-long Piao[1,4,*]

[1]CAS Key Laboratory of Separation Science for Analytical Chemistry, Dalian Institute of Chemical Physics, Chinese Academy of Sciences, Dalian 116023, China.

[2]State Key Laboratory for Cellular Stress Biology, School of Life Sciences, Xiamen University, 361102 Fujian, China.

[3]Department of Life Sciences, Sogang University, Seoul 04107, Republic of Korea

[4]University of Chinese Academy of Sciences, Beijing 100049, China.

*Correspondence: Hai-long Piao, hpiao@dicp.ac.cn

Materials and Methods

Cell lines and culture conditions

The HCC cell lines were purchased from the cell bank of Committee on Type Culture Collection of the Chinese Academy of Sciences (CTCC, Shanghai, China). Immortalized human liver cell THLE-2 and breast cancer cells MCF10A, MCF7, T47D, BT549, MDA-MB-231, MDA-MB-157, MDA-MB-436, MDA-MB-468 were obtained from ATCC (American Type Culture Collection) and cultured under conditions specified by the provider. MHCC-97H MCF7, T47D, BT549 and A549 cell lines were maintained in RPMI 1640 medium (GIBCO, USA). 293T, Huh7, BEL-7402, Hep3B, HepG2, SMMC-7721, HCC-LM3 and MCF10A cells were cultured in DMEM medium (GIBCO, USA). MDA-MB-231, MDA-MB-157, MDA-MB-436, MDA-MB-468 cells were cultured in L15 medium (Gibco, USA). The medium was mixed with 10% FBS (GIBCO, USA) and 1% penicillin/streptomycin (Thermo) before use. THLE-2 cell line was cultured in bronchial epithelial cell growth medium (BEGM, Lonza/Clonetics Corporation, Walkersville) supplemented with 200 ng/ml epidermal growth factor (EGF), 500 ng/ml hydrocortisone, 100 ng/ml choleratoxin, 10 μg/ml insulin, 10% FBS and 1% P/S. All cells were cultured at 37°C with 5% CO2 in a humidified incubator.

Constructs

For transient transfection, MDK, MDKDel, AMPKα1, AMPKβ1, AMPKγ1, LKB1, CAMKKβ, full length and truncated LKB1 coding sequences were fused with HA, Flag or Myc tag sequence by PCR and then subcloned to pCDNA3.1 vector in appropriate restriction endonuclease sites. Otherwise, these coding sequences were subcloned to the entry vector pDONR201 using Gateway System of Invitrogen company, and then recombined into gateway-compatible destination vectors for the transient expression of native or SFB-tagged proteins.

For the stable expression of proteins and shRNAs in cells, lentivirus packaging system was used. Coding sequences of MDK, MDK_Del, LKB1 and AMPKα1 were subcloned to pLoc-RFP vector in appropriate restriction endonuclease sites, or recombined to gateway destination vectors. Lentiviral shRNAs were cloned in pLKO.1 within the

AgeI/EcoRI sites at the 3'end of the human U6 promoter. The targeted sequences were shown in the table above.

Transfection and Lentivirus infection

HEK293T cells was used for transfection and lentivirus packaging. Plasmids and PEI (Polyethylenimine, Polysciences 24765) were mixed at 1:4 ratio (w/w) in Opti-MEM medium (Gibco), and then added to 293T cell medium. Protein expression was tested about 48 hours after transfection.

For lentivirus packaging, the protein or shRNA expressing lentivirus vectors were transfected into 293T cells together with packaging vectors psPAX2 and pVSVG using PEI. Virus containing medium was harvested 48 hours after transfection and filtered with 0.45 μm membrane to remove cell debris, then the virus containing medium was used to culture target cells for 8-24 hours. Puromycin or blasticidine were used to screen positive cells, depending on the lentivirus vector resistant gene.

Cell proliferation and cloning formation assay

For cell proliferation assay, cells (3000 per well) were seeded in 6-well or 12-well plates, then cultured under 37°C and 5% CO_2 condition. At each time point, cells were stained with crystal violet (0.1% w/v, in 10% methanol) for 1 hour at room temperature, then the cells were washed by PBS for 3 times to remove extra crystal violet. Finally, the crystal violet in stained cells was dissolved in 10% acetic acid, and the OD590 value was measured.

For soft agar colony formation assay, cells (5000 per well) were suspended in medium containing 0.4% agar and overlaid on 0.7% agar in 6-well plates. Extra liquid medium was added on the surface of ager to keep top layer moist. The plates were incubated for 3 weeks in a 37 °C incubator, followed by crystal violet staining for 2 hours. After extra crystal violet was taken off by PBS, pictures of the cell colony were taken and the clone numbers were counted with the help of Image J.

Energy stress treatment

For Western Blot assay, cells were seeded on 60 mm dishes 24 hours before treatment. After the cells adhered to dish bottom, the medium was vacuumed and completely removed by washing with PBS. After PBS was vacuumed, glucose deficient medium

or 2-DG (2-Dexoy-D-Glucose) containing medium was used to culture the cells. At the end of energy stress treatment, the medium was vacuumed, and cells were washed once with PBS. After PBS was removed, the dishes were immediately put in liquid nitrogen, then the cell protein was extracted and test by WB.

For 2-D colony formation assay, cells (500 per well) were seeded in 6-well plates. 24 hours after cell adherent, medium with normal of low concentration of glucose was applied to culture the cells for 2-3 weeks until visible colonies formed. Medium was replaced every 3 days. Finally, the cells were stained with crystal violet (0.1% w/v, in 10% methanol) for 1 hour at room temperature, then the cells were washed by PBS for 3 times to remove extra crystal violet. The colony numbers were counted with the help of Image J.

Midkine conditioned medium treatment

To obtain MDK conditioned medium, MHCC97H cells that stably expressed ectopic MDK were seeded in 100 mm dishes and cultured until they reached over 90% confluency. Then the cells were further cultured in fresh medium for 24 hours to make sure MDK secretion. Then the MDK containing medium was filtered with 0.22 μm membrane to remove cell debris and used to incubate cells.

Xenograft Studies

The indicated cells (1×10^6) were suspended in 100 μl PBS, and subcutaneously injected into flanks of male nude mice (Balb/c, 4-6 weeks old). Tumor size was measured every 2 or 3 days with a caliper and the tumor volume was determined using the formula: (widest diameter × smallest diameter^2)/2. The mice were sacrificed at the end of the studies and the tumors were dissected and weighted, followed by keeping in liquid nitrogen for protein extraction.

Clinical specimens

36 pairs of HCC and normal liver tissue samples were obtained from the first affiliated hospital of Dalian Medical University. All samples were collected with the informed consent of the patients. The experiments were approved by Research ethics committee at the first affiliated hospital of Dalian Medical University. Human HCC Tissue Microarrays were purchased from SHANGHAI OUTDO BIOTECH Company.

Immunoblotting assay

Cells were scraped from plates and lysed in RIPA buffer [50 mM Tris-Cl (PH7.4), 150 mM NaCl, 1 mM EDTA (PH8.0), 0.25% DOC (deoxycholic acid), 10% glycerol, 1% Nonidet P40 and 1% Triton-X100] supplemented with protease inhibitors (Bimake) and phosphatase inhibitors (Bimake). The cell lysates were clarified by centrifugation at 13000 r.p.m. for 15 min at 4 °C. The protein samples from tumors were homogenized with glass beads and then lysed in RIPA buffer with protease/phosphatase inhibitors. The protein concentrations of the lysates were measured using EASY II Protein Quantitative Kit (Transgene). SDS-PAGE was performed for equal amounts of protein per sample, followed by transfer to a PVDF membrane (Millipore). Then the proteins on the PVDF membrane were immunoblotted with indicated antibodies listed in the Extended Method Table.

Immunoprecipitation and S-protein pull down

Cells were scraped from dishes and lysed with NETN buffer [20 mM Tris-Cl (PH8.0), 100 mM NaCl, 1 mM EDTA (PH8.0), 0.5% Nonidet P40] containing protease and phosphatase inhibitors. The cell lysates were clarified by centrifugation at 13000 r.p.m. for 15 min at 4 °C.

For immunoprecipitation, the cell lysate was pre-cleared with normal IgG which had been bound to Protein A/G Agarose (Thermo) or Protein A/G magnetic Beads (Bimake) for 1 hour at room temperature. The pre-cleared cell lysate was then incubated with indicated antibodies which had been bound to Protein A/G Agarose or Protein A/G magnetic Beads for 1 hour at room temperature. After incubating with the cell lysate, the IgG/antibody bound Protein A/G agarose or magnetic beads were washed 4 times with NETN buffer to remove non-specific binding proteins. Then 1 X SDS-PAGE Loading buffer was added to the Protein A/G agarose or magnetic beads. Subsequently Western Blots were performed as described above.

For S-protein pull down, cell lysates were incubated with S-protein agarose (Millipore) at 4 °C for 4 hours. Then the agarose was washed 4 times with NETN, followed by adding 1 X SDS-PAGE Loading buffer and being tested by Western Blot.

RNA isolation and Q-RT-PCR

RNA of cell samples was isolated with RNAiso Plus reagent (TaKaRa). The concentrations of RNA were determined with NanoDrop (Thermo). Reverse transcription PCR was performed using PrimeScript^{TM} RT Master Mix (TaKaRa) to obtain cDNA as template for Q-RT-PCR. Q-RT-PCR was performed using StepOnePlus and the DNA double‑strand‑specific reagent SYBR‑Green I for detection (Roche) in the CFX-96 instrument (Bio-Rad). Primers for Midkine transcript detection were as followed:

F: 5'-GACCATCCGCGTCACCA-3'

R: 5'-TCCAGGCTTGGCGTCTAGTC-3'

Immunohistochemistry

The samples were fixed with 4% PFA, and embedded with paraffin. Standard IHC staining procedures were performed according to the instructions of IHC Kit. MDK (1:50) and p-AMPKα Thr172 (1:100) were used as the primary antibodies. EDTA and Citrate solution were used for antigen retrieval depend on antibody instruction. H-score was used to assess the staining intensity.

Cell invasion and migration ability assay

Cell invasion ability test was performed using Transwell plate coated with Matrigel (BD) according to the manufacture's instructions. Briefly, after serum starvation overnight, cells (2×10^5 per well) were seeded in Transwell Matrigel plate and incubated for 30 hours. Finally, the invasive cells were visualized by crystal violet staining. Photograph of the invaded cells were taken by microscope and analyzed by Image J.

Wound healing assay was used to test cell migration abilities. Cells (1×10^6 per well) were seeded in 6-weel plates. After the cells reached approximate 98% confluency, they were cultured in serum free medium containing Mitomycin (4 μg/ml) for 6 hours to postpone cell proliferation. Next, the cells were scratched with a sterile 10μl pipette tip and incubated in fresh serum-free media after the cell debris was removed by PBS. The width of gap was photographed and measured at the indicated time points with Image J.

Statistical analysis

Data representative of two or more independent experiments. Statistical analysis was

performed using Student's *t*-test (unpaired, two-tailed) to compare two groups of independent samples, and a difference with P value <0.05 was considered statistically significant. Bars and error represent mean ± standard deviations (SD) of replicate measurements. All of the relative protein expression was normalized by ImageJ (1.51j8). The survival curve was achieved using Kaplan Meier estimator. The correlation analysis between MDK and p-AMPKα was performed with Pearson Correlation Coefficient. The detailed methods for TCGA data analysis and pathway enrichment were presented in the Computational analysis part below.

Computational analysis

TCGA and HPM data: The normalized RNA-seq data together with clinical survival information in TCGA (1) were downloaded by the TCGAbiolinks R package (2). The expression matrix for each cancer type was further transformed by the voom algorithm in the limma R package (3). The human proteome map (HPM) (4) protein level expression data were downloaded from http://www.humanproteomemap.org/download.php.

Survival analysis: For a single gene, patients were separated into two groups with expressions higher or lower than the median expression of the gene, then we utilized the log-rank test to compare the overall survival time between groups, and survival curves were created based on Kaplan Meier estimator. For two items (*MDK* and *PRKAA1* or AMPK), patients were separated into four groups according to the expressions of two items-“HH” group: both items are high, “HL” group: the first one was high, while the other was low, “LH” group: the first one was low while the other was high, “LL” group: both were low. To determine whether the inverse correlations between MDK and AMPK can cooperatively impact the prognosis, we utilized the log-rank test to compare the overall survival time between “HL” and “LH” groups, and survival curves were created based on Kaplan Meier estimator.

Pathway enrichment analysis: Initially, we calculated the Pearson correlation coefficients between MDK and all other genes according to their mRNA expression levels in the RNA-seq datasets. The genes with absolute value s of Pearson correlation coefficients larger than threshold were considered as MDK correlated genes. Then, we

utilized Fisher-Exact to examine the overlap significance between MDK correlated genes and pathway genes. Furthermore, to obtain a genome-wide perspective of MDK impacts on pathways, we also ranked all the genes in the RNA-seq data based on the Pearson correlation coefficients and utilized the ranked genome-wide gene list as the input of the GSEA software (5), and performed GSEA-based pathway enrichment. Pathway genes were obtained from Kyoto Encyclopedia of Genes and Genomes (KEGG) (6), https://www.kegg.jp/.

Disease enrichment analysis: We calculated the Pearson correlation coefficients between MDK and all other proteins according to their protein expression levels in the HPM dataset. The proteins with absolute value s of Pearson correlation coefficients larger than threshold were considered as MDK correlated. Then, we utilized Fisher-Exact to examine the overlap significance between MDK correlated proteins and pathway genes. Disease genes were obtained from Online Mendelian Inheritance in Man (OMIM) (7), https://omim.org/.

AMPK activity estimation: The AMPK activity was derived by performing principal component analysis on the gene expression data of 33 AMPK downstream genes (e.g., ACADL, ACADM, ACOX1, ACSL1, etc.), and the first principal component was used to quantify the activity of AMPK (8).

Reference

1. Cancer Genome Atlas Research N, Weinstein JN, Collisson EA, Mills GB, Shaw KR, Ozenberger BA, et al. The Cancer Genome Atlas Pan-Cancer analysis project. Nature genetics. 2013;45(10):1113-20.
2. Colaprico A, Silva TC, Olsen C, Garofano L, Cava C, Garolini D, et al. TCGAbiolinks: an R/Bioconductor package for integrative analysis of TCGA data. Nucleic Acids Res. 2016;44(8):e71.
3. Law CW, Chen Y, Shi W, Smyth GK. voom: Precision weights unlock linear model analysis tools for RNA-seq read counts. Genome Biol. 2014;15(2):R29.
4. Kim MS, Pinto SM, Getnet D, Nirujogi RS, Manda SS, Chaerkady R, et al. A draft map of the human proteome. Nature. 2014;509(7502):575-81.
5. Subramanian A, Tamayo P, Mootha VK, Mukherjee S, Ebert BL, Gillette MA, et al. Gene set enrichment analysis: a knowledge-based approach for interpreting genome-wide expression profiles. Proc Natl Acad Sci U S A. 2005;102(43):15545-50.
6. Kanehisa M, Furumichi M, Tanabe M, Sato Y, Morishima K. KEGG: new perspectives on genomes, pathways, diseases and drugs. Nucleic Acids Res. 2017;45(D1):D353-D61.
7. Hamosh A, Scott AF, Amberger JS, Bocchini CA, McKusick VA. Online Mendelian Inheritance in Man

(OMIM), a knowledgebase of human genes and genetic disorders. Nucleic Acids Res. 2005;33(Database issue):D514-7.
8. Yu L, Lu M, Jia D, Ma J, Ben-Jacob E, Levine H, et al. Modeling the Genetic Regulation of Cancer Metabolism: Interplay between Glycolysis and Oxidative Phosphorylation. Cancer research. 2017;77(7):1564-74.

Supplementary Figures

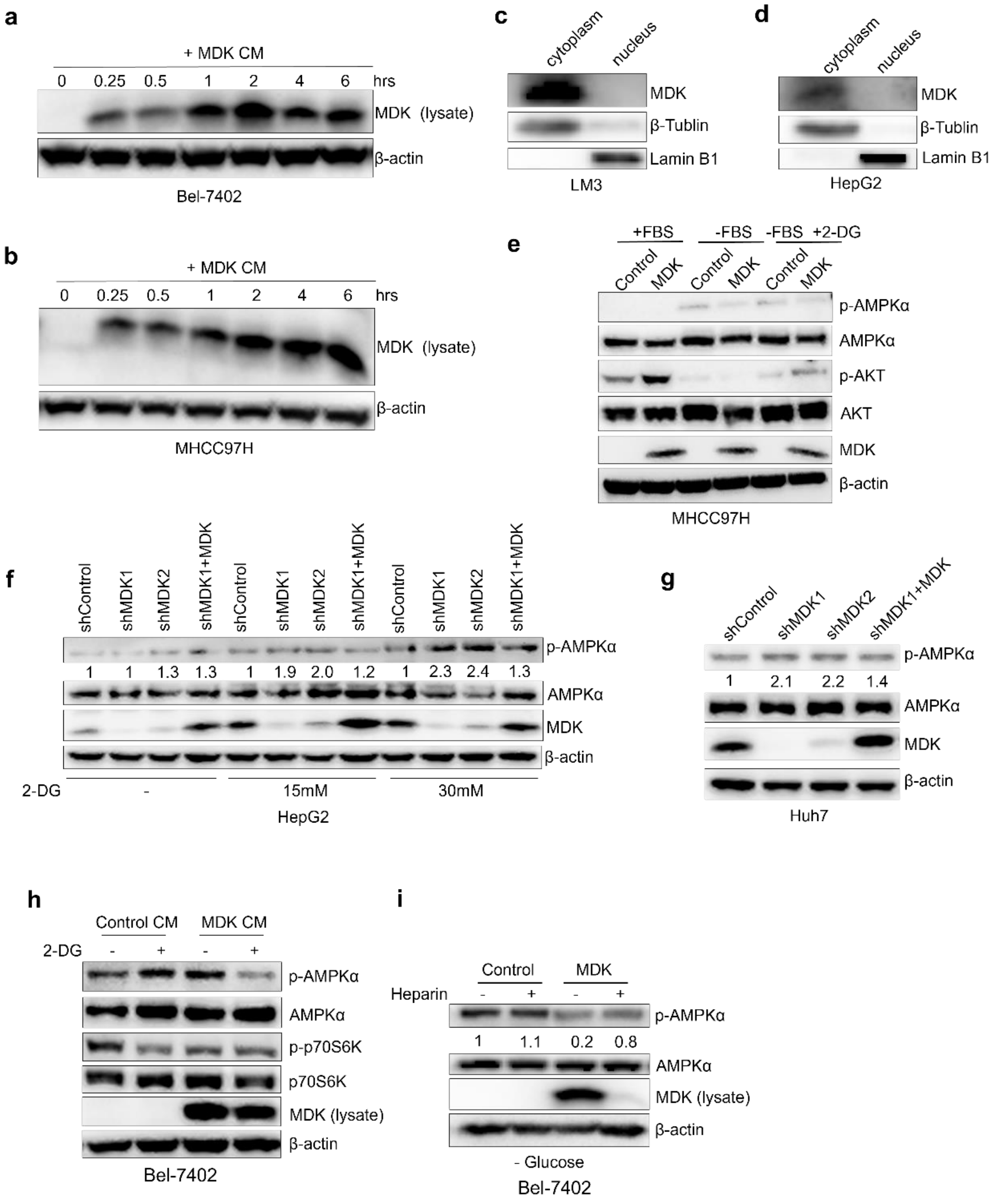

Supplementary Figure 1 | Midkine suppresses AMPK activation in an intracellular localization-dependent manner

a and b. Western blotting of MDK and β-actin in Bel-7402 (a) and MHCC97H (b) cells at different time points during CM treatment. CM from MDK-overexpressing MHCC97H cells.

c and d. Immunoblotting demonstrates the MDK distribution in the cytoplasm and

nuclear of of HCCLM3 (c) and HepG2 (d) cells.

e. Immunoblotting shows that MDK suppresses p-AMPKα phosphorylation in MHCC97H cells treated with FBS deprivation and combined with 10 mM 2DG for 2 hours.

f. MDK knocked-down HepG2 cells with two independent MDK shRNAs and the reconstituted MDK-knockdown cells , accompanied with the control cells were treated with different concentrations of 2DG (0 mM, 15 mM and 30 mM) treatment, the cell lysate was subjected to western blotting.

g. MDK expression ia negatively correlated to p-AMPKα Thr172 level in the Huh7 cells transduced with two independent MDK shRNAs and the reconstituted MDK-knockdown cells.

h. Immunoblotting of the Bel-7402 cells being subjected to a combination treatment with or without MDK CM and 10 mM 2-DG for 2 hours.

i. MDK overexpressing Bel-7402 cells and control cells was treated with or without heparin treatment under glucose starvation conditions for 2 hours, then western blotting was performed to detect the indicated proteins.

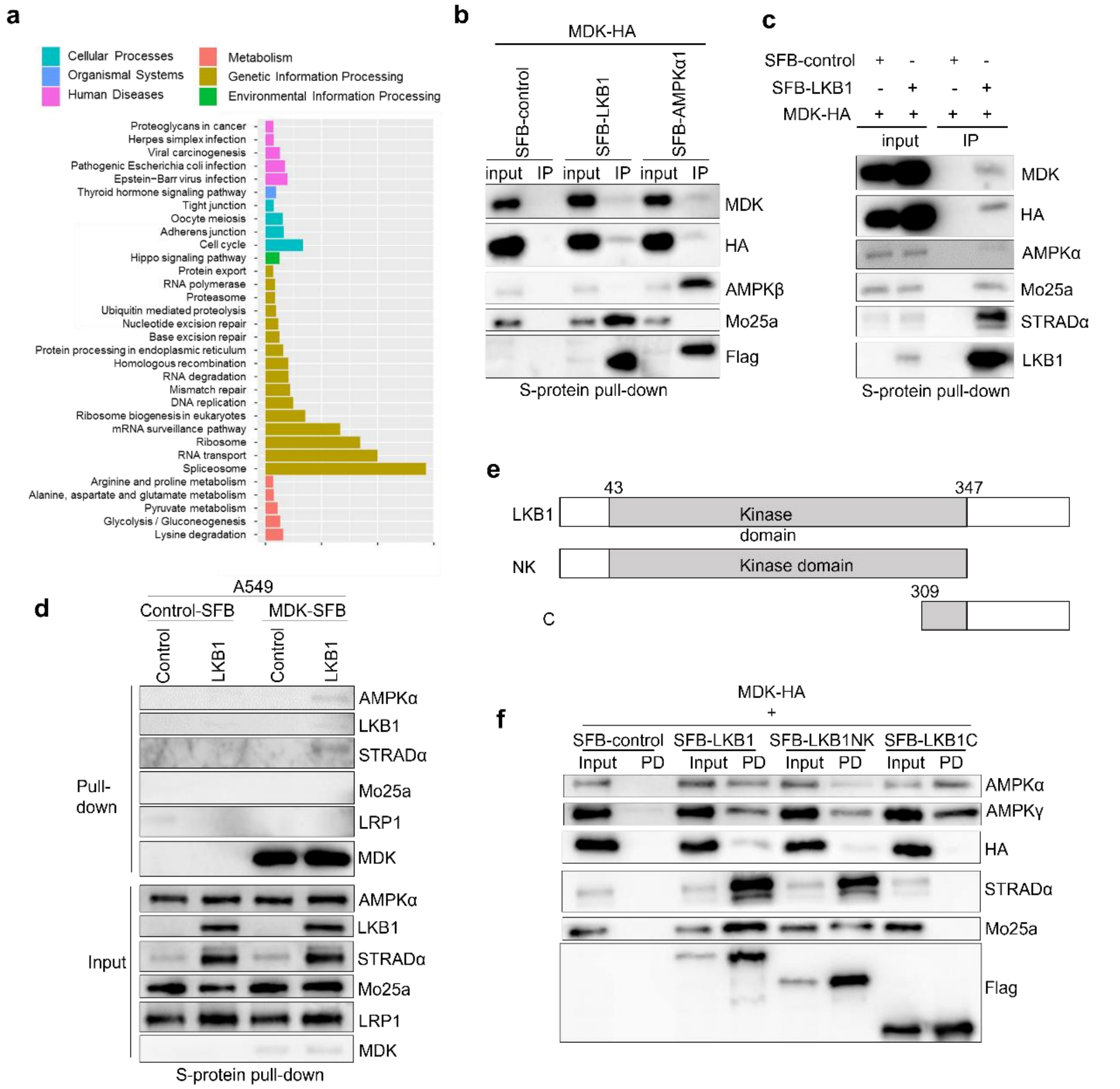

Supplementary Figure 2 | Midkine associates with AMPK subunits and its upstream regulating factors

a. Pathway enrichment of MDK-interacting proteins. The bar length is proportional to –log10 (P) (Fisher-Exact test). Different colors represent different pathway categories.

b and c. MDK associates with LKB1and AMPK subunits. The indicated constructs were expressed in HEK293T cells for 24 hours, and the cell lysates were subjected to pull-down assays with S protein beads.

d. MDK from A549 cells transduced with LKB1 was pulled down with S protein beads and the control cells and subjected to Western blot analysis with antibodies against AMPKα, LKB1, Mo25a, STRADα, LRP1 and MDK.

e and f. HEK293T cells were cotransfected with MDK-HA and SFB-tagged full-length

LKB1 or the LKB1-NK fragment or LKB1-C fragment, purified with S protein beads and subjected to Western blot analysis with antibodies against AMPKα, AMPKγ, HA, STRADα, Mo25a and FLAG.

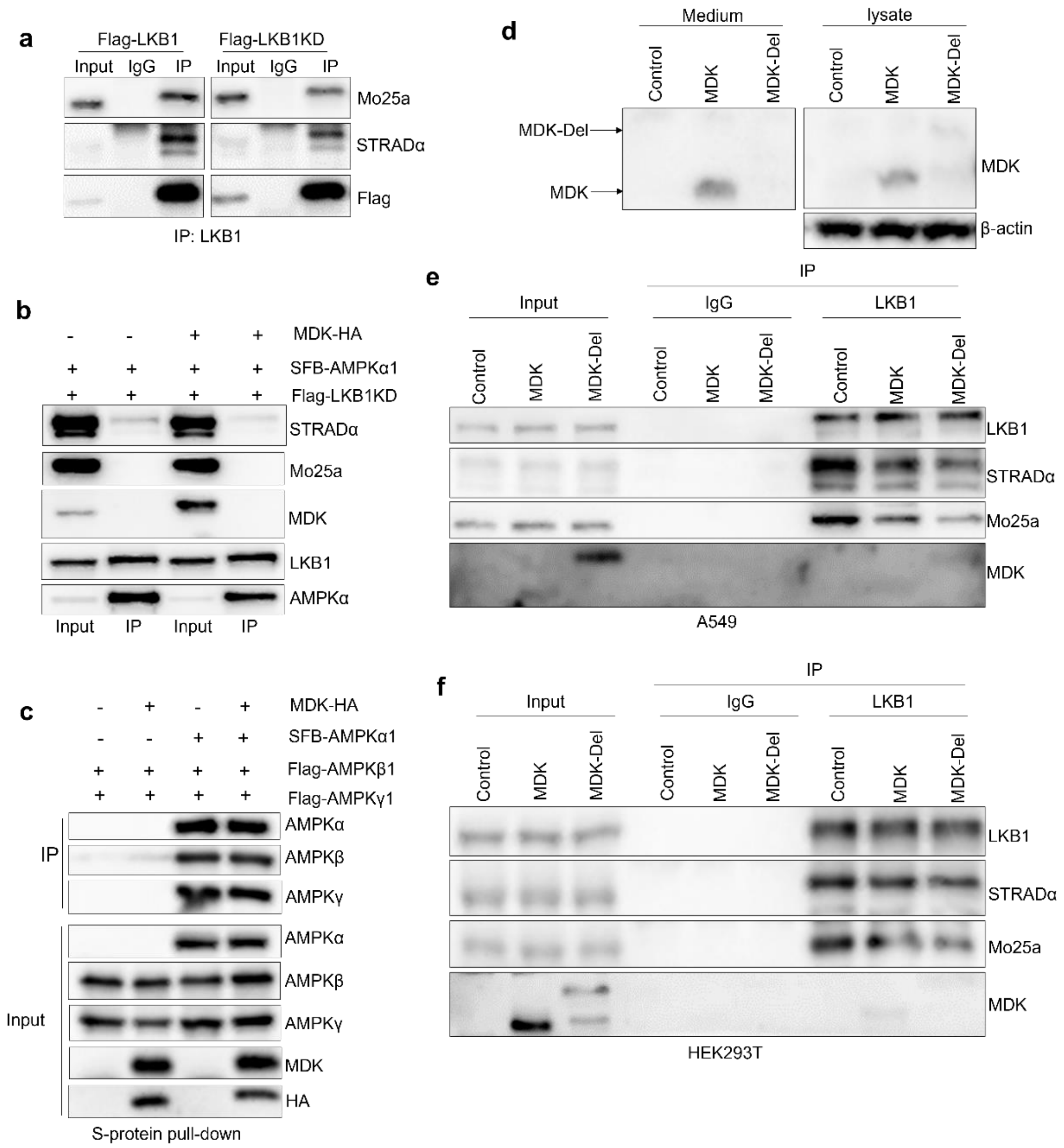

Supplementary Figure 3 | Midkine depolymerized LKB1-STRAD-Mo25 complex

a. HEK293T cells were transfected with FLAG-tagged LKB1 or FLAG-tagged LKB1-KD and coimmunoprecipitated with primary LKB1 or IgG antibodies and then subjected to Western blot analysis with antibodies against STRADα, Mo25a, and FLAG.

b. HEK293T cells were cotransfected with FLAG-tagged LKB1-KD and SFB-tagged AMPKα1 with or without MDK-HA and coimmunoprecipitated with FLAG primary antibody and the subjected to Western blot analysis with antibodies against STRADα, Mo25a, MDK, LKB1 and AMPKα.

c. MDK associates with AMPK subunits through AMPKα. The indicated constructs

were expressed in HEK293T cells for 24 hours, and the cell lysates were subjected to pull-down assays with S protein beads.

d. MDK and MDK-Del were expressed in HEK293T cells, and the expression of MDK in cell lysate and medium was tested by Western Blot.

e and f. MDK and MDK-Del were expressed in A549 (e) and HEK293T (f) cells, coimmunoprecipitation was performed using LKB1 primary antibody. The interactions between LKB1 with MDK, STRAD and Mo25 were tested by Western Blot.

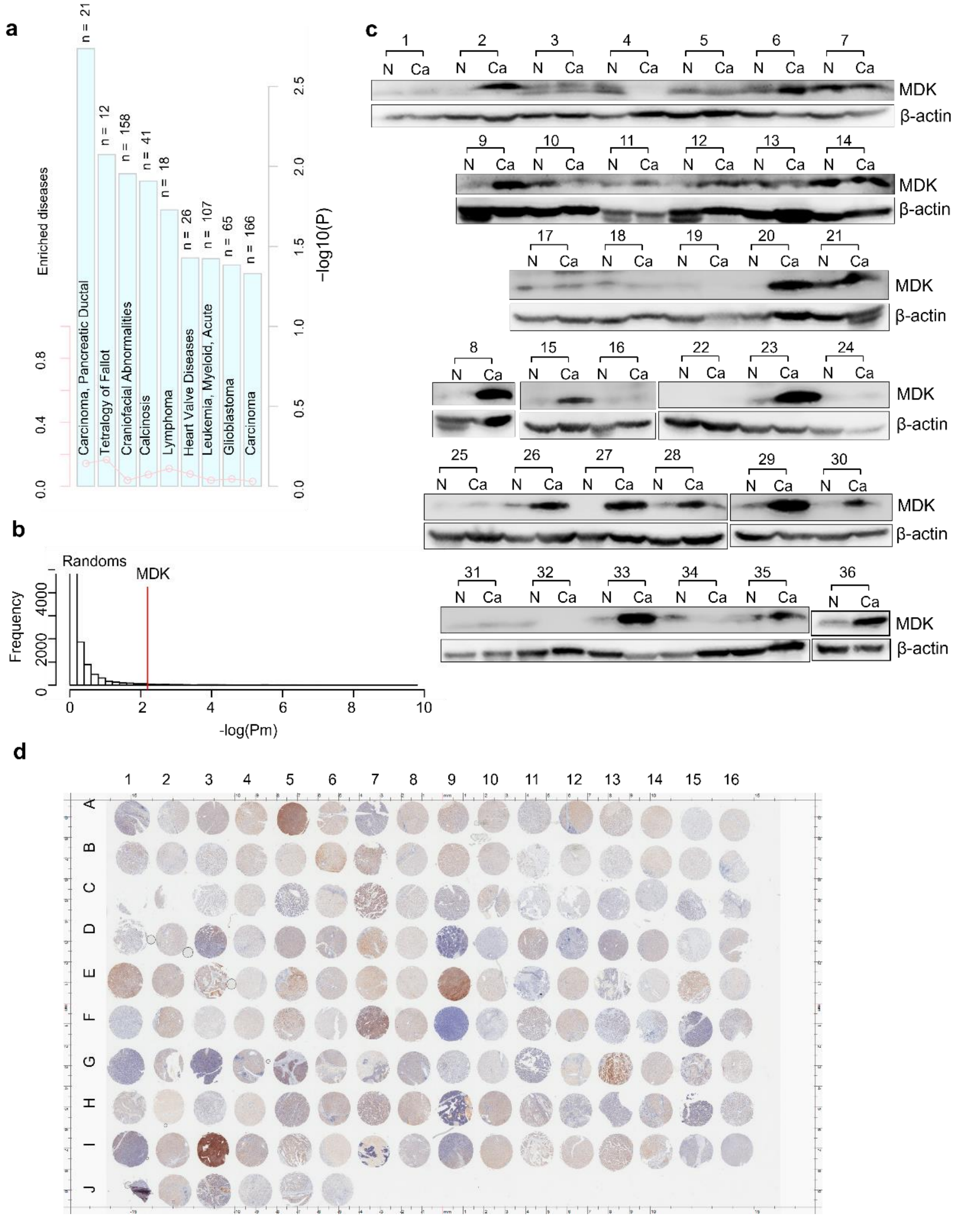

Supplementary Figure 4 | Midkine expression is upregulated in cancer

a. Enrichment of MDK-correlated proteins in diseases. The MDK-correlated proteins were identified based on the human proteome map (HPM) protein expression dataset (Spearman correlation test, absolute value of correlation coefficient > 0.6). The overlap of the MDK-correlated proteins and disease genes was assessed by Fisher's exact test; the bar length is proportional to the corresponding –log10 (P); and the point position

indicates the ratio of overlapping genes to disease genes.

b. A bar plot showing the distribution of the maximum P-value (Pm) of 10000 randomly selected genes considering the gene expression differences in the tumor and normal tissues of eight cancer types. The Pm is transformed by –log10. The red line annotates the corresponding results for MDK.

c. The expression of MDK in 36 pairs of matched adjacent nontumor (NT) and cancer (Ca) tissues was detected by Western blot analysis.

d. Immunohistochemical staining shows the MDK expression status in 75 pairs of paraffin-embedded adjacent nontumor and cancer tissues.

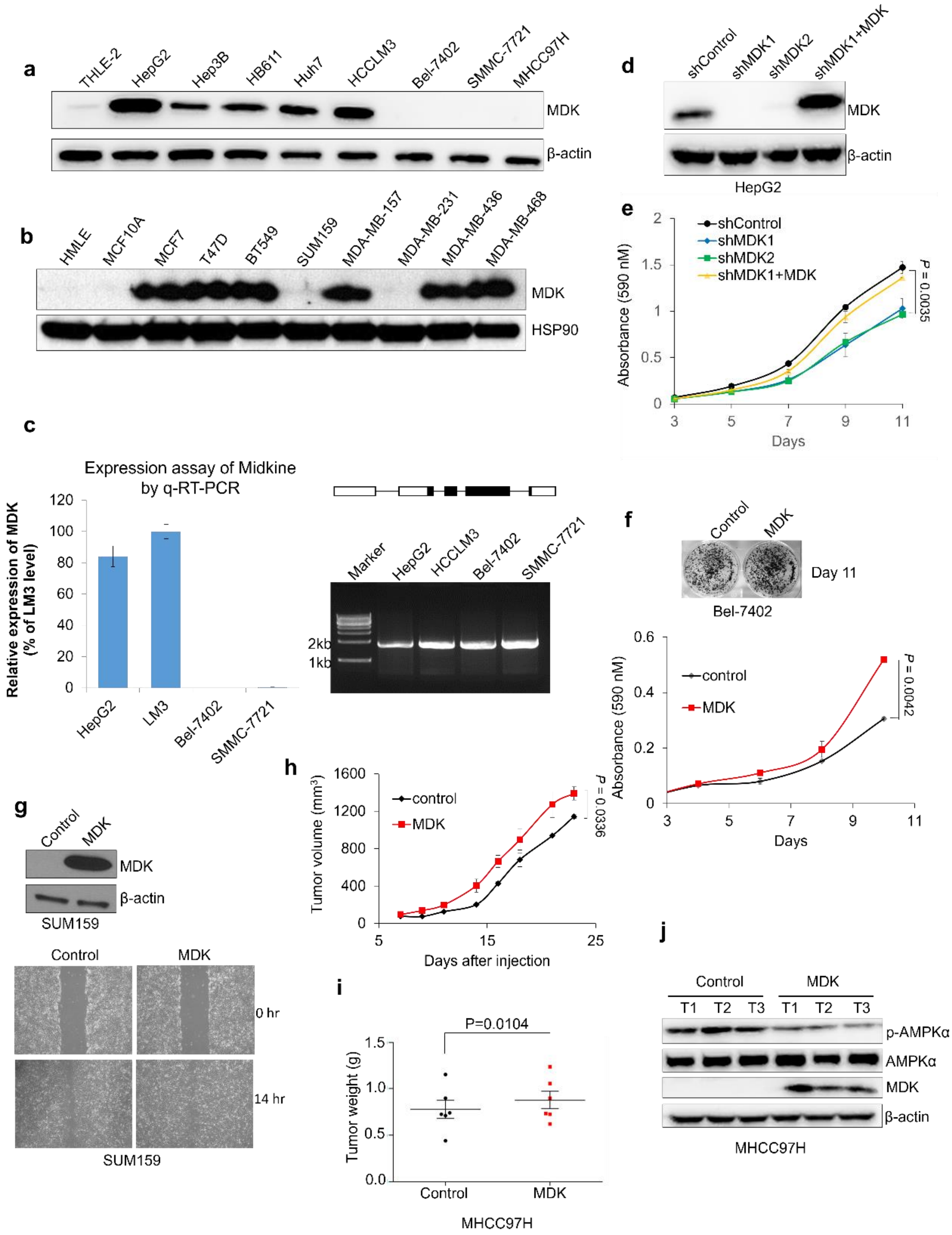

Supplementary Figure 5 | Midkine promotes cancer cell proliferation, invasion and tumorigenesis

a and b. Western blotting of MDK and β-actin in the THLE2 and HCC cell lines (A) and HMLE and breast cancer cell lines (B).

c. qRT-PCR of *MDK* expression in HerpG2, HCCLM3, Bel-7402 and SMMC-7721 cells (left panel). Structure of the MDK genomic DNA region (white boxes indicate

UTR, black boxes indicate ORF and black line indicate intron), and agarose gel image of PCR amplified MDK genomic DNA in HerpG2, HCCLM3, Bel-7402 and SMMC-7721 cells (right panel).

d and e. Western blot analysis of MDK and β-actin in the HepG2 cells transduced with two independent MDK shRNAs and restored MDK in the MDK-knockdown cells (D), and cell growth curves of the HepG2 cells transduced with two independent MDK shRNAs and restored MDK in the MDK-knockdown cells (E).

f. Representative images and cell growth curve in Bel-7402 cells stably expressing MDK.

g. Western blot analysis of MDK and β-actin in the restored MDK-expressing SUM159 cells and representative images of the wound healing migration of the MDK-overexpressing SUM159 cells. Bar=2 mm.

h and i. Tumor growth curve (h) and weight (i) after mice were subcutaneously injected with MHCC97H cells transduced the MDK and the control cells.

j. Western blotting of p-AMPKα, AMPKα, MDK, and β-actin in tumors derived from the MHCC97H cells overexpressing MDK.

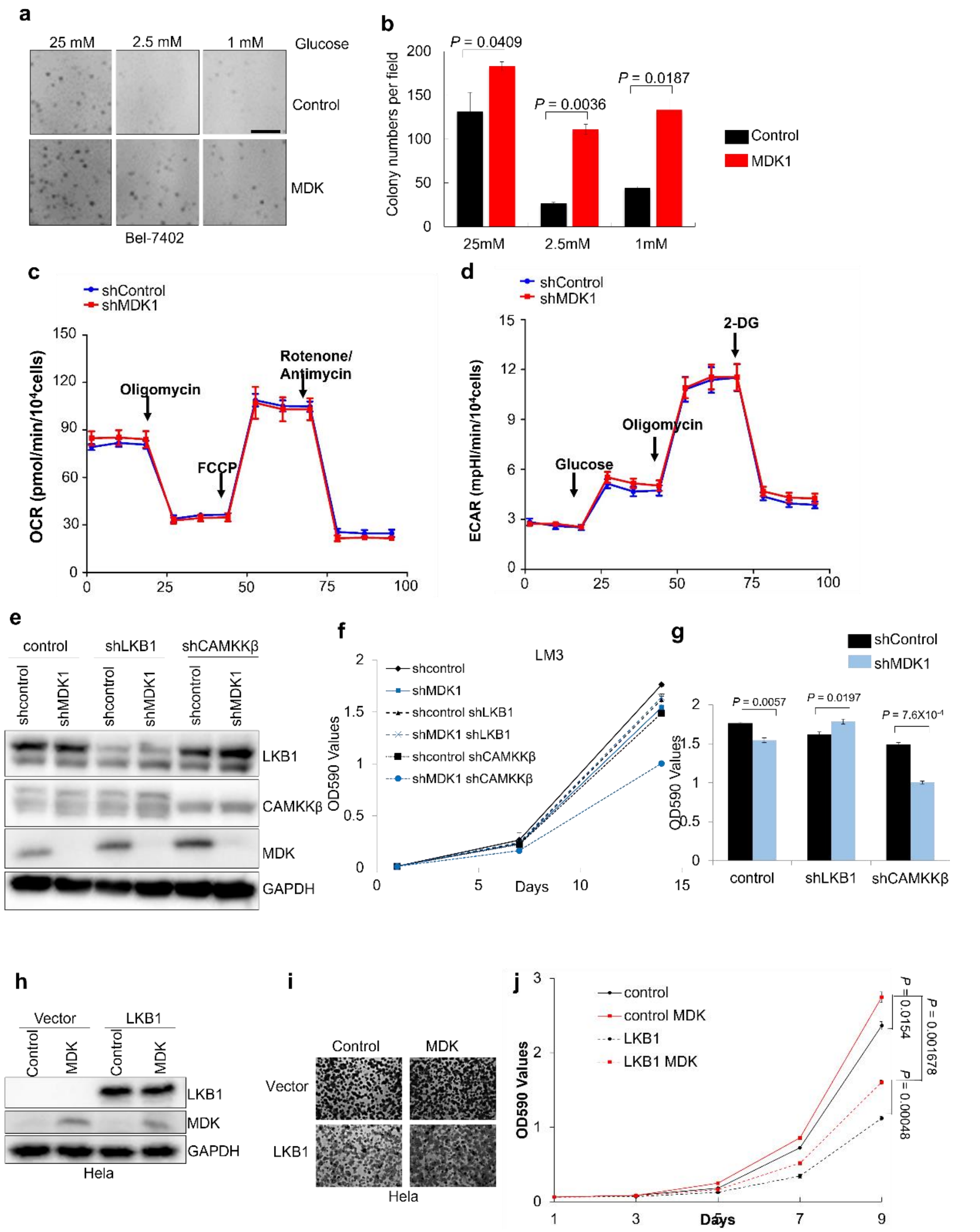

Supplementary Figure 6 | Midkine promotes cancer progression by negatively regulating AMPK signaling

a and b. Colony forming assay of the Bel-7402 cells transduced with MDK and the control cells treated with different concentrations of glucose. Images (a) and quantification (b) of colony formation. *n*=3 wells per group. Scale bar, 200 µm.

c. Cellular respiration measured as the basal and maximal oxygen consumption rates

(OCRs) in HCCLM3 cells transduced with MDK shRNA or control shRNA during sequential treatment with oligomycin (O), carbonyl cyanide p-trifluoromethoxyphenylhydrazone (FCCP) (F), and antimycin/rotenone (A/R). Bar chart indicates basal and maximal OCRs after FCCP injection; *n* = 3 wells per group.

d. ECARs were measured during sequential injection of oligomycin (O) and 2-deoxyglucose (2-DG) in the HCCLM3 cells transduced with MDK shRNA or control shRNA. Bar chart shows basal ECAR; *n* = 3 wells per group.

e. Western blotting of MDK, LKB1 CAMKKβ and GAPDH from HCCLM3 cells transduced with MDK shRNA alone or in combination with LKB1 shRNA or CAMKKβ shRNA.

f and g. Cell growth curve (f) and relative cell proliferation (g) of seeded HCCLM3 cells transduced with MDK shRNA alone or in combination with LKB1 shRNA or CAMKKβ shRNA.

h-j. LKB1 and MDK were stably expressed alone or simultaneously in Hela cells, and western blotting were performed to test the expression of MDK, LKB1 and GAPDH (h), the cells were seeded on 6-well plates to test cell proliferation by cell growth curve (i-j).

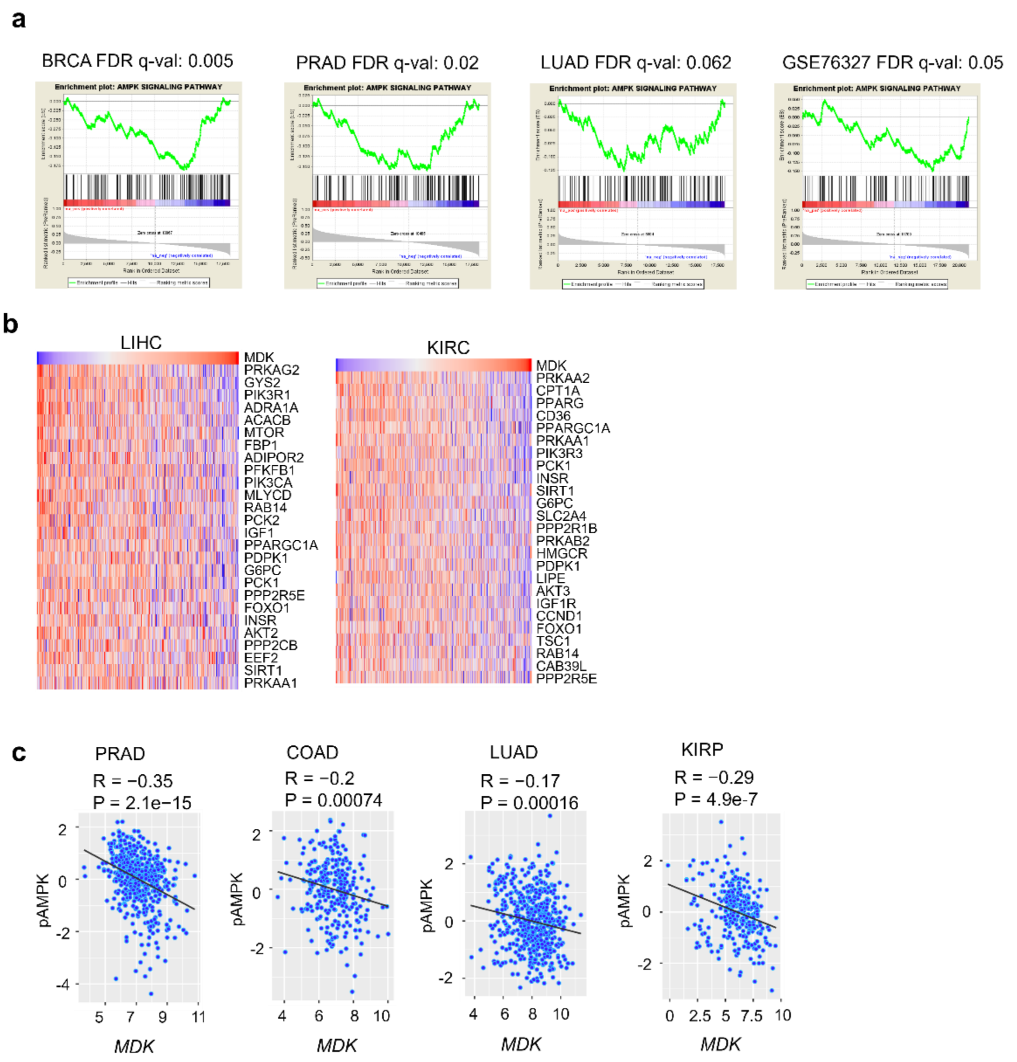

Supplementary Figure 7 | Midkine promotes cancer progression by negatively regulating AMPK signaling

a. GSEA results showing the negative correlations between MDK and the AMPK signaling pathway based on three TCGA cohorts (BRCA, PRAD and LUAD) and one GEO dataset (GSE76327).

b. Heat maps showing the top 25 MDK-correlated AMPK signaling pathway genes in the TCGA LIHC and KIRC RNA-seq datasets.

c. Scatter plots showing the inverse correlation of MDK with AMPK activity in the TCGA LUAD, PRAD, COAD and KIRP datasets (LUAD n=515, PRAD n=497, COAD n=286, KIRP n=290). Statistical significance was determined by the Pearson correlation test. R: Pearson correlation coefficient.

Supplementary Table 1

Characteristics	N	MDK		$\chi 2$	P
		+	-		
Gender					
Male	12	6	6	2.017	0.156
Female	62	44	18		
Ages（years）					
≤60	41	26	15	0.724	0.395
＞60	33	24	9		
HBV/HCV					
Negative	11	5	6	1.820	0.177
Positive	63	45	18		
AFP（ng/ml）					
≤20	29	22	7	1.497	0.221
＞20	45	28	17		
Liver cirrhosis					
No	22	12	10	2.423	0.120
Yes	52	38	14		
Tumor encapsulation					
None	18	14	4	1.132	0.287
Complete	56	36	20		
Tumor number					
Single	61	41	20	0.000	1.000
Mutiple	13	9	4		
Tumor Size					
≤5	41	24	17	3.422	0.064
＞5	33	26	7		
Differentiation					
Poor-moderate	37	29	8	3.947	0.047
Well	37	21	16		
TNM stage					
I+II	52	31	21	5.047	0.025
III+IV	22	19	3		
BCLC stage					
A	44	26	18	3.559	0.059
B+C	30	24	6		

REAGENT or RESOURCE	RESOURCE	IDENTIFIER
**Antibodies**		
Rabbit anti-p-AMPKα T172	Cell Signaling Technology	Cat# 2535; RRID: AB_331250
Rabbit anti-AMPKα	Cell Signaling Technology	Cat# 5831s; RRID: AB_10622186
Rabbit anti-ACC	Cell Signaling Technology	Cat# 3662S;RRID: AB_2219400
Rabbit anti-p-ACC S79	Cell Signaling Technology	Cat# 3661S; RRID: AB_330337
Rabbit anti-p-Akt S473	Cell Signaling Technology	Cat# 4060s; RRID: AB_2315049
Rabbit anti-Akt	Cell Signaling Technology	Cat# 4691s; RRID: AB_915783
Rabbit anti-p-S6K T389	Cell Signaling Technology	Cat# 9234s; RRID: AB_2269803
Rabbit anti-S6K	Cell Signaling Technology	Cat# 2708s; RRID: AB_390722
Rabbit anti-p-LKB1 S428	Cell Signaling Technology	Cat# 3482s; RRID: AB_2198321
Normal Rabbit IgG	Cell Signaling Technology	Cat# 2729s; RRID: AB_1031062
Rabbit anti-STRADα	Abcam	Cat# ab192879;
Rabbit anti-Mo25a	Abcam	Cat# ab51132; RRID: AB_868561
Mouse anti-LKB1	Abcam	Cat# ab15095; RRID: AB_301641
Normal mouse IgG	Santa Cruz Biotechnology	Cat# sc-2025; RRID: AB_737182
Mouse-anti-CAMKKβ	Santa Cruz Biotechnology	Cat# sc-sc271674; RRID: AB_10708844
Mouse anti-midkine	Santa Cruz Biotechnology	Cat# SC-46701; RRID: AB_627949
Mouse anti-FLAG Tag	Sigma	Cat# F1804; RRID: AB_262044
Mouse anti-GAPDH	Proteintech	Cat# 60004-1-Ig; RRID: AB_2107436
Rabbit anti-LKB1	Proteintech	Cat# 10746-1-AP; RRID: AB_2271311
Mouse anti-MYC Tag	Proteintech	Cat# 60003-2-Ig; RRID: AB_2734122
Mouse anti-HA Tag	Proteintech	Cat# 66006-1-Ig; RRID: AB_2857911
Rabbit anti-STRADβ	Proteintech	Cat# 10688-1AP; RRID: AB_2197715
Rabbit anti-AMPKβ1	Proteintech	Cat# 10308-1-AP; RRID: AB_513239
Rabbit anti-AMPKγ1	Proteintech	Cat# 10290-1-AP; RRID: AB_2268781
Mouse anti-β-actin	Proteintech	Cat# 66009-1-Ig; RRID: AB_268793810746-
Rabbit anti-AMPKα1	Proteintech	Cat# 10929-2-AP; RRID:

		AB_2169568
Rabbit anti- AMPKα2	Proteintech	Cat# 18167-1-AP; RRID: AB_10695046
**Chemicals and Reagents**		
DMEM	Gibco	Cat# 11995065
DMEM no glucose and sodium pyruvate	Gibco	Cat# 11966-026
RPMI 1640	Gibco	Cat# A1049101
RPMI 1640 no glucose	Gibco	Cat# 11879020
Trypsin	Yeasen	Cat# 40127ES60
Penicillin-Streptomycin	Yeasen	Cat# 60162ES76
Opti-MEM	Thermo Fisher Scientific	Cat# 31985070
FBS	Gibco	Cat# 10099-141
Matrigel Matrix	BioCoat	Cat# 356234
Heparin sodium	Selleck	Cat# S1346
Protein A/G Magnetic Beads	Bimake	Cat# B23201
S-Protein agarose	Millipore, USA	Cat# 69704
HpaI	Thermo Fisher Scientific	Cat# FD1034
EcoRI	Thermo Fisher Scientific	Cat# FD0274
NotI	Thermo Fisher Scientific	Cat# FD0596
NheI	Thermo Fisher Scientific	Cat# FD0974
KpnI	Thermo Fisher Scientific	Cat# FD0524
XhoI	Thermo Fisher Scientific	Cat# FD0694
PstI	Thermo Fisher Scientific	Cat# FD0614
T4 DNA ligase	Thermo Fisher Scientific	Cat# EL0011
BP CLONASE II ENZYME MIX	Thermo Fisher Scientific	Cat# 11789020
LR CLONASE II ENZYME MIX	Thermo Fisher Scientific	Cat# 11791020
Blasticidin	InvivoGen	Cat# ant-bl-1
Puromycin	InvivoGen	Cat# ant-pr-1
BCA protein assay kit	TIANGEN BIOTECH	Cat# PA115
PEI	Polysciences	Cat# 24765
2-Deoxy-D-Glucose	Sigma	Cat# D8375
Protease inhibitor Cocktail	Bimake	Cat# 14002
Phosphatase inhibitor	Bimake	Cat#15002

Cocktail		
**Biological Samples**		
Human HCC Tissue Microarrays	Shanghai Outdo Biotech Company	LivH150CS03
**Deposited Data**		
HCC expression data	TCGA	www.cbioportal.org
**Organisms/Strains**		
BALB/C nude mice	Beijing Vital River Laboratory Animal Technology Co., Ltd (Beijing, China)	N/A
**Recombinant DNA**		
pLKO.1-bsh-shRNA		
AMPKα1-shRNA CCGGGTGACCTCAC TTGACTCTTCTCTCGA GAGAAGAGTCAAGTG AGGTCACTTTTTTG	Sigma-Aldrich	TRCN0000199831
LKB1-shRNA CCGGGATCCTCAAGA AGAAGAAGTTCTCGA GAACTTCTTCTTCTTG AGGATCTTTTT	Sigma-Aldrich	TRCN0000000409
CAMKKβ-shRNA CCGGGTGAAGACCATG ATACGTAAACTCGAGTT TACGTATCATGGTCTTCA CTTTTT	Sigma-Aldrich	TRCN0000002299
Midkine-shRNA1 TGATTAAAGCTAAC GAGCA	In house	plenti
Midkine-shRNA2 TTCTTAGTAATGGA ATTGT	In house	plenti
SFB-AMPKα1	This paper	N/A
pCDNA-Flag-AMPKβ1	This paper	N/A
pCDNA-Flag-AMPKγ1	This paper	N/A
SFB-LKB1	This paper	N/A

SFB-LKB1NK	This paper	N/A
SFB-LKB1KC	This paper	N/A
pCDNA-Flag-LKB1	This paper	N/A
pCDNA-Flag-LKB1KD	This paper	N/A
pCDNA-Flag-LKB1NK	This paper	N/A
pCDNA-Flag-LKB1KC	This paper	N/A
SFB-CAMKKβ	This paper	N/A
MDK-SFB	This paper	N/A
pCDNA-MDKHA	This paper	N/A
pLoc-MDKHA	This paper	N/A
pLoc-MDKMyc	This paper	N/A
**Cell lines**		
MHCC97H	CTCC, Shanghai, China	
HUH7	CTCC, Shanghai, China	SCSP-528
BEL-7402	CTCC, Shanghai, China	TCHu182/ SCSP-526
Hep3B	CTCC, Shanghai, China	TCHu 10
HepG2	CTCC, Shanghai, China	SCSP-5045
SMMC-7721	CTCC, Shanghai, China	SCSP-510
HEK293T	CTCC, Shanghai, China	TCHu 52
HCC-LM3		GNHu44
HB611		N/A
THLE-2	ATCC	N/A
A549	ATCC	N/A
**Software and Algorithms**		
Image J	N/A	https://imagej.nih.gov/ij/
Gene Set Enrichment Analysis (GSEA)	Broad Institute	http://software.broadinstitute.org/gsea/index.jsp
SPSS 21.0	IBM	N/A
GEPIA	N/A	http://gepia.cancer-pku.cn/
CCLE	N/A	https://portals.broadinstitute.org/ccle
METABOANALYST	N/A	https://www.metaboanalyst.ca/
R2	N/A	https://hgserver1.amc.nl/cgi-bin/r2/main.cgi
