# Supplementary figures and images for: Midkine noncanonically suppresses AMPK activation through disrupting the LKB1-STRAD-Mo25 complex
Source: Cell Death Dis. 2022 Apr 29;13(4):414. doi: 10.1038/s41419-022-04801-0 (PMC9054788; doi:10.1038/s41419-022-04801-0)

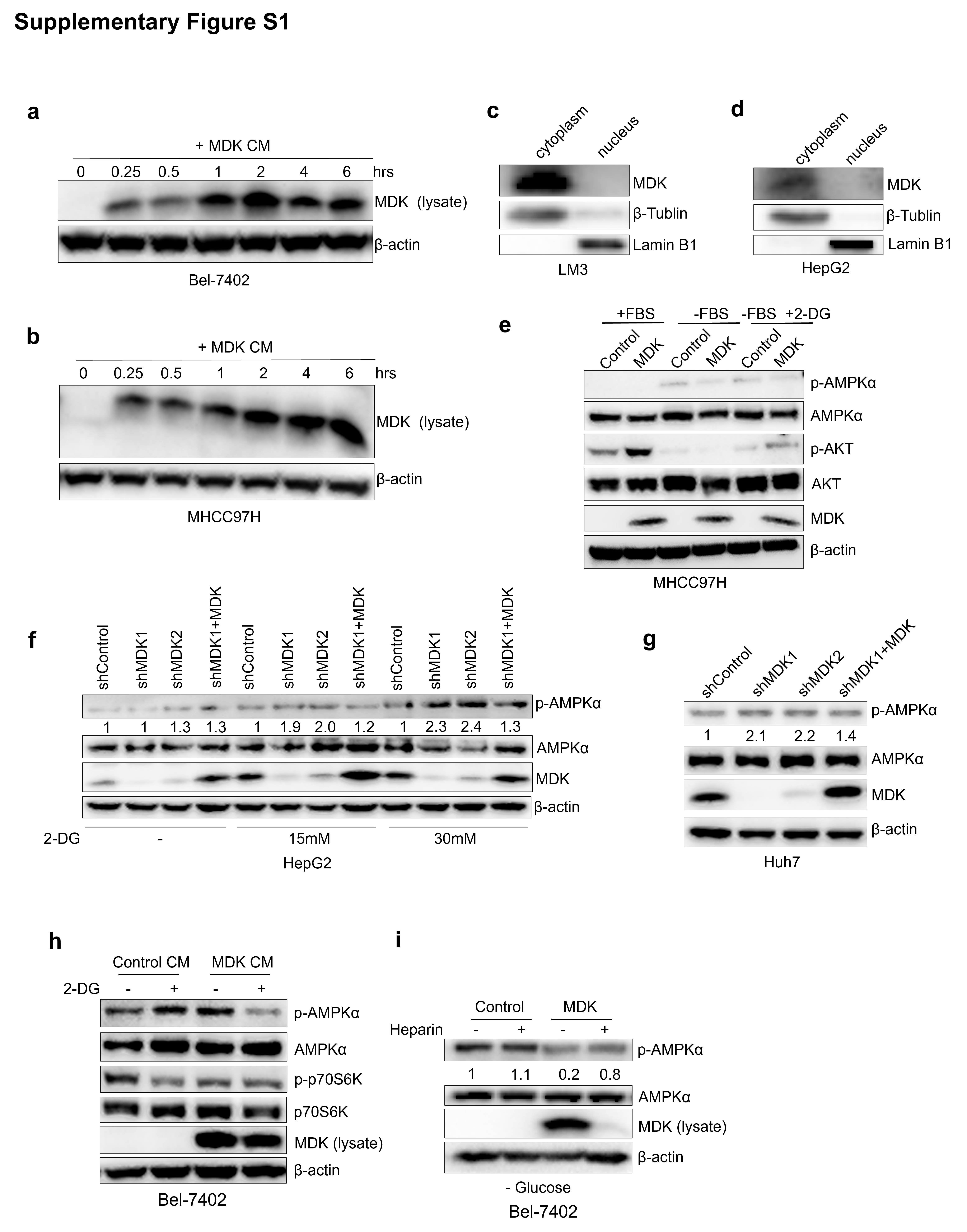

Supplement: Supplementary file 2 — Supplementary Figure S1 [file 41419_2022_4801_MOESM2_ESM.png]

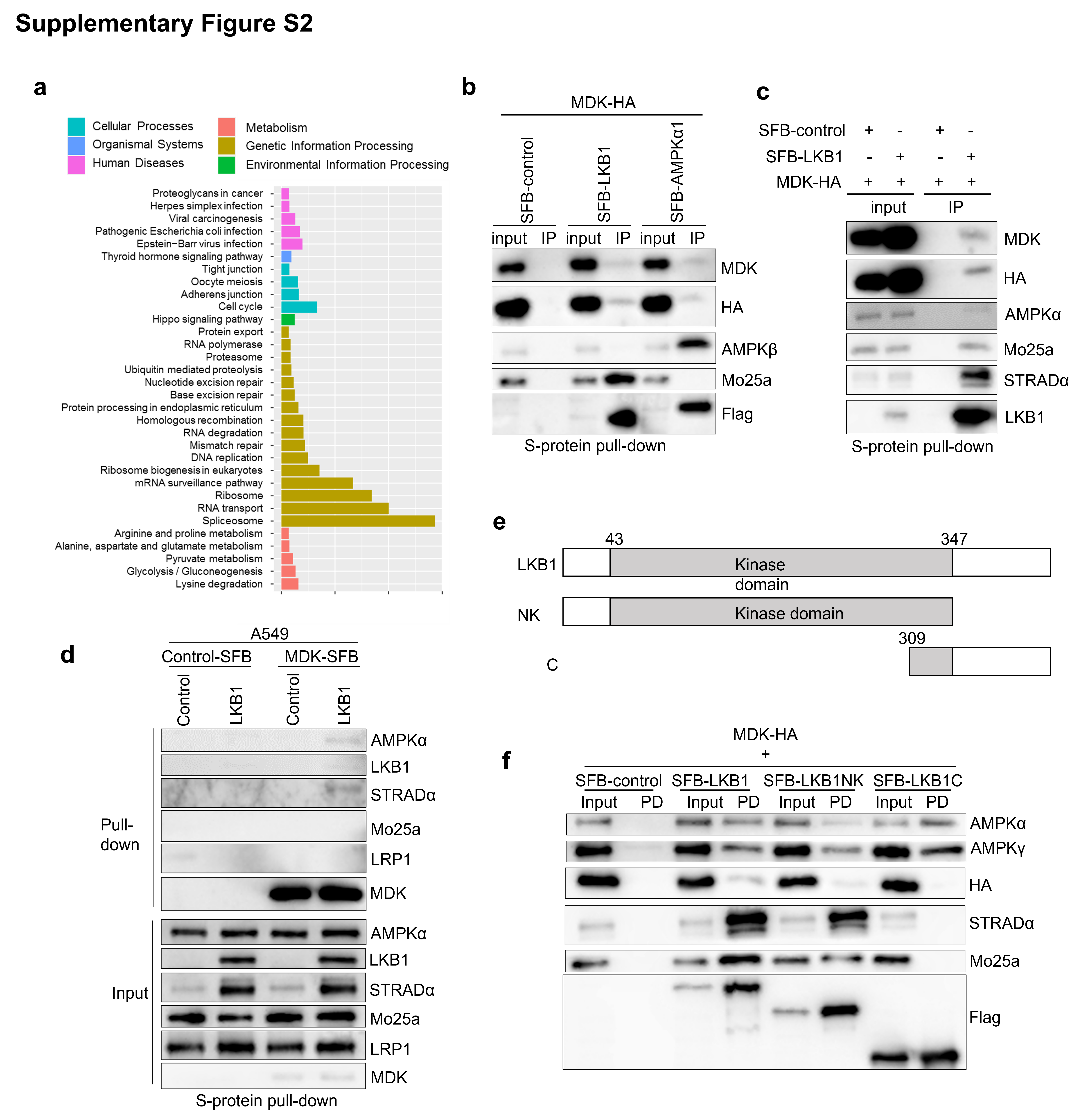

Supplement: Supplementary file 3 — Supplementary Figure S2 [file 41419_2022_4801_MOESM3_ESM.png]

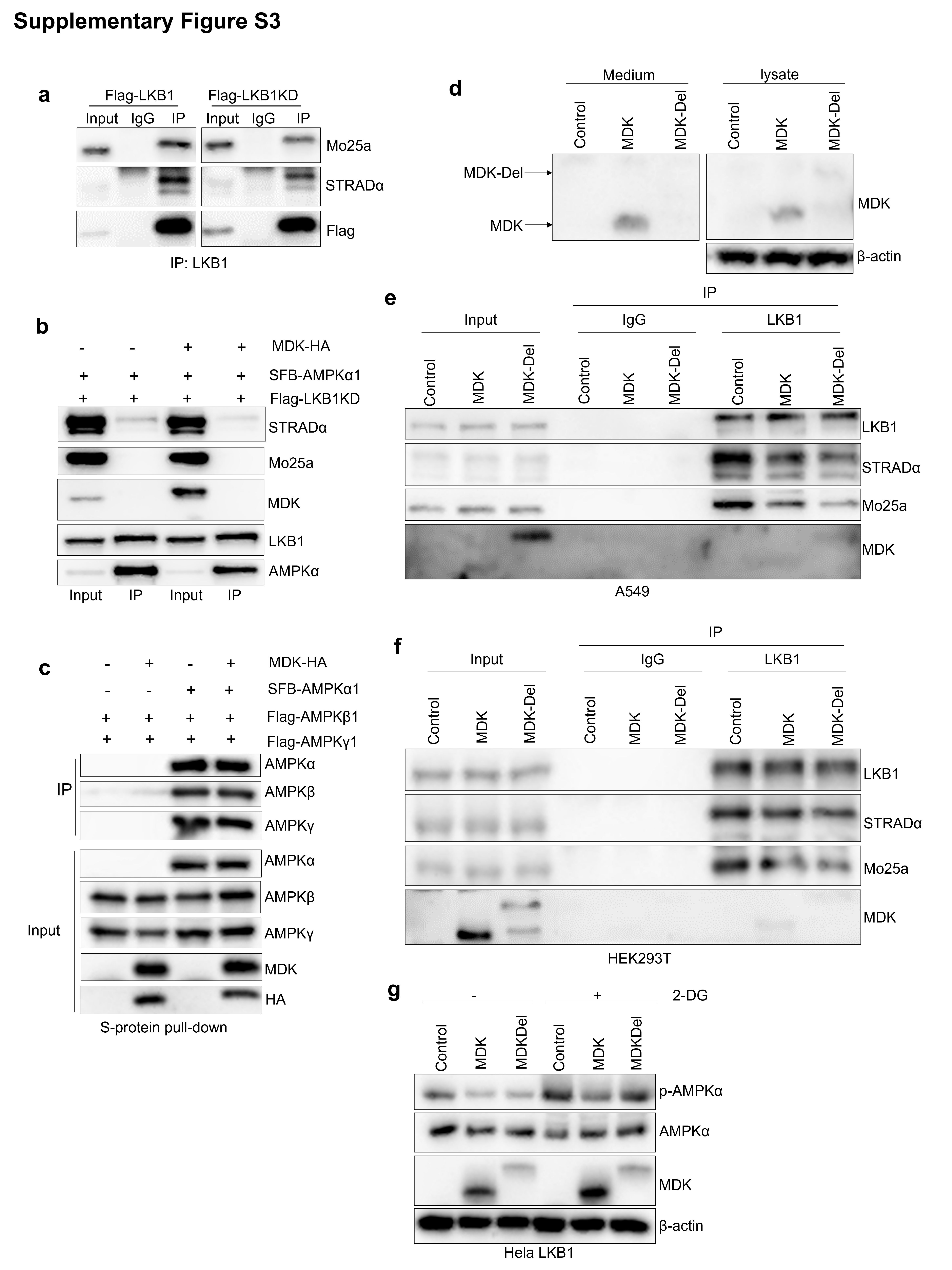

Supplement: Supplementary file 4 — Supplementary Figure S3 [file 41419_2022_4801_MOESM4_ESM.png]

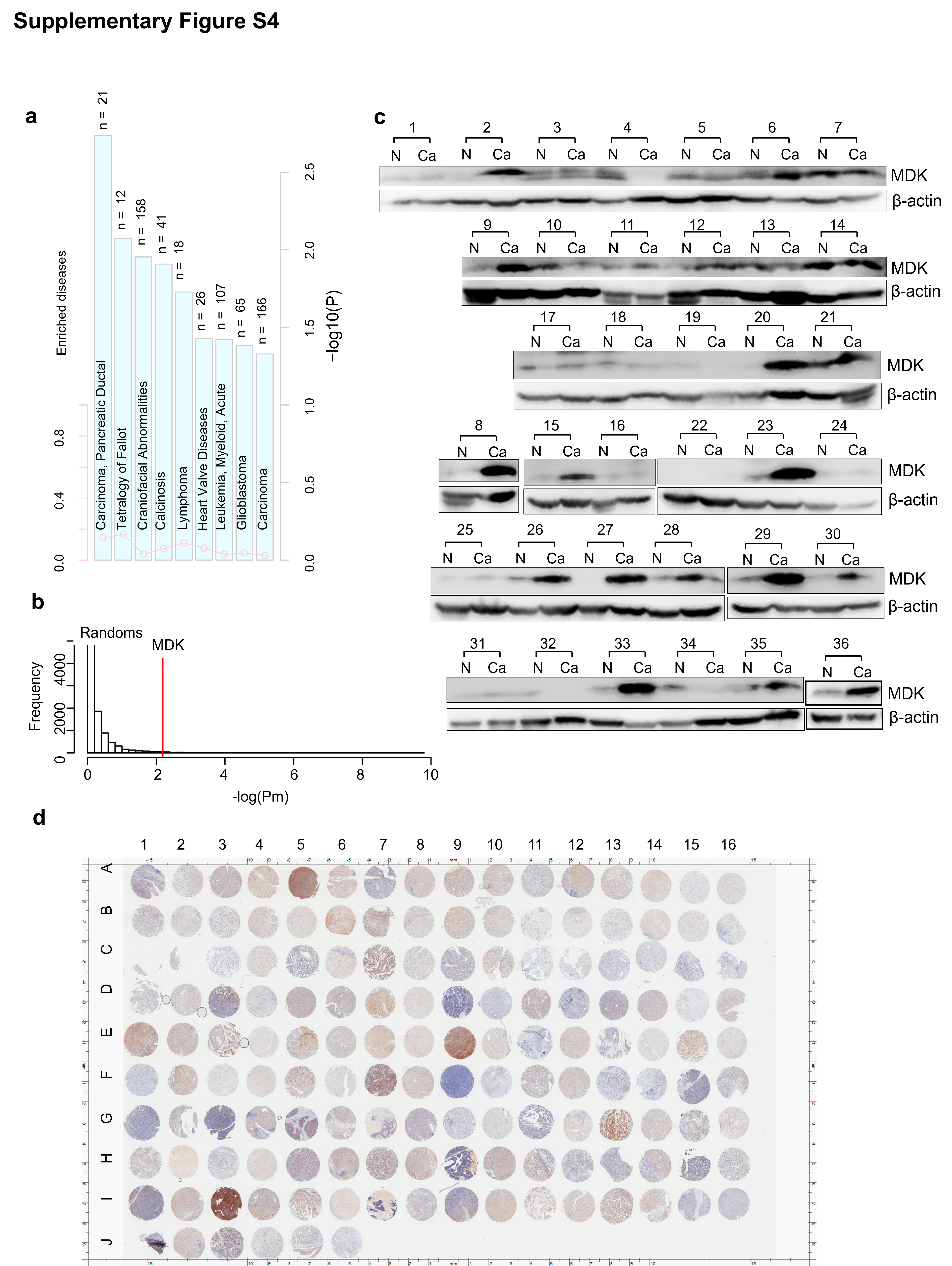

Supplement: Supplementary file 5 — Supplementary Figure S4 [file 41419_2022_4801_MOESM5_ESM.png]

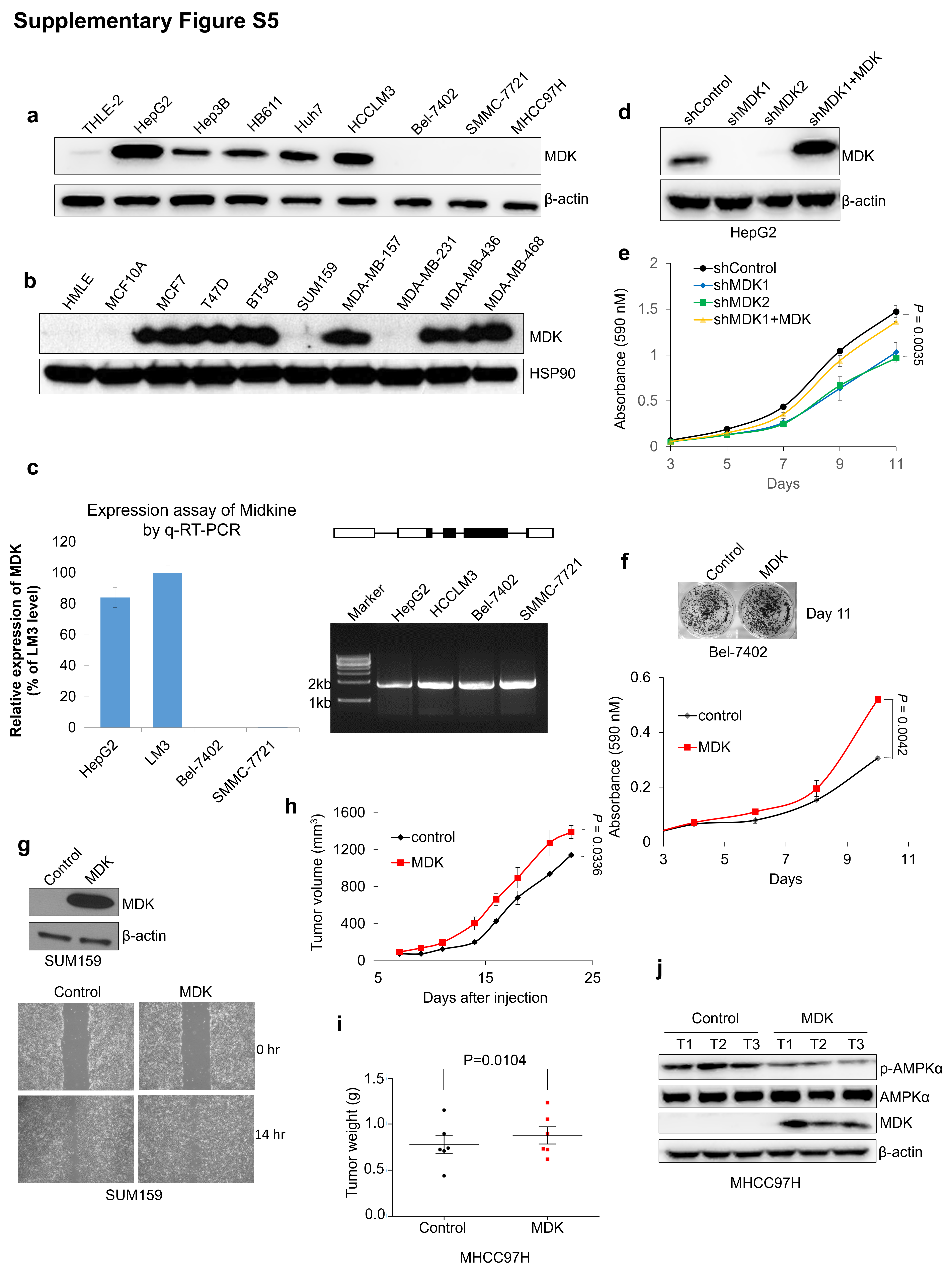

Supplement: Supplementary file 6 — Supplementary Figure S5 [file 41419_2022_4801_MOESM6_ESM.png]

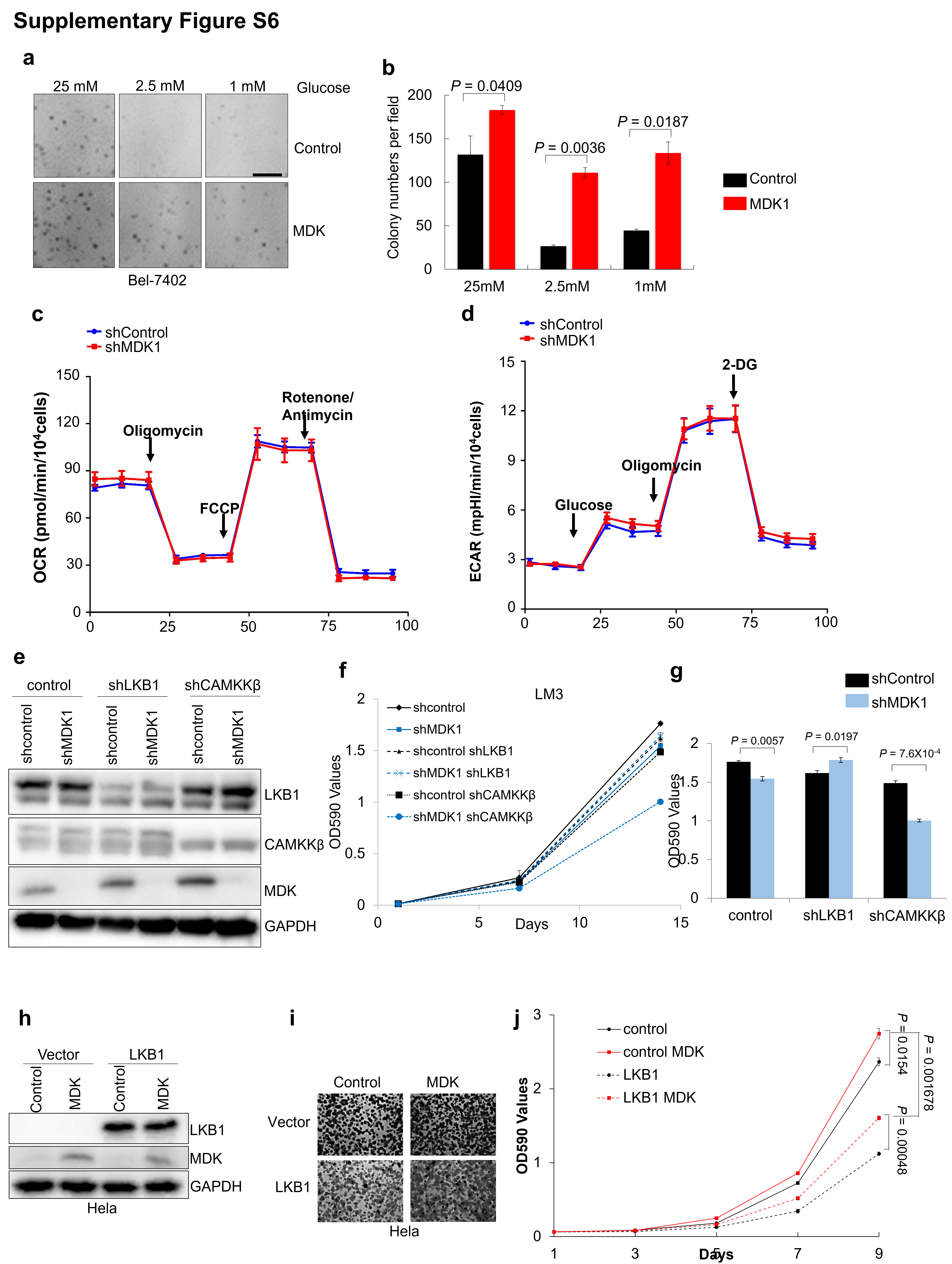

Supplement: Supplementary file 7 — Supplementary Figure S6 [file 41419_2022_4801_MOESM7_ESM.png]

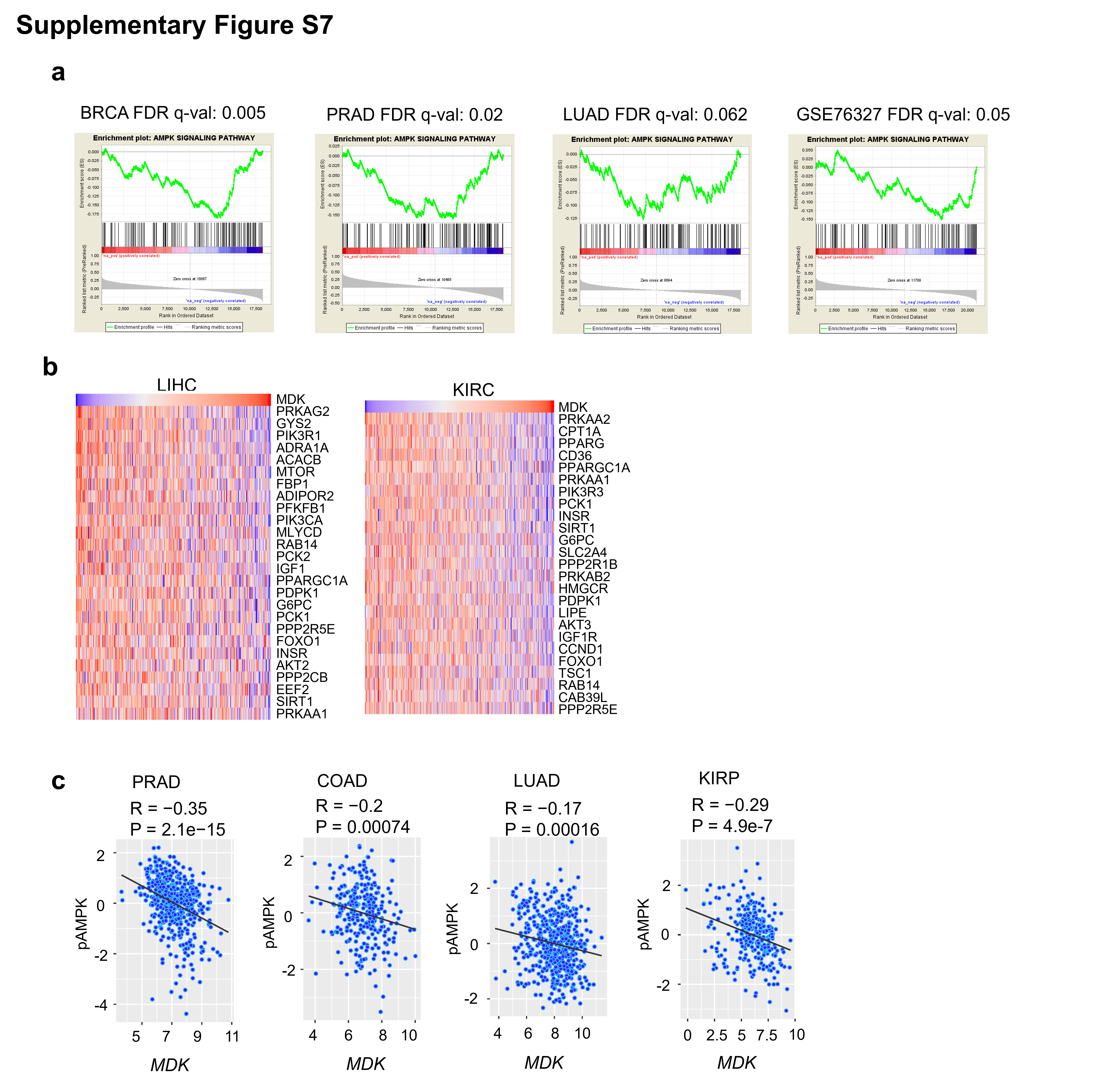

Supplement: Supplementary file 8 — Supplementary Figure S7 [file 41419_2022_4801_MOESM8_ESM.png]
