# Supplementary material for: Midkine noncanonically suppresses AMPK activation through disrupting the LKB1-STRAD-Mo25 complex
Source: Cell Death Dis. 2022 Apr 29;13(4):414. doi: 10.1038/s41419-022-04801-0 (PMC9054788; doi:10.1038/s41419-022-04801-0)

Fig.1A

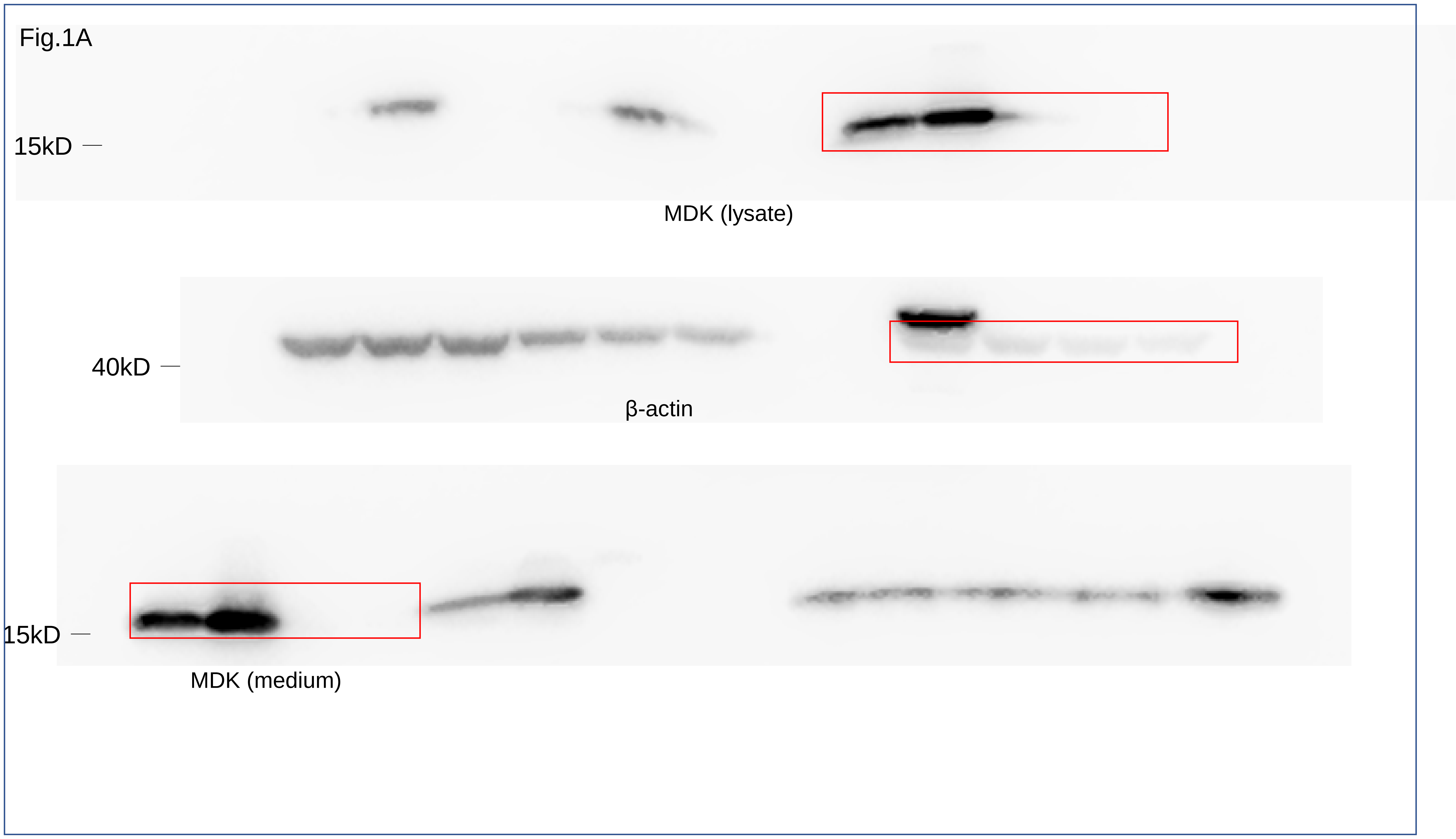
15kD
MDK (lysate)
40kD
β-actin
15kD
MDK (medium)

Fig.1B

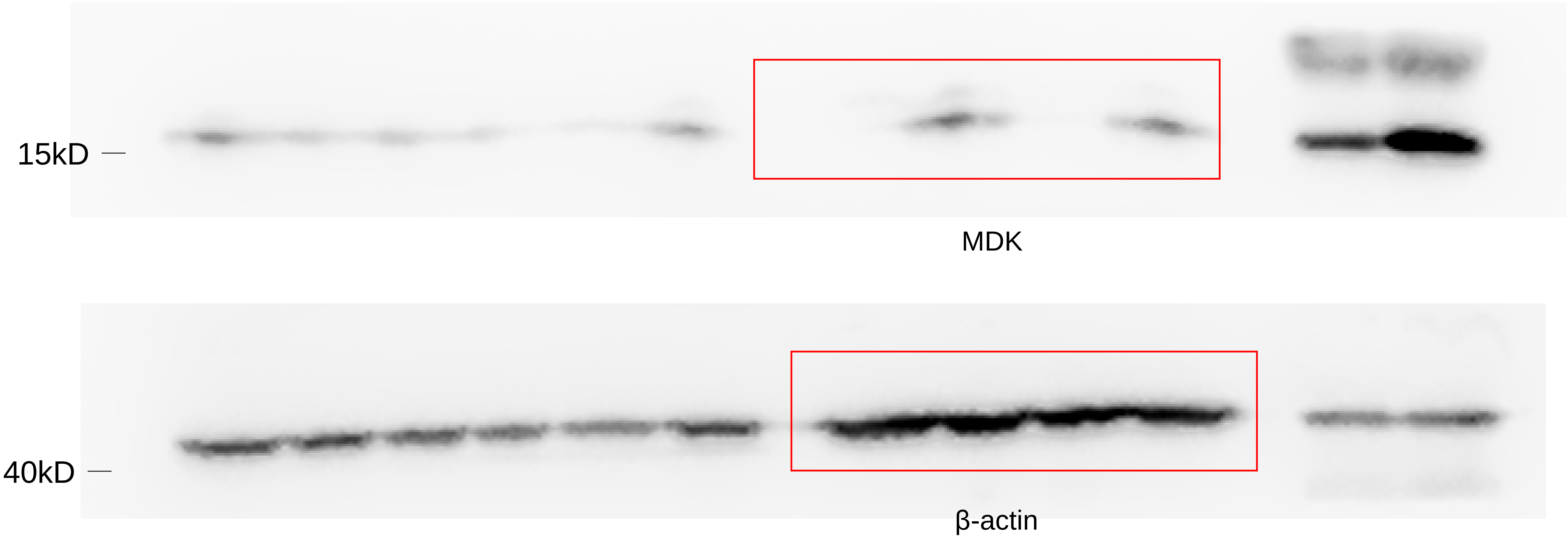
15kD
MDK
40kD
β-actin

Fig.1C

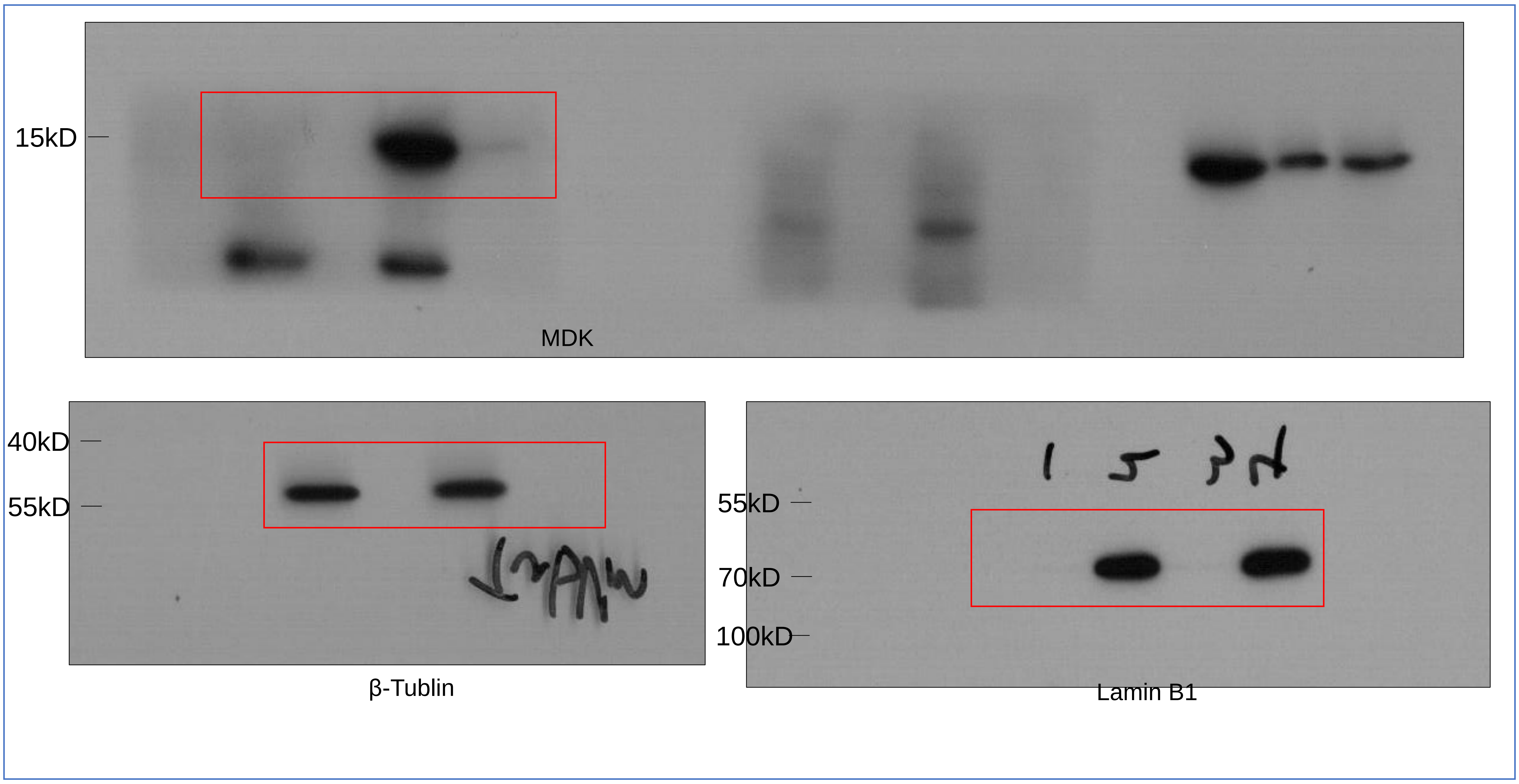
15kD
MDK
40kD
55kD
β-Tublin
55kD
70kD
100kD
Lamin B1

Fig.1E

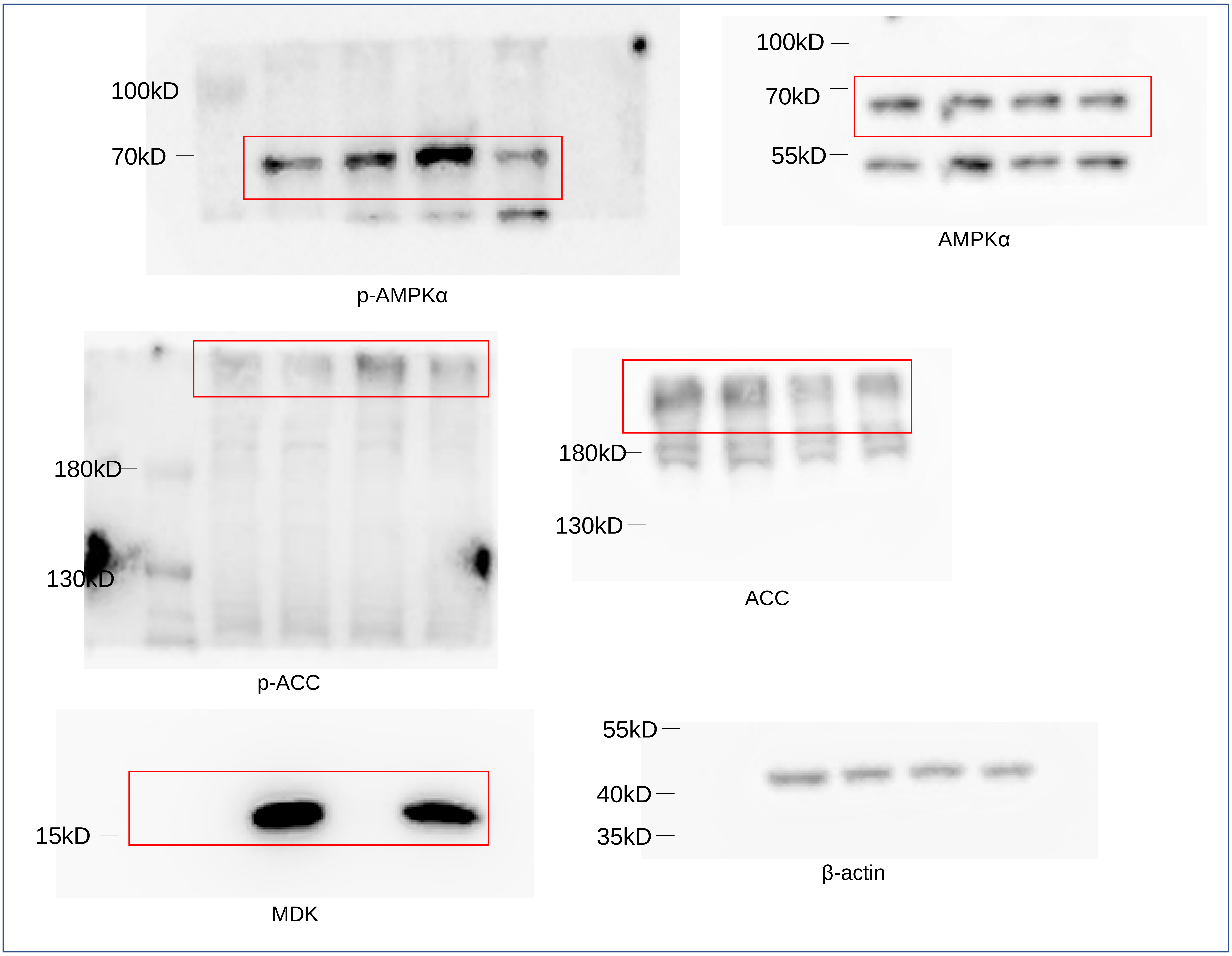
100kD
70kD
p-AMPKα
100kD
70kD
55kD
AMPKα
180kD
130kD
p-ACC
180kD
130kD
ACC
15kD
MDK
55kD
40kD
35kD
β-actin

Fig.1F

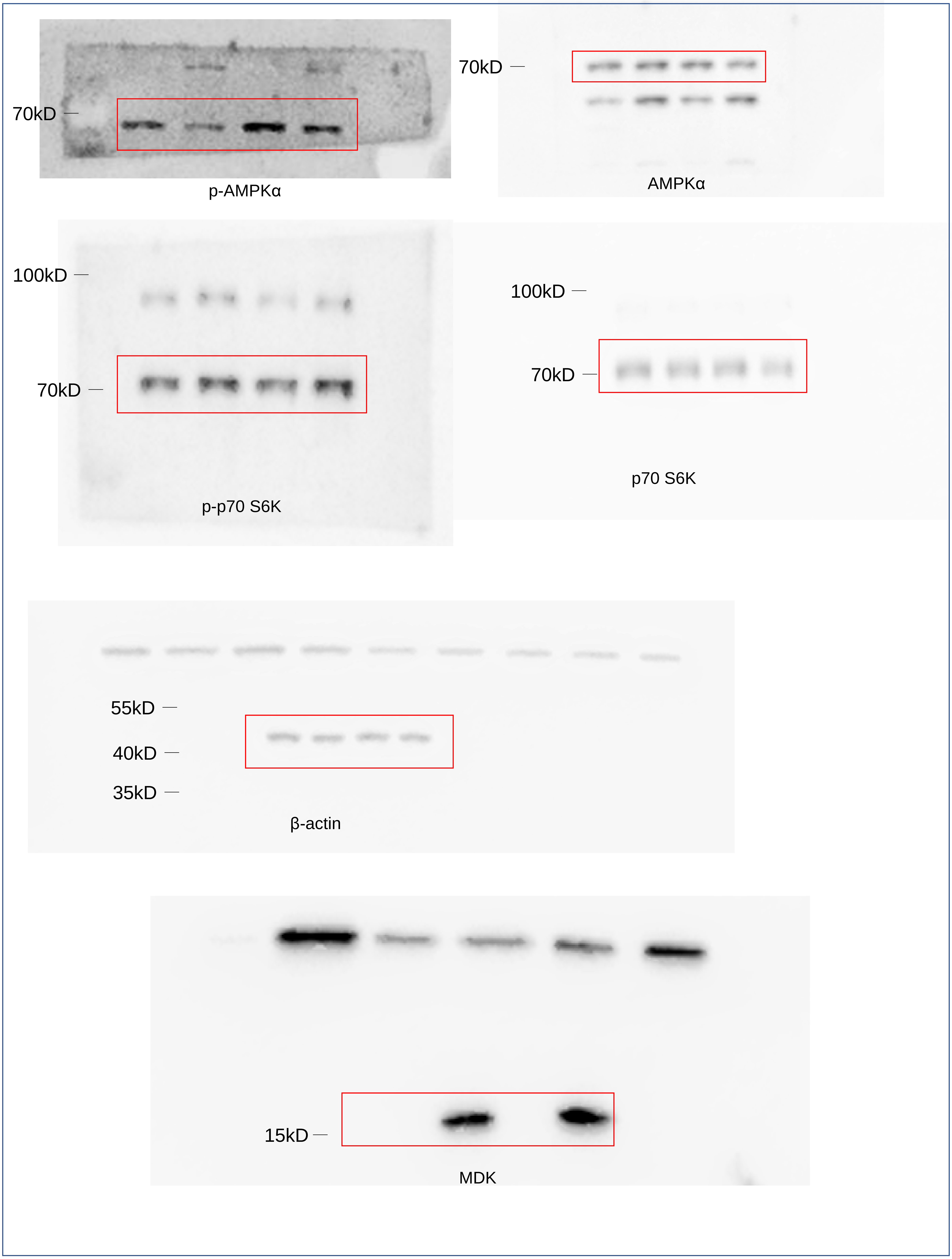
70kD
p-AMPKα
70kD
AMPKα
100kD
70kD
p-p70 S6K
100kD
70kD
p70 S6K
55kD
40kD
35kD
β-actin
15kD
MDK

Fig.1G

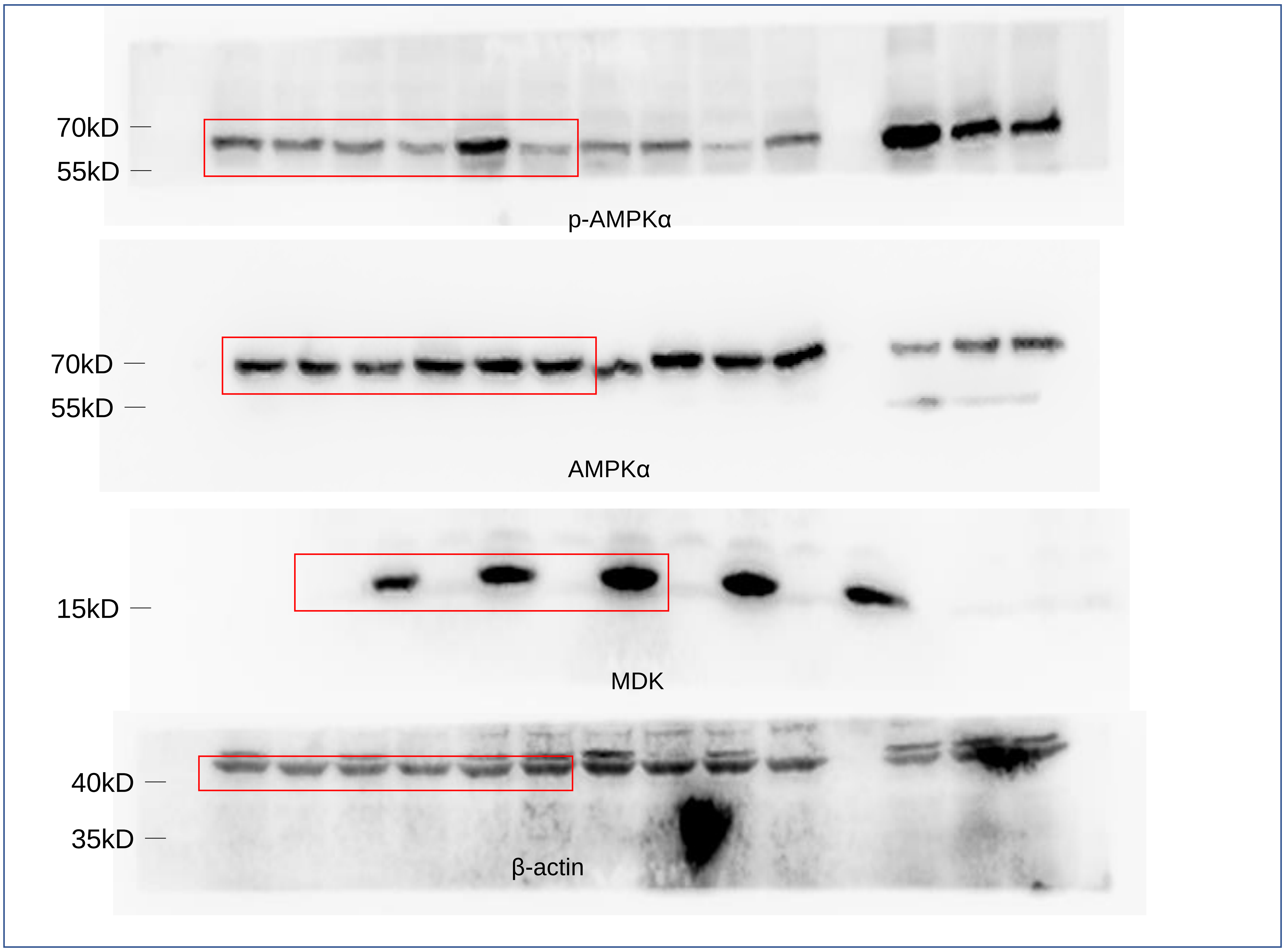
70kD
55kD
p-AMPKα
70kD
55kD
AMPKα
15kD
MDK
40kD
35kD
β-actin

Fig.1H

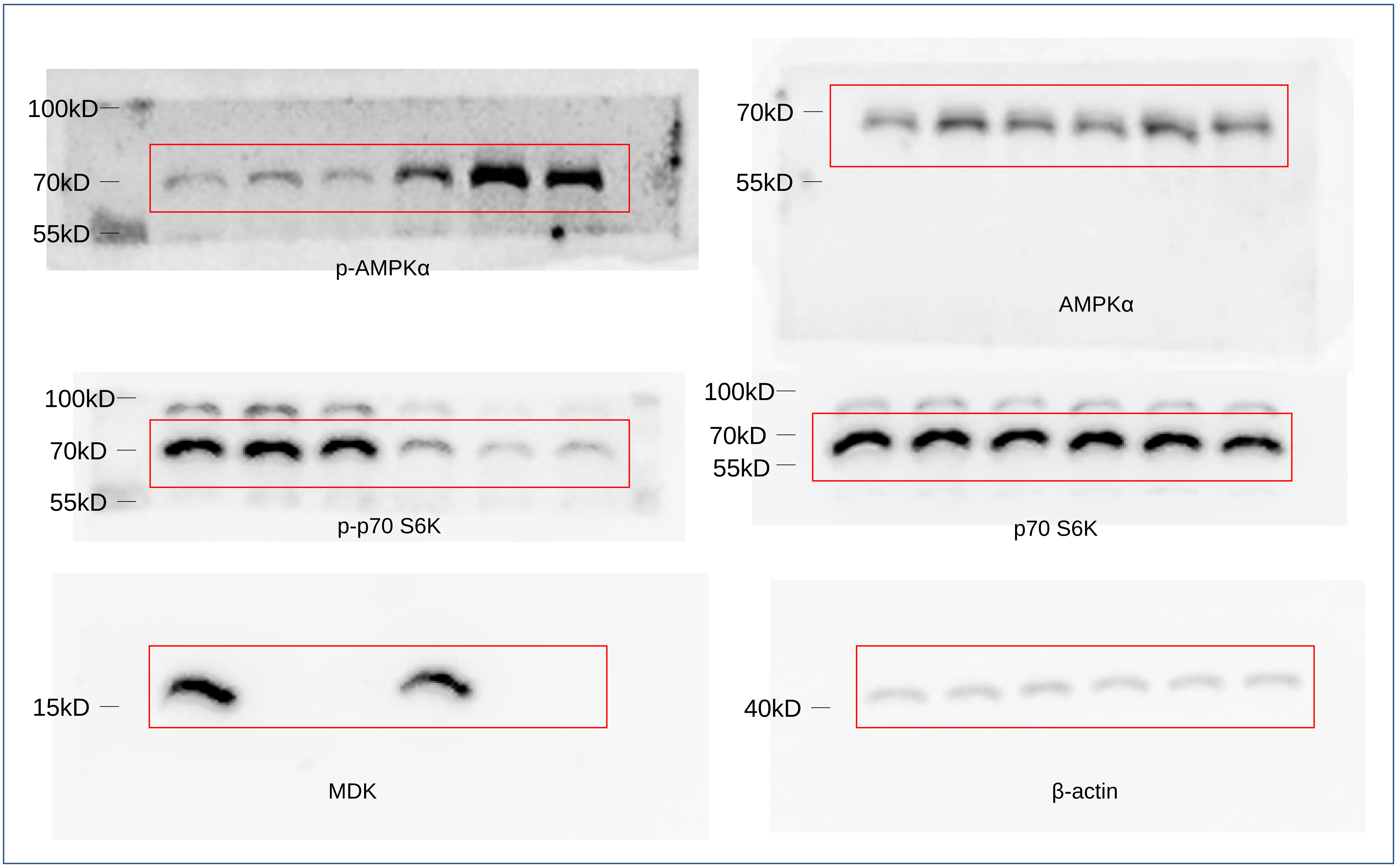
100kD
70kD
55kD
p-AMPKα
70kD
55kD
AMPKα
100kD
70kD
55kD
p-p70 S6K
100kD
70kD
55kD
p70 S6K
15kD
MDK
40kD
β-actin

Fig.1I

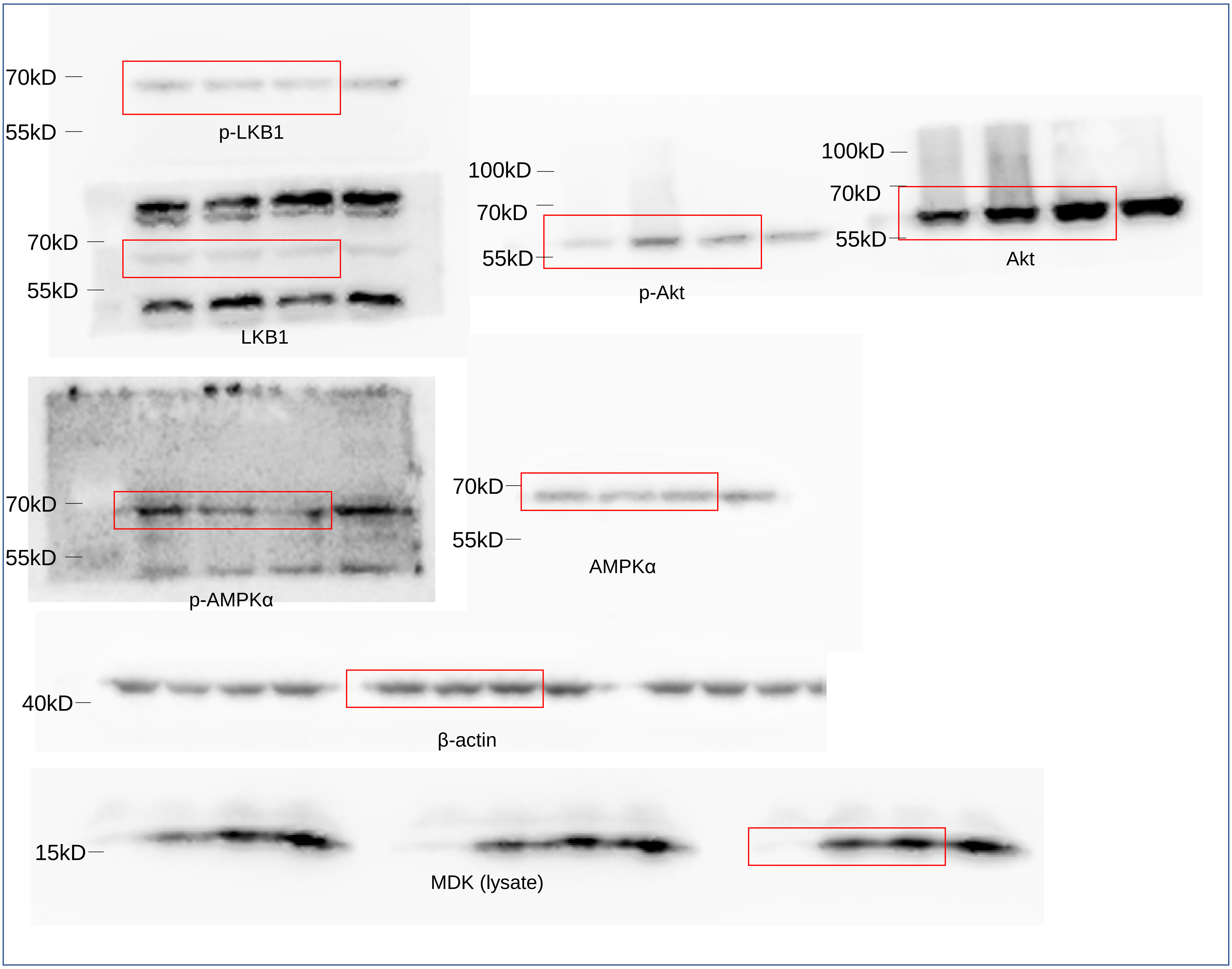
70kD
55kD
p-LKB1
70kD
55kD
LKB1
100kD
70kD
55kD
p-Akt
100kD
70kD
55kD
Akt
70kD
55kD
p-AMPKα
70kD
55kD
AMPKα
40kD
β-actin
15kD
MDK (lysate)

Fig.1J

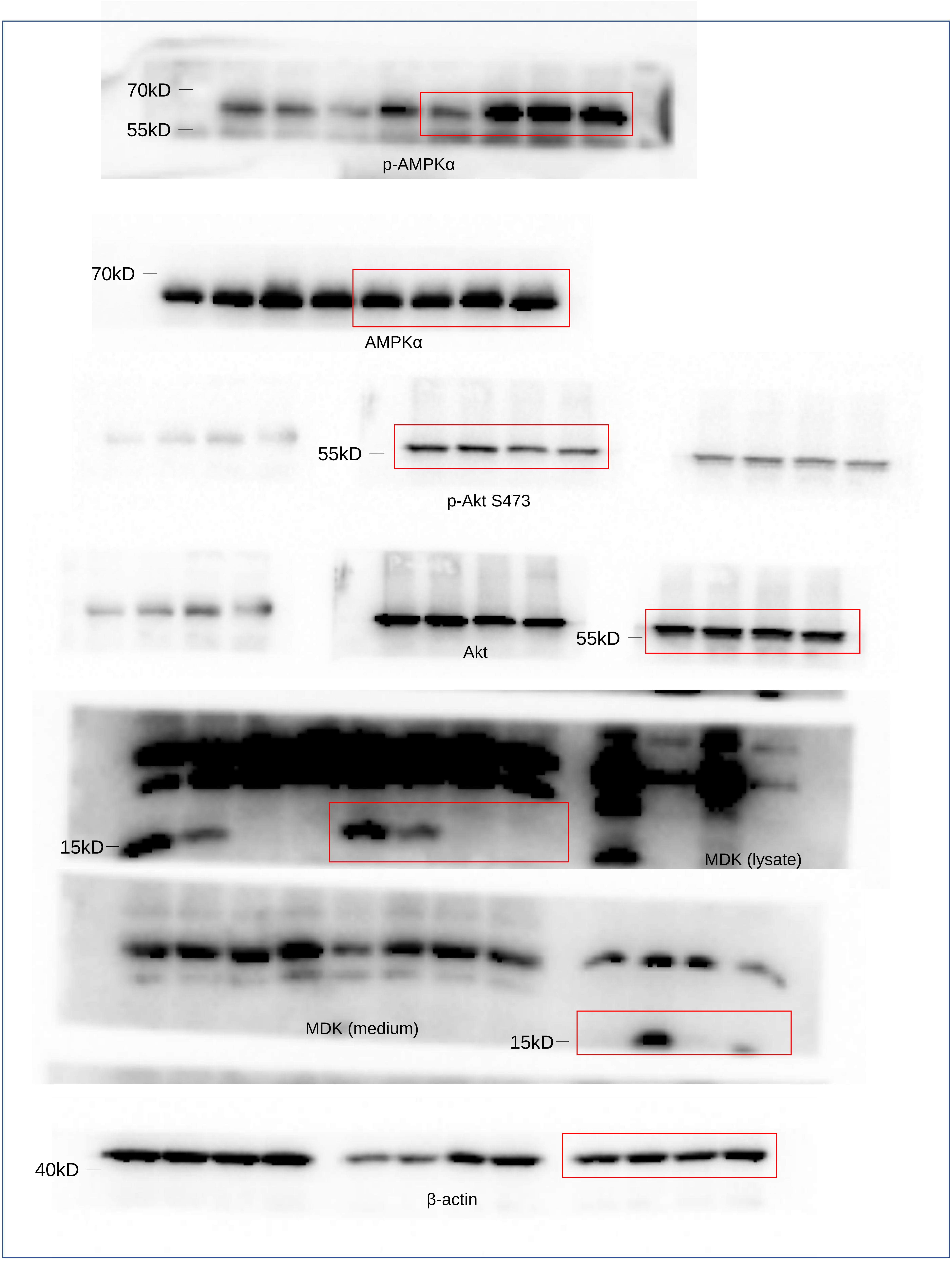

70kD
55kD
p-AMPKα
70kD
AMPKα
55kD
p-Akt S473
Akt
55kD
15kD
MDK (lysate)
MDK (medium)
15kD
40kD
β-actin

Fig.1K

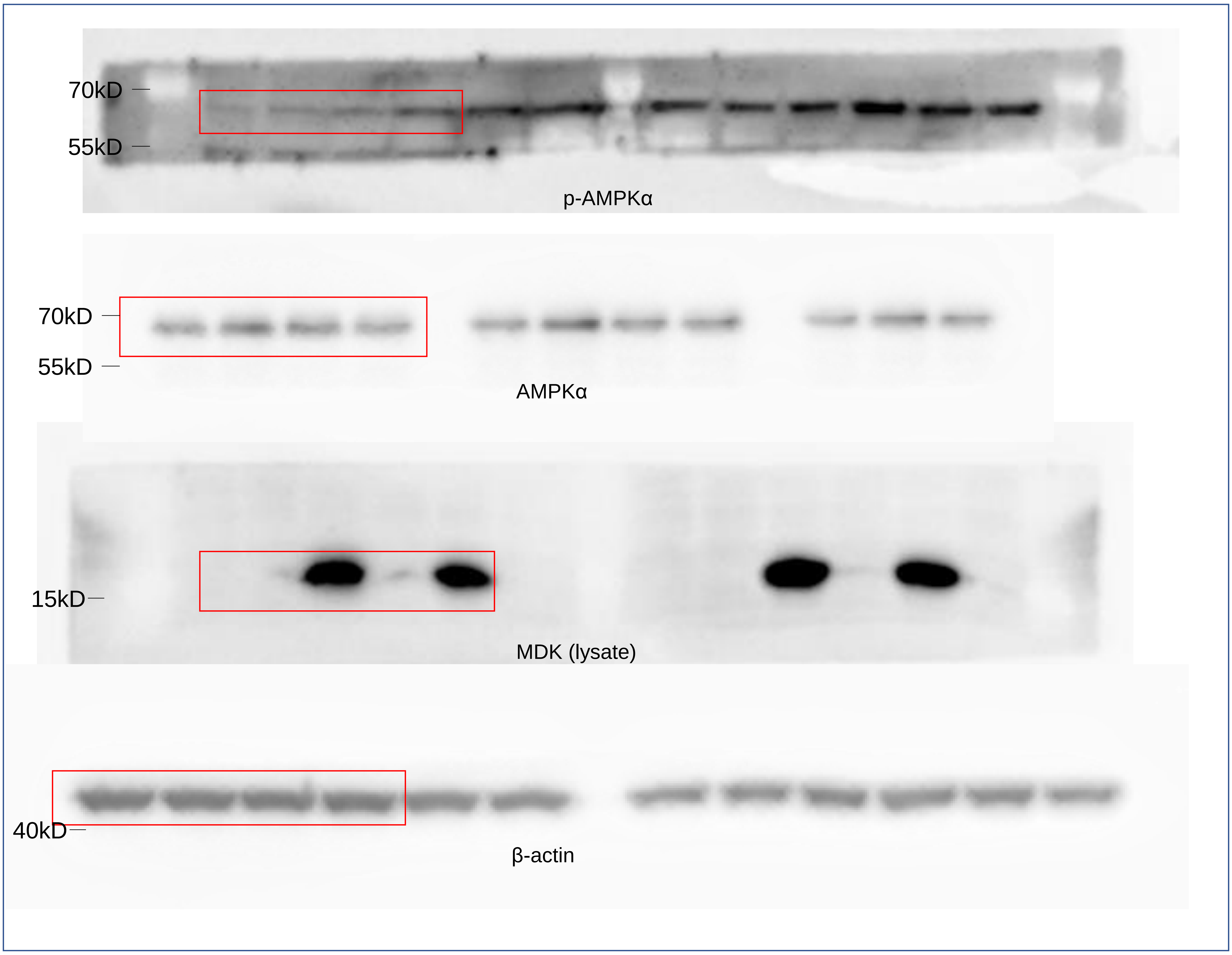
70kD
55kD
p-AMPKα
70kD
55kD
AMPKα
15kD
MDK (lysate)
40kD
β-actin

Fig.1L

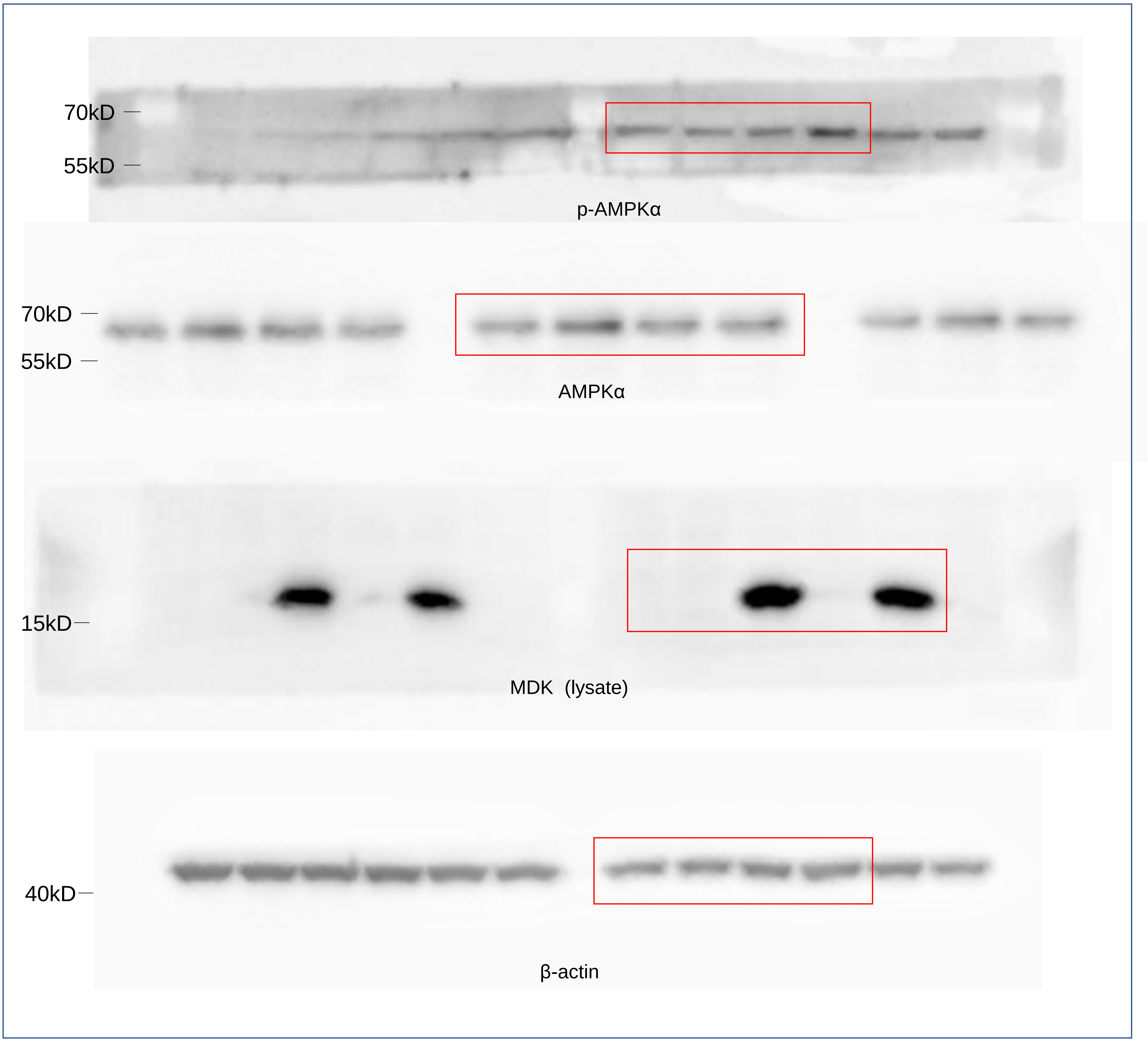
70kD
55kD
p-AMPKα
70kD
55kD
AMPKα
15kD
MDK (lysate)
40kD
β-actin

Fig.2B

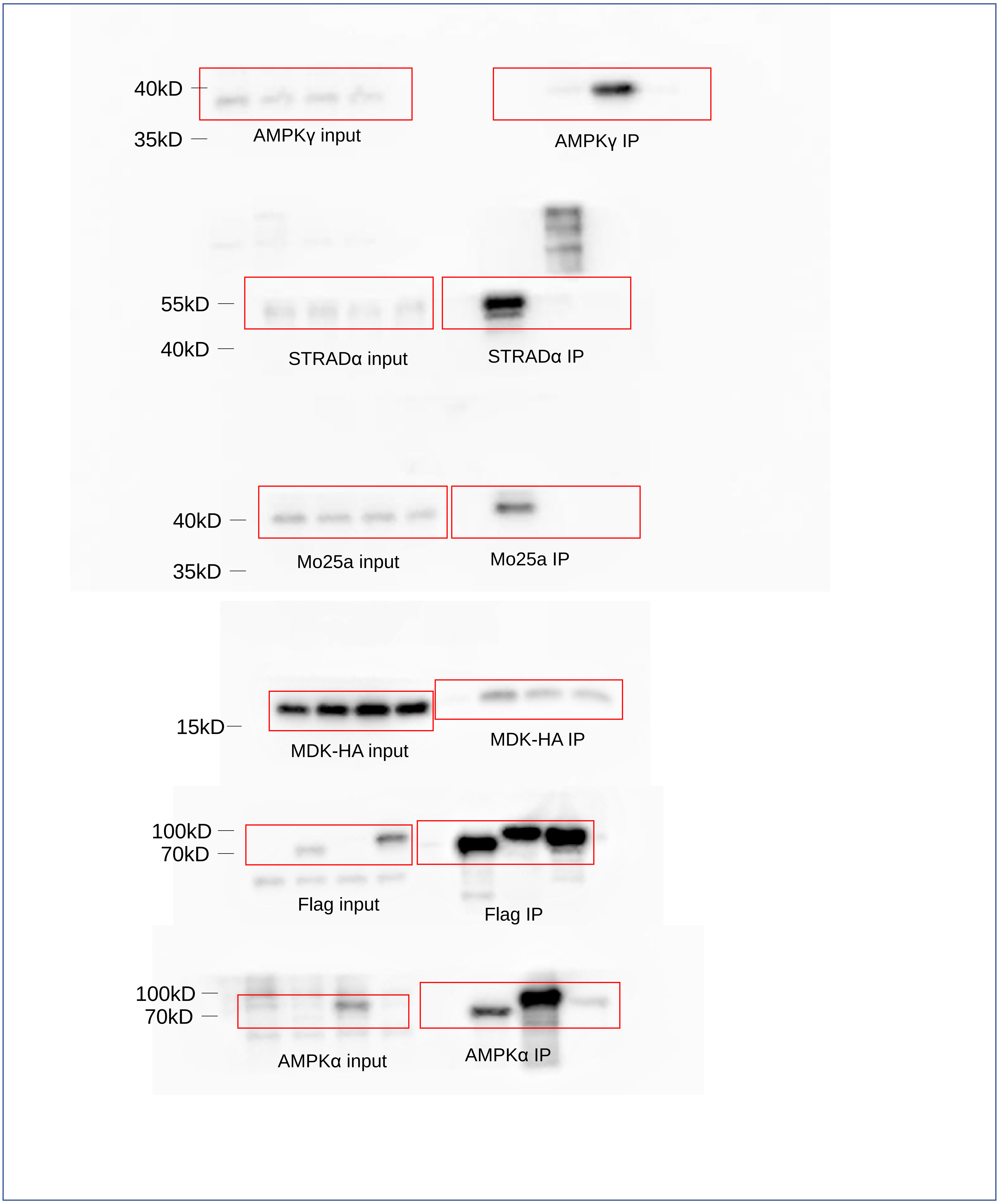
40kD
35kD
AMPKγ input
AMPKγ IP
55kD
40kD
STRADα input
STRADα IP
40kD
35kD
Mo25a input
Mo25a IP
15kD
MDK-HA input
MDK-HA IP
100kD
70kD
Flag input
Flag IP
100kD
70kD
AMPKα input
AMPKα IP

Fig.2C

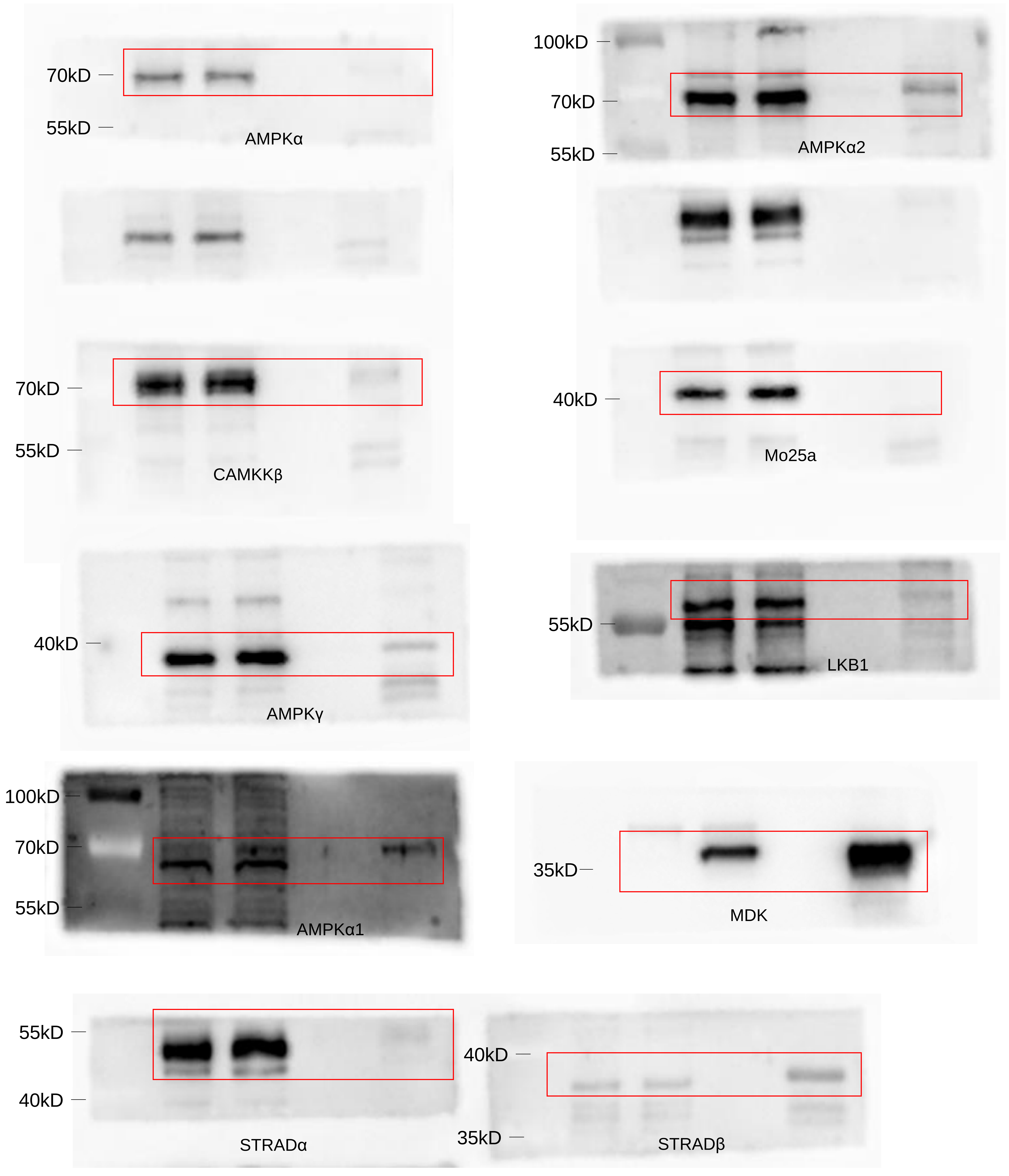
70kD
55kD
AMPKα
100kD
70kD
55kD
AMPKα2
70kD
55kD
CAMKKβ
40kD
Mo25a
40kD
AMPKγ
55kD
LKB1
100kD
70kD
55kD
AMPKα1
35kD
MDK
55kD
40kD
STRADα
40kD
35kD
STRADβ

Fig.2D-E

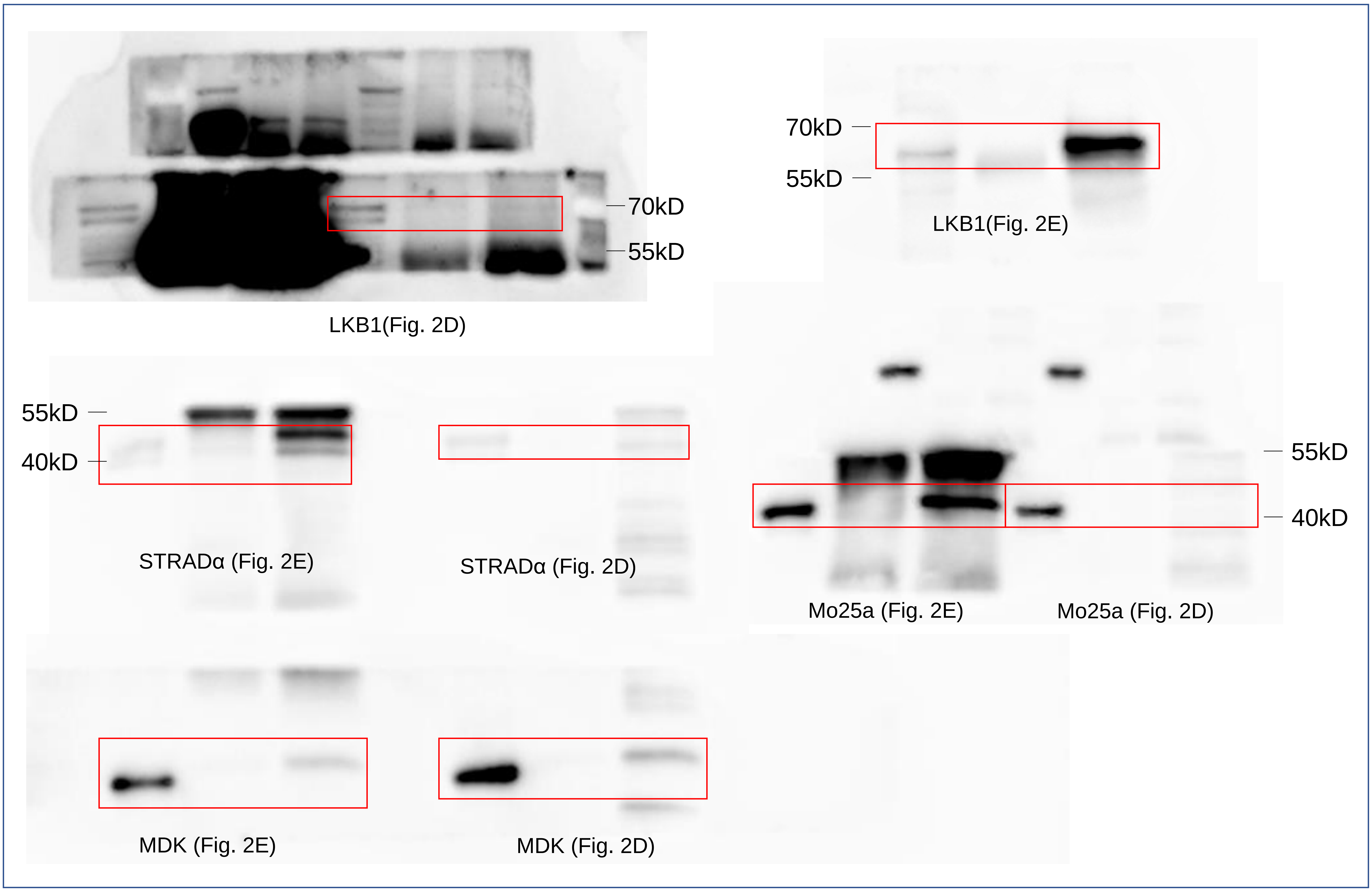


Fig.2F

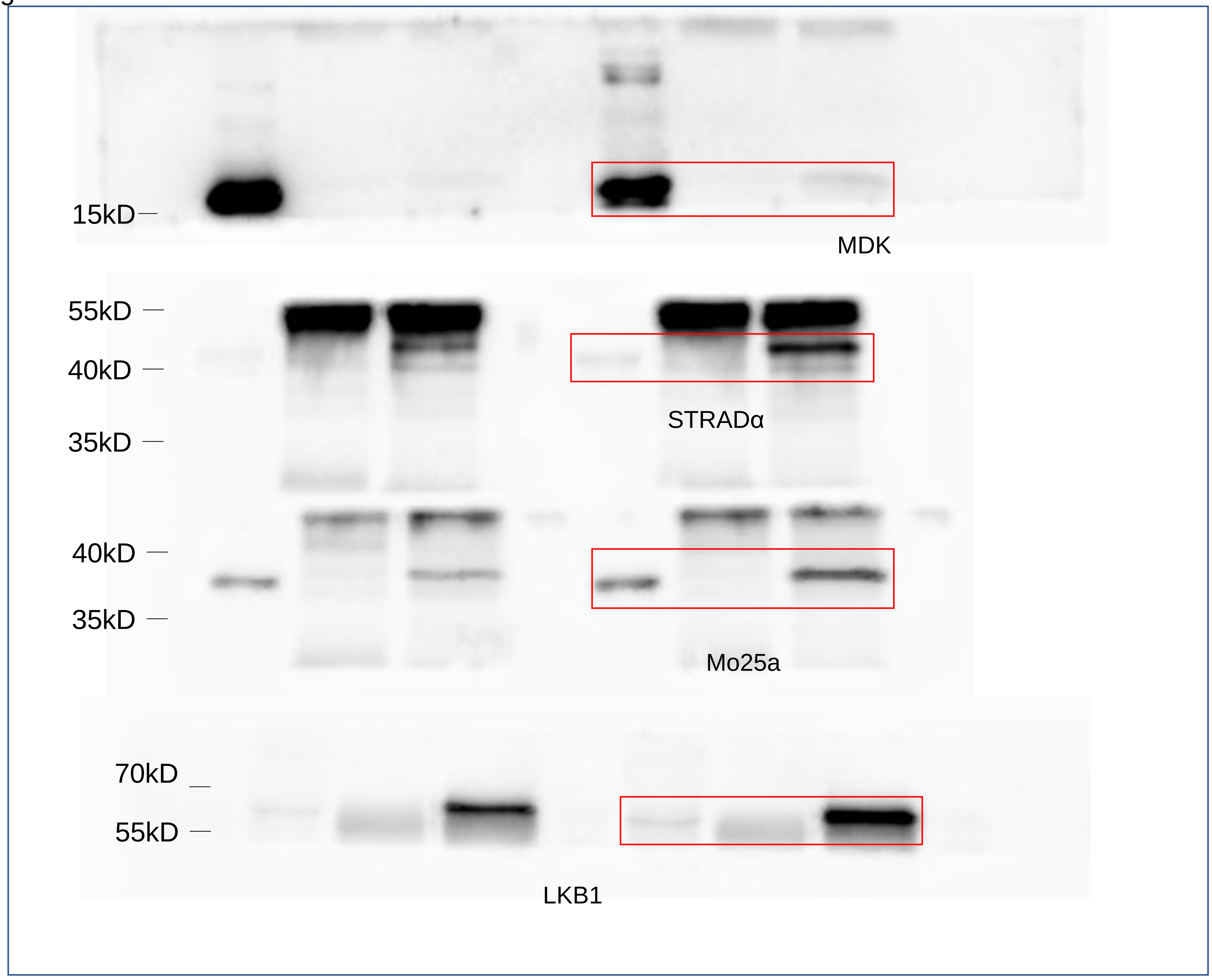

Fig.2G-H

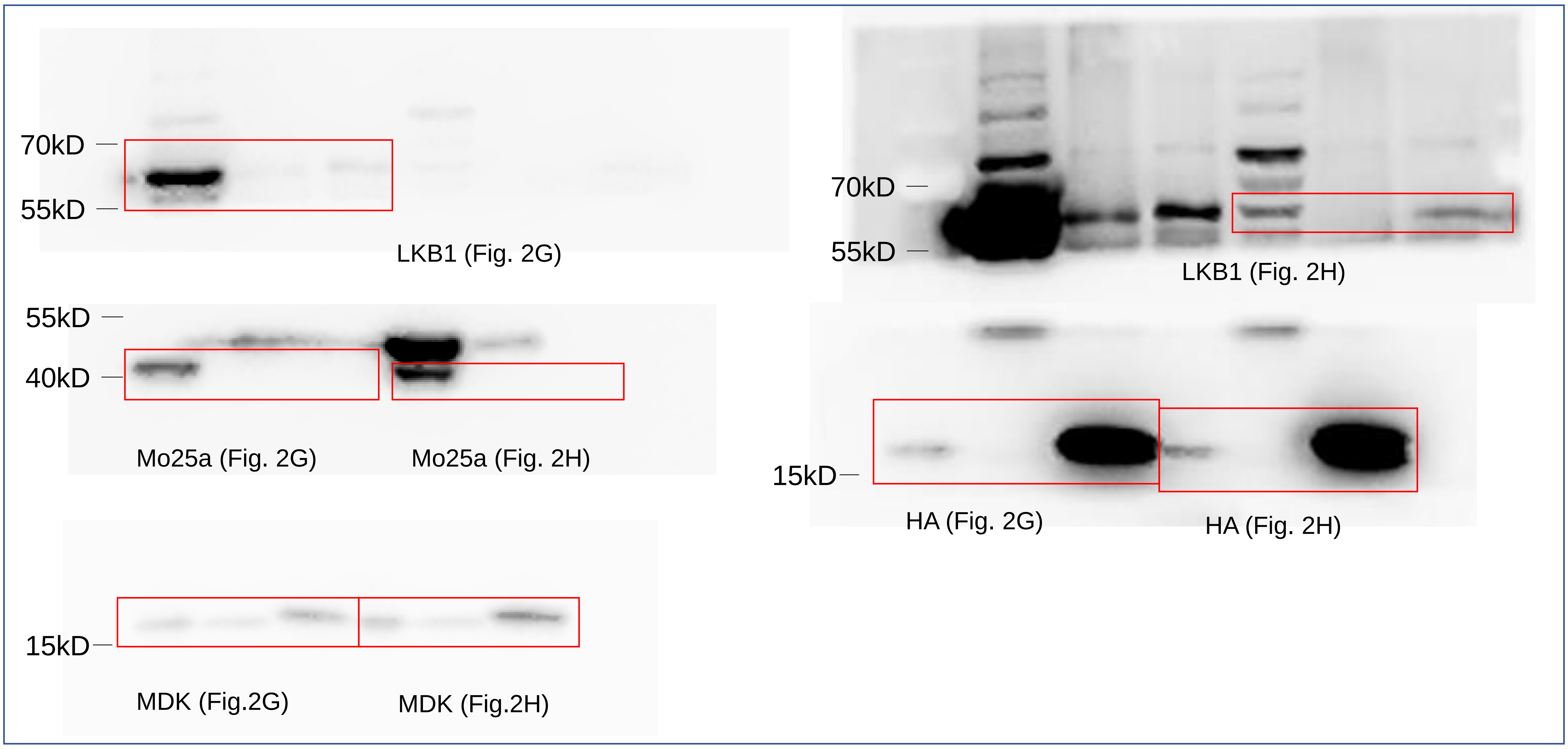


Fig.3A

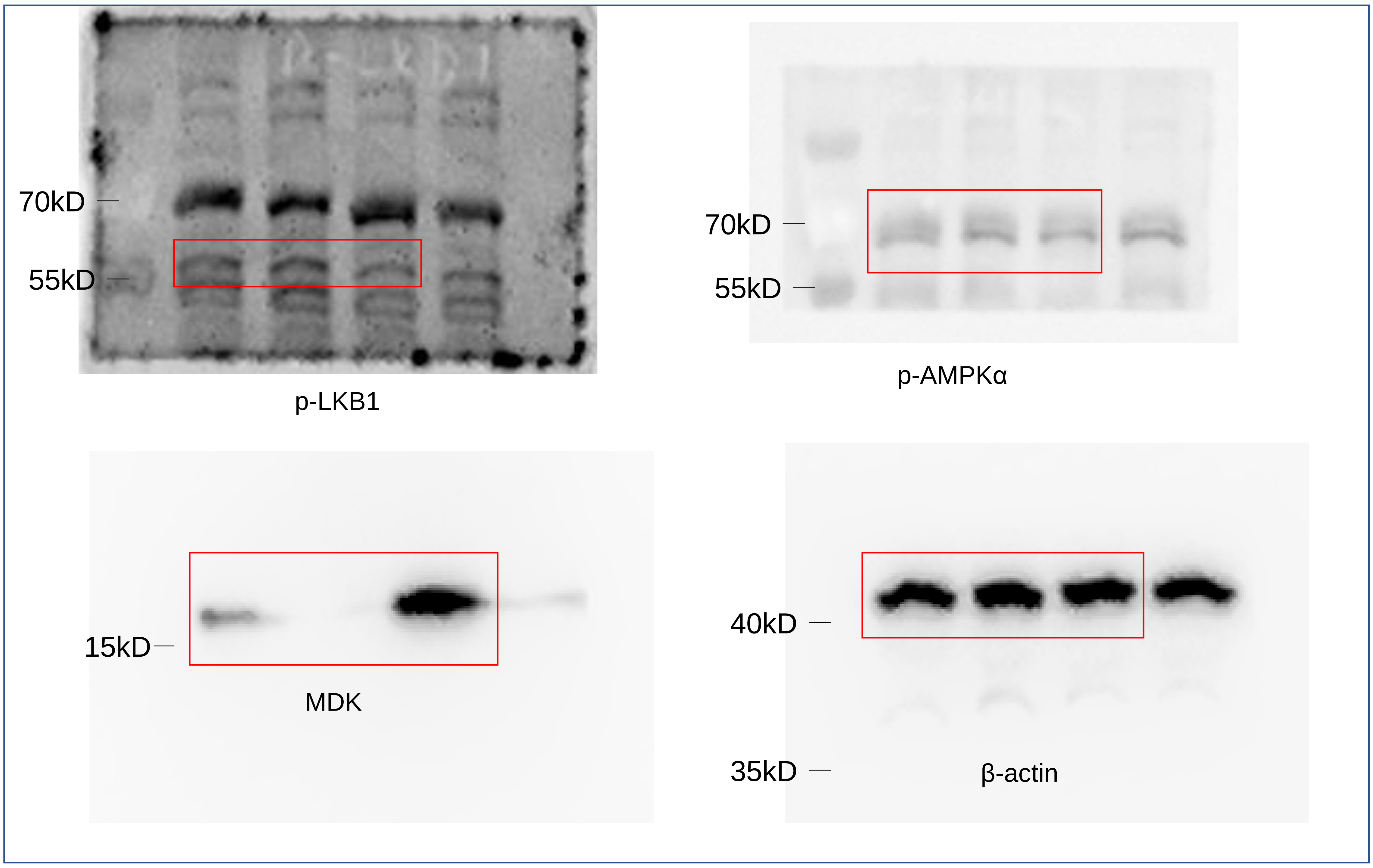

Fig.3B

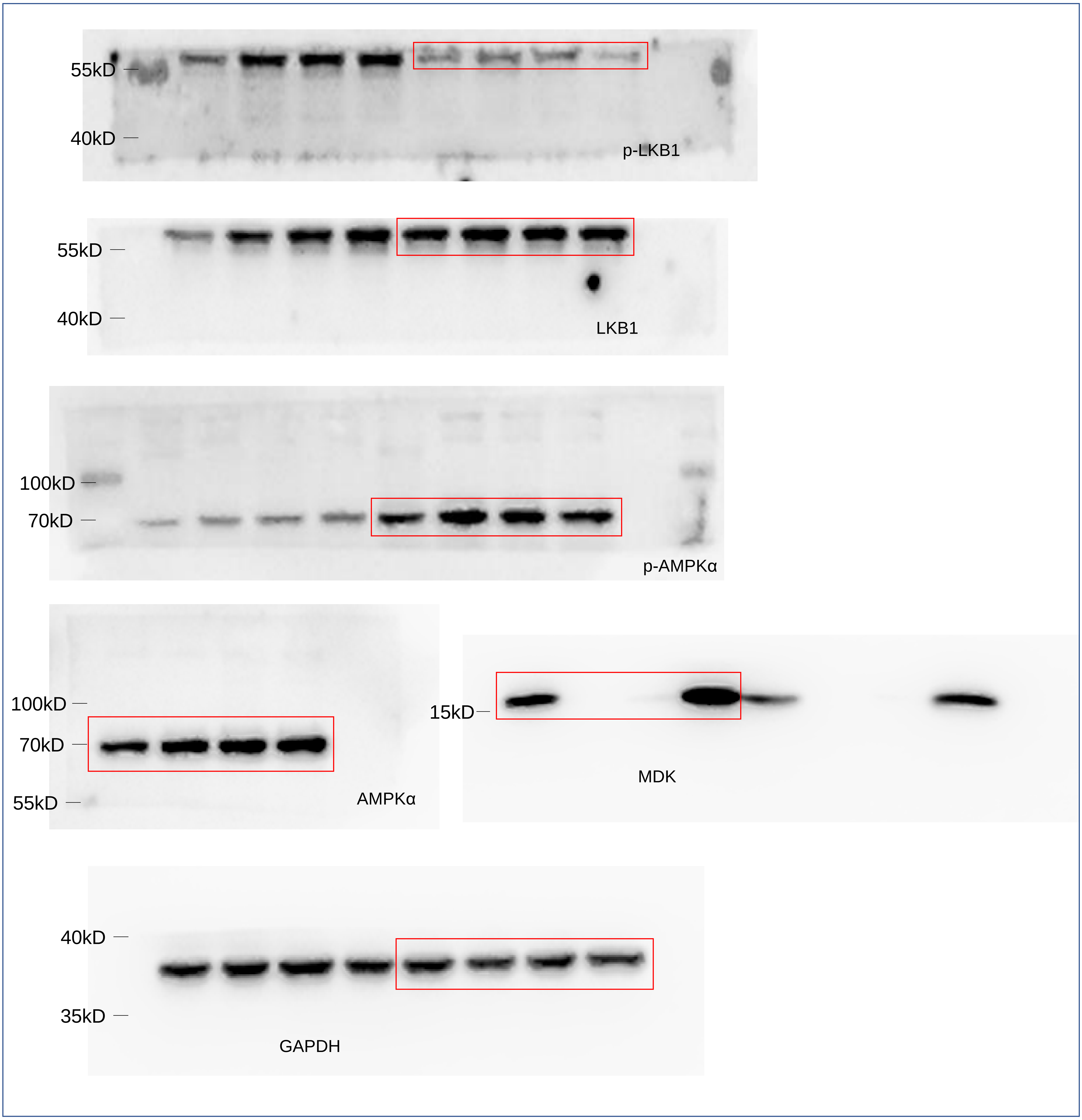
55kD
40kD
p-LKB1
55kD
40kD
LKB1
100kD
70kD
p-AMPKα
100kD
70kD
55kD
AMPKα
15kD
MDK
40kD
35kD
GAPDH

Fig.3C

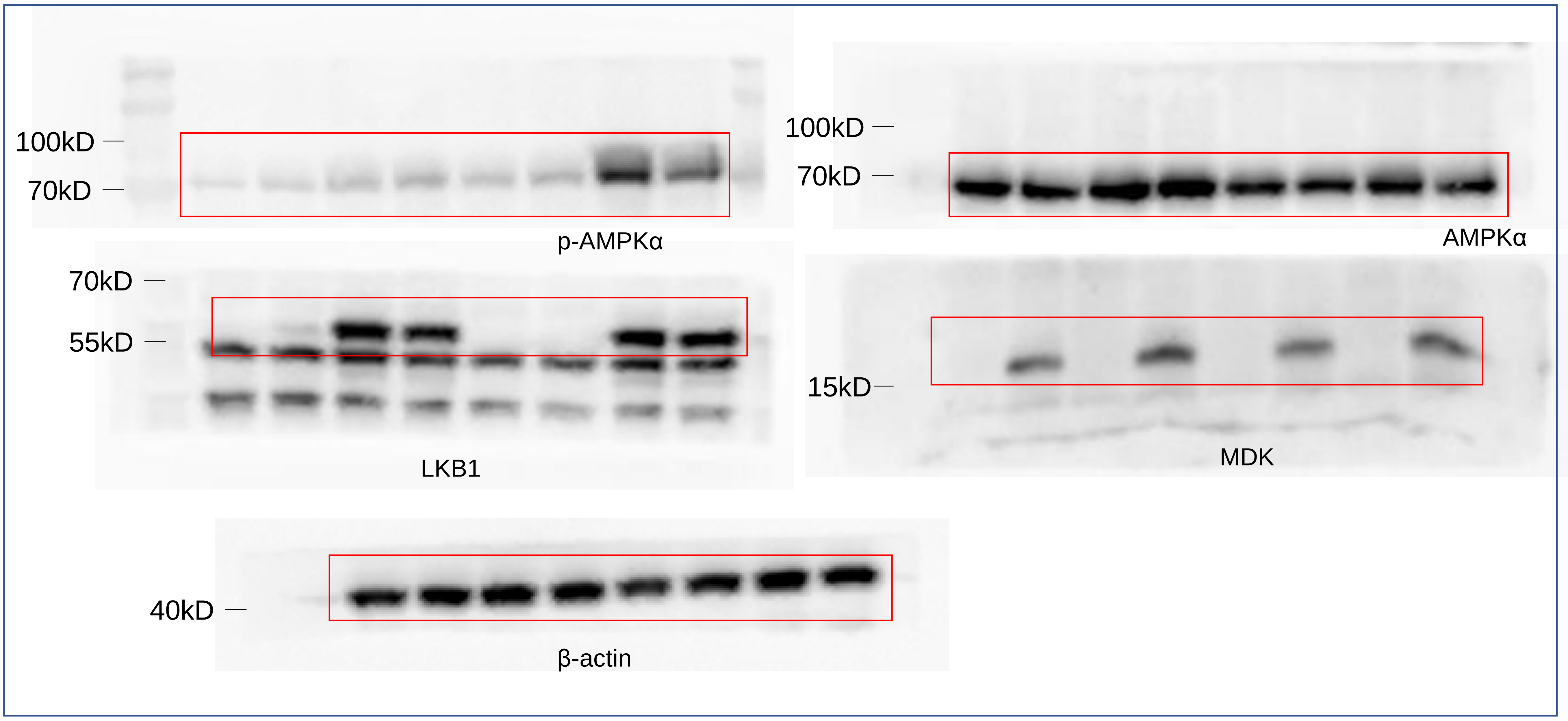
100kD
70kD
p-AMPKα
100kD
70kD
AMPKα
70kD
55kD
LKB1
15kD
MDK
40kD
β-actin

Fig.3D

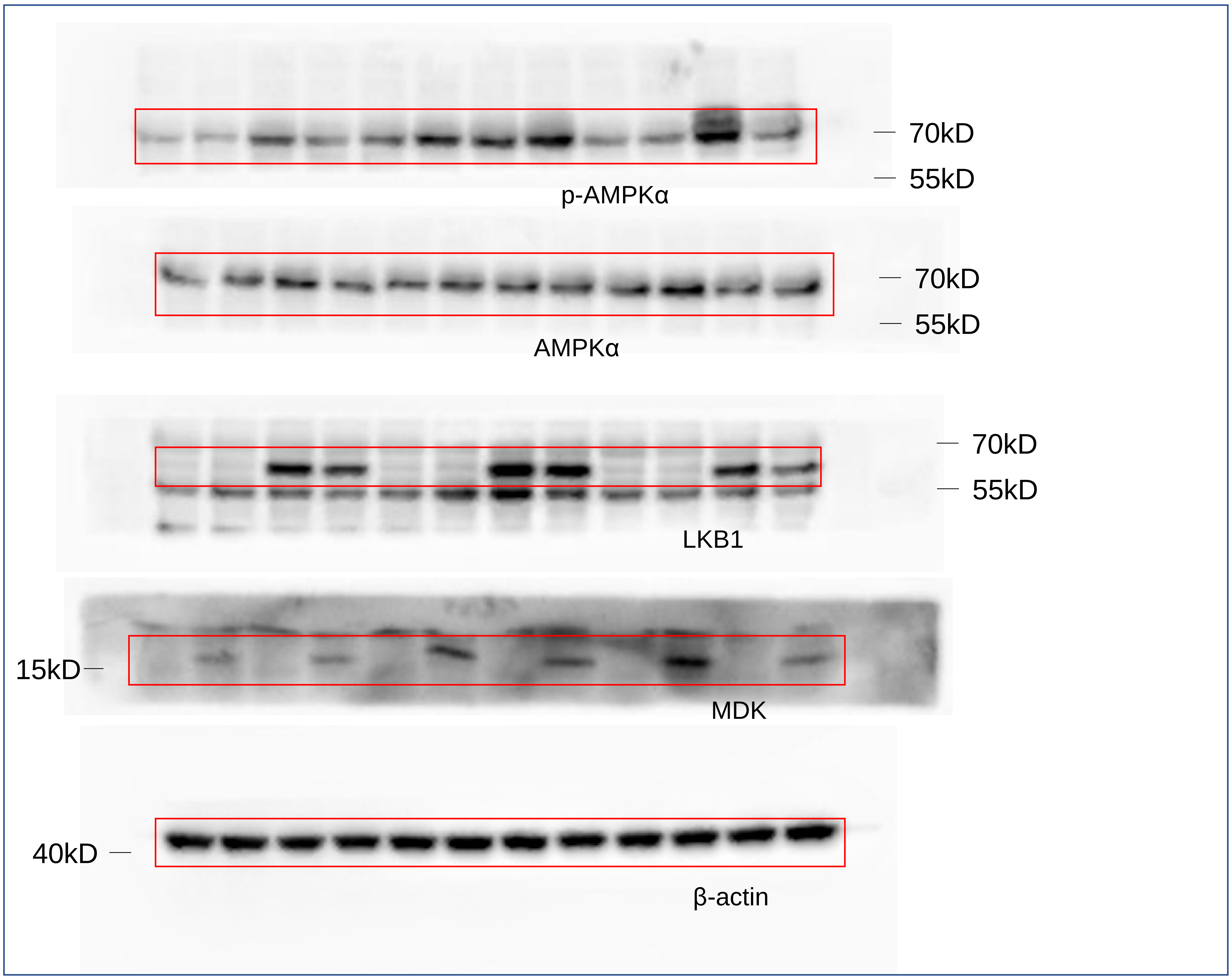
70kD
55kD
p-AMPKα
70kD
55kD
AMPKα
70kD
55kD
LKB1
15kD
MDK
40kD
β-actin

Fig.3E

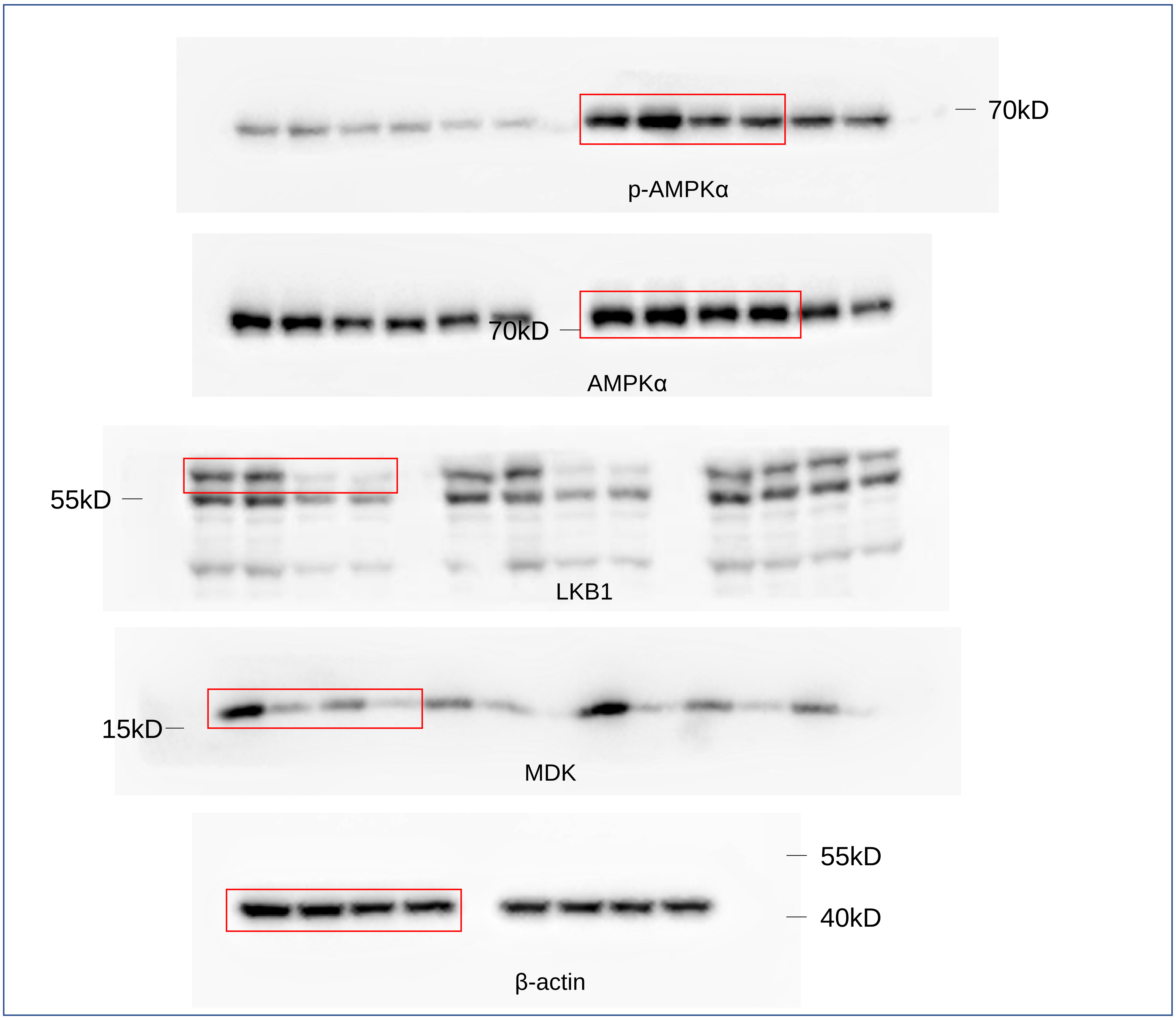
70kD
p-AMPKα
70kD
AMPKα
55kD
LKB1
15kD
MDK
55kD
40kD
β-actin

Fig.3F

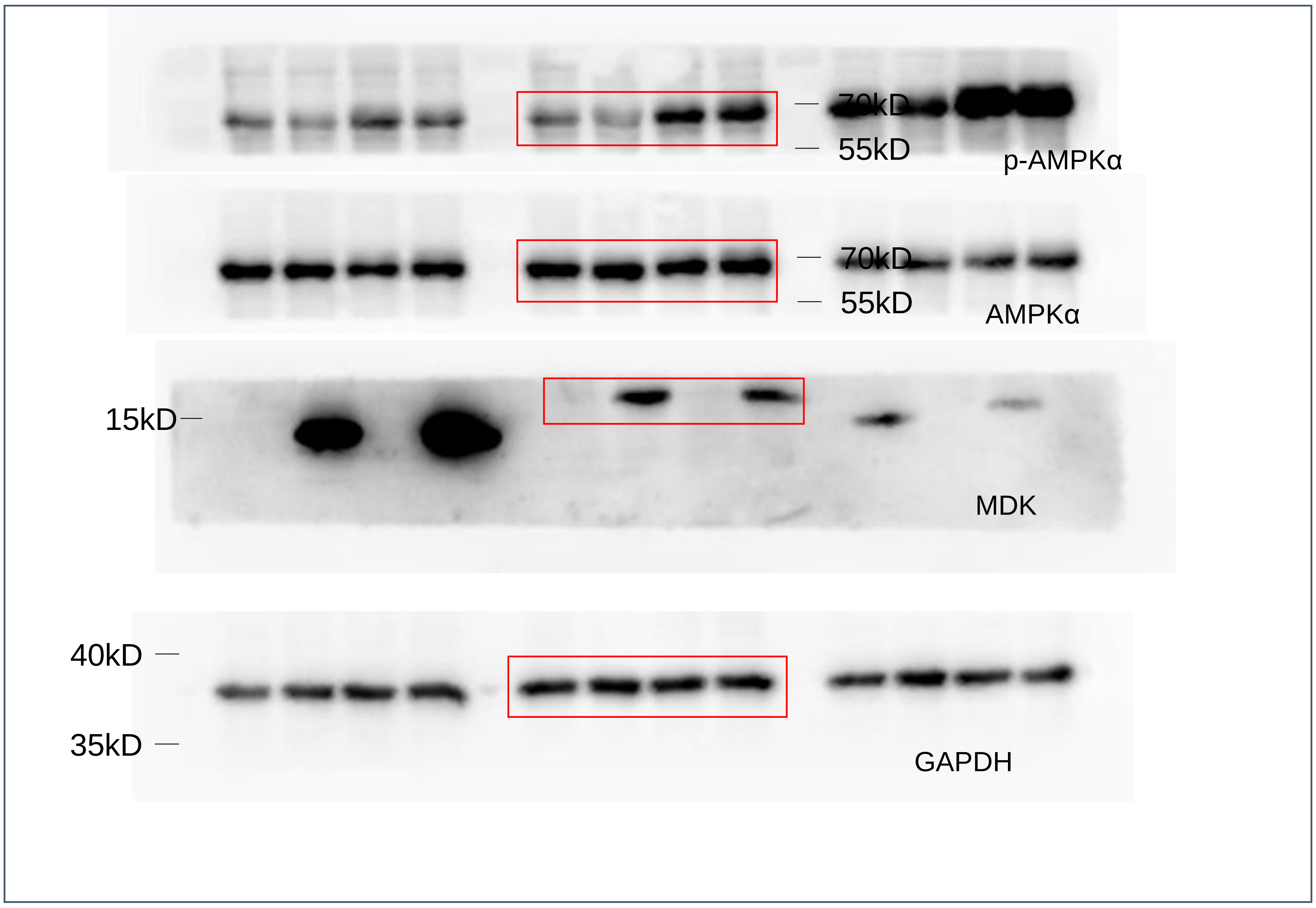
70kD
55kD
p-AMPKα
70kD
55kD
AMPKα
15kD
MDK
40kD
35kD
GAPDH

Fig.4A

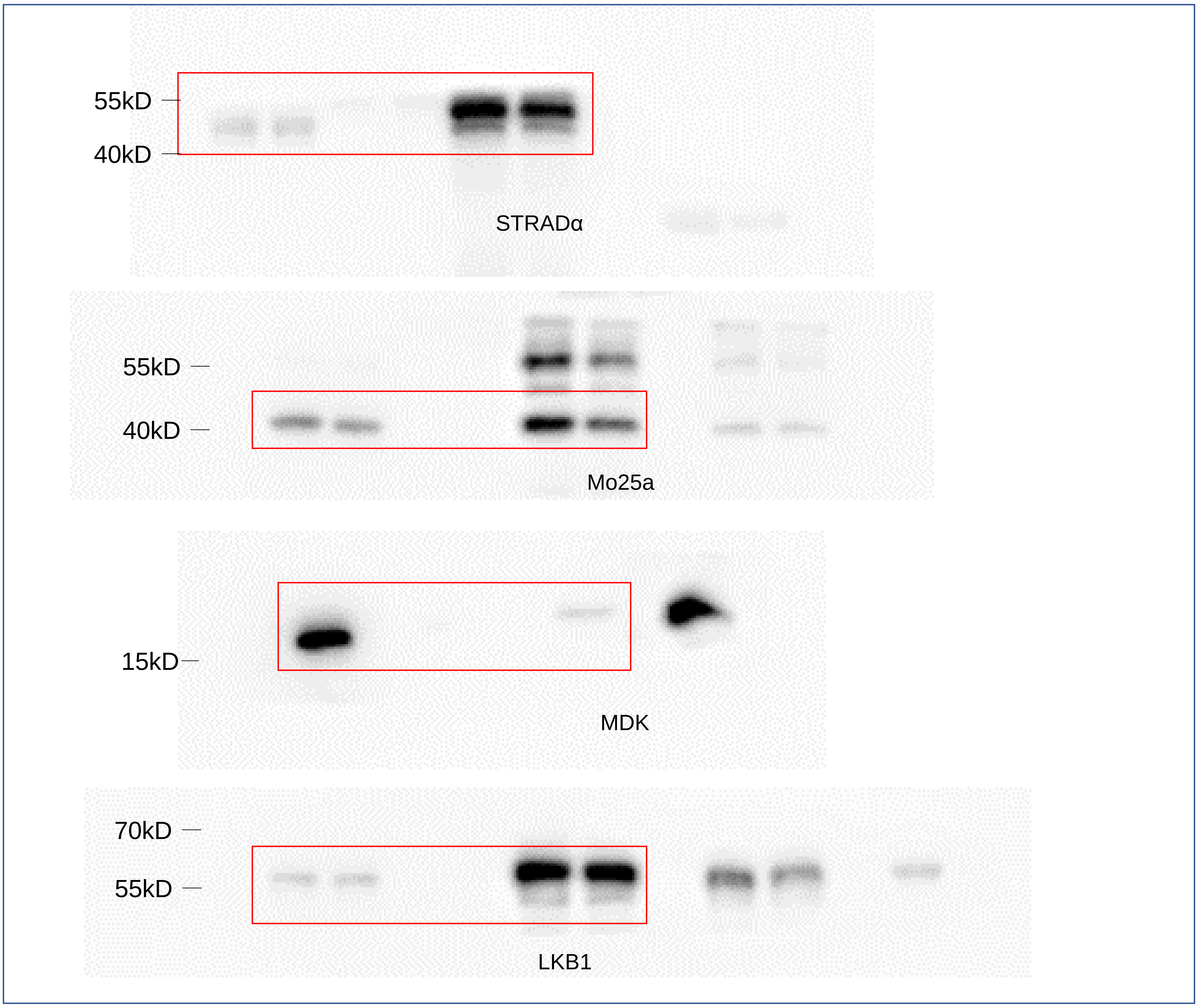

55kD
40kD
STRADα
55kD
40kD
Mo25a
15kD
MDK
70kD
55kD
LKB1


Fig.4B

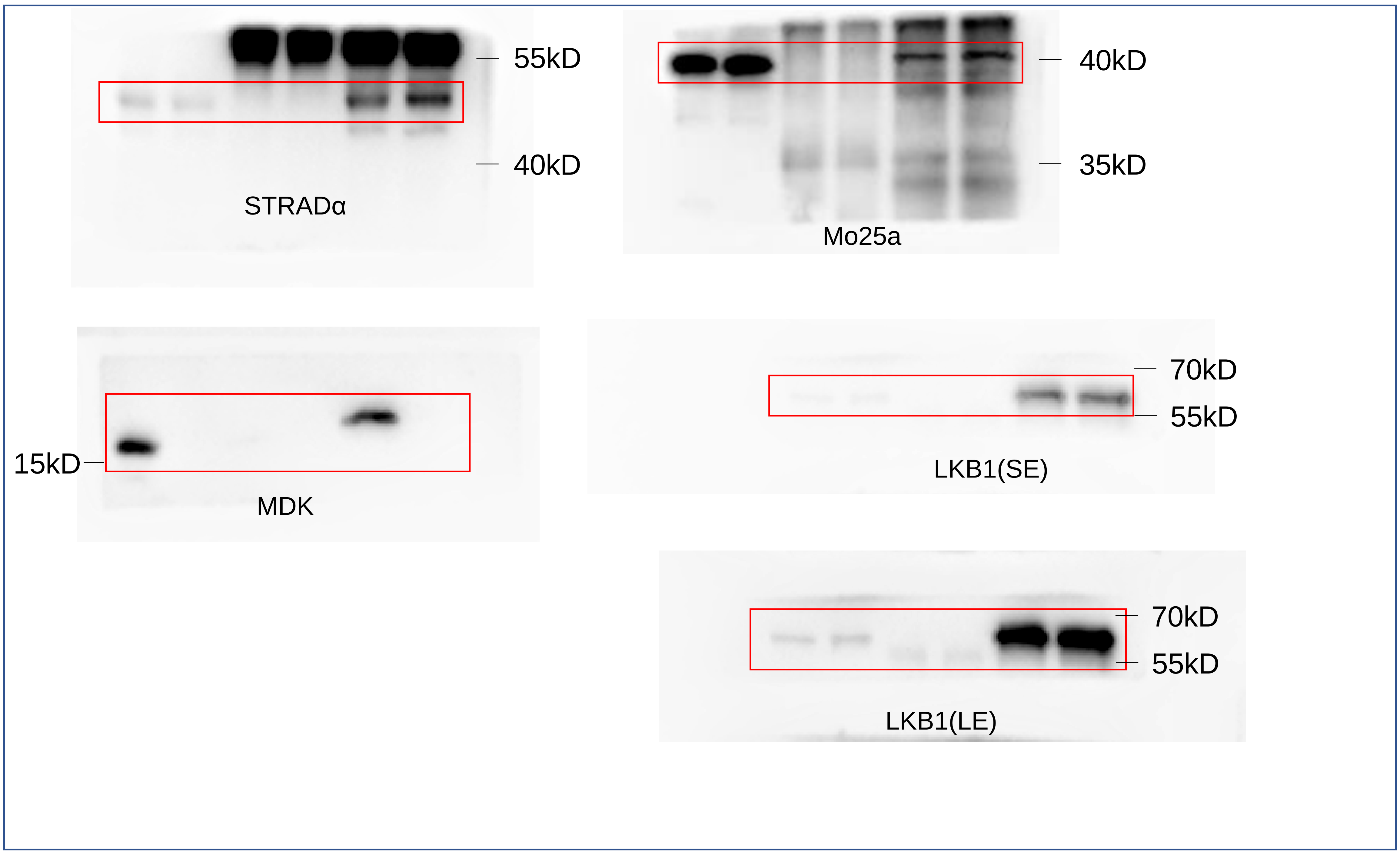

55kD
40kD
STRADα
40kD
35kD
Mo25a
15kD
MDK
70kD
55kD
LKB1(SE)
70kD
55kD
LKB1(LE)

Fig.4C

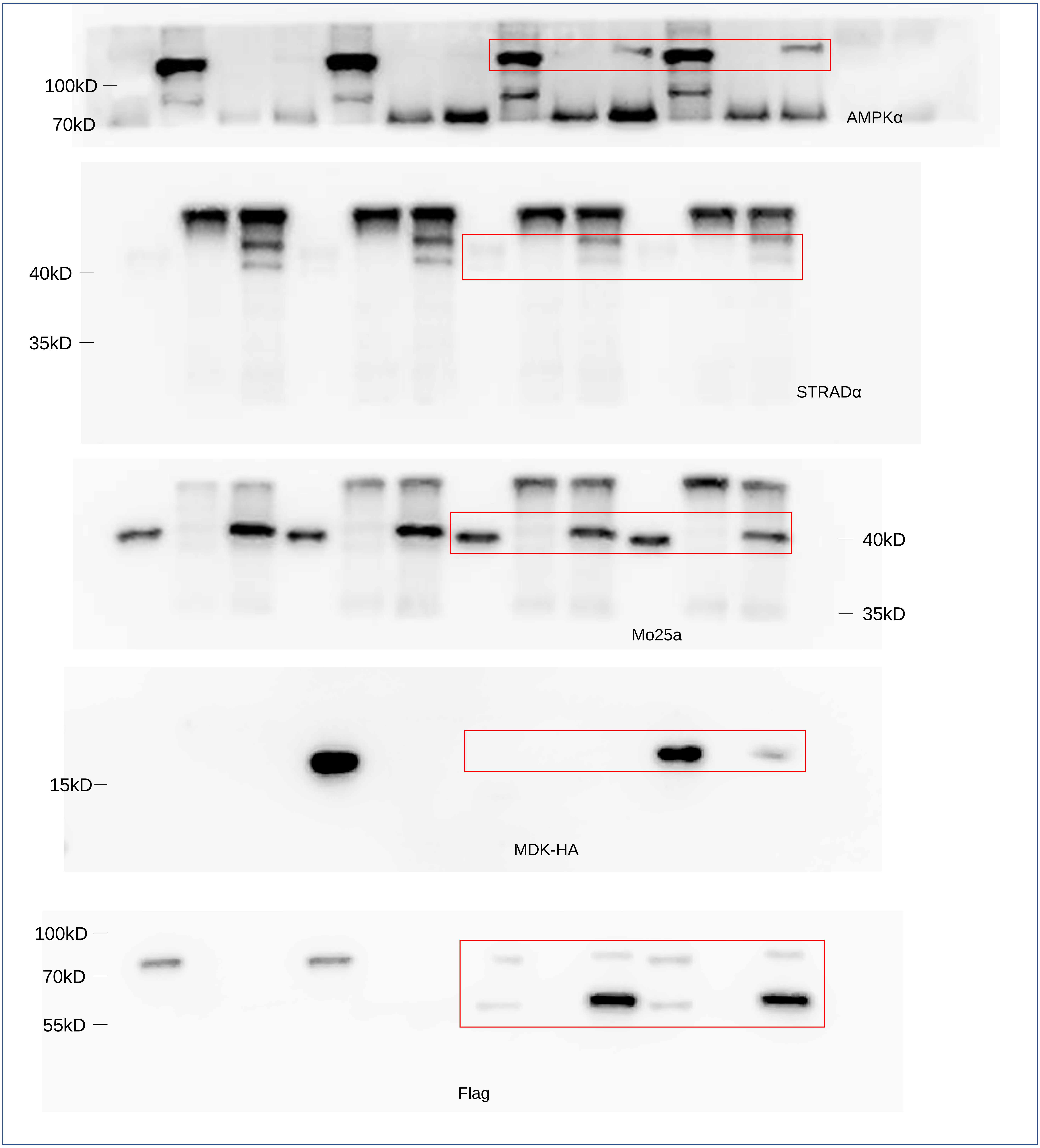
100kD
70kD
AMPKα
40kD
35kD
STRADα
40kD
35kD
Mo25a
15kD
MDK-HA
100kD
70kD
55kD
Flag

Fig.4D

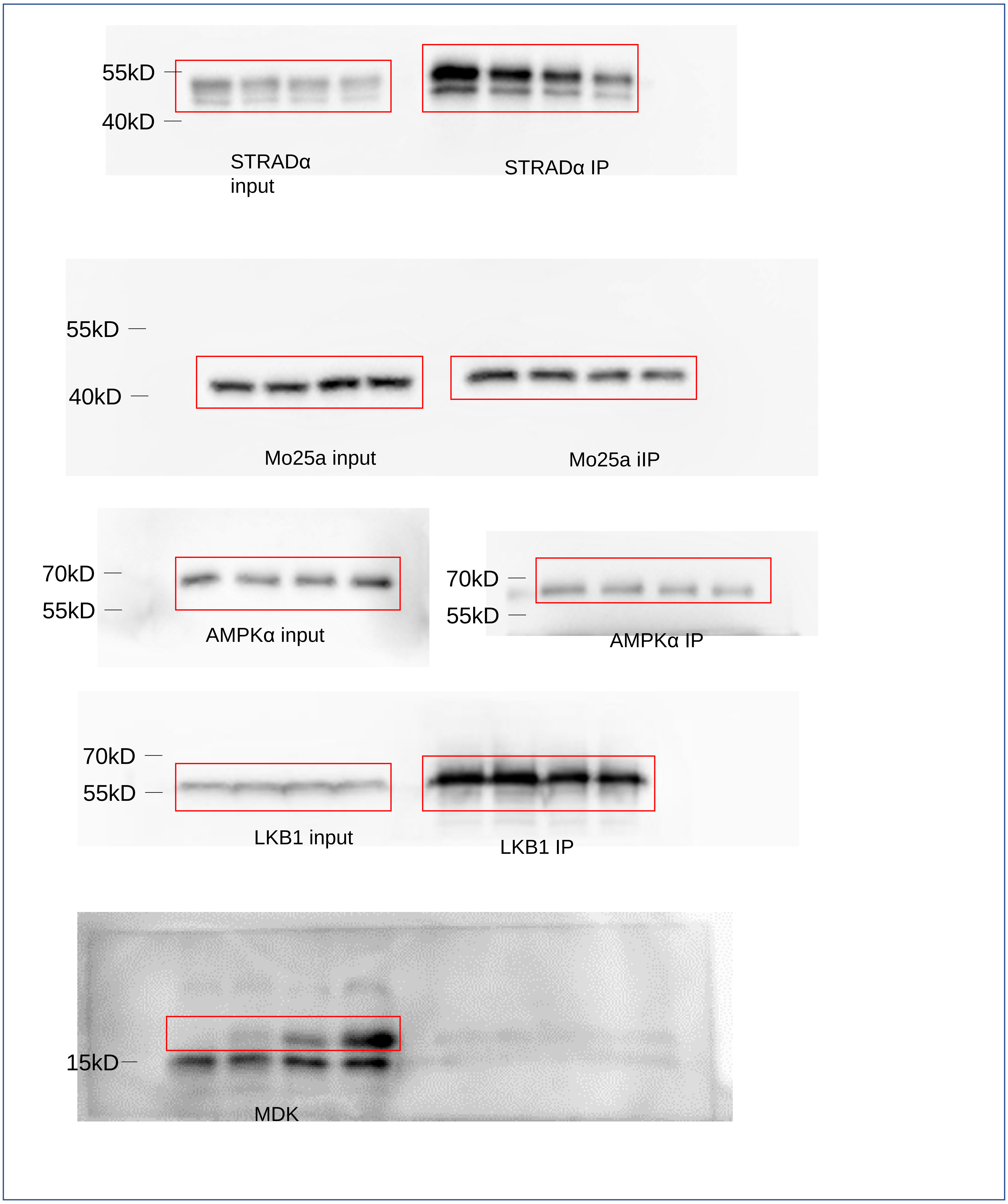
55kD
40kD
STRADα input
STRADα IP
55kD
40kD
Mo25a input
Mo25a iIP
70kD
55kD
AMPKα input
70kD
55kD
AMPKα IP
70kD
55kD
LKB1 input
LKB1 IP
15kD
MDK

Fig.4E

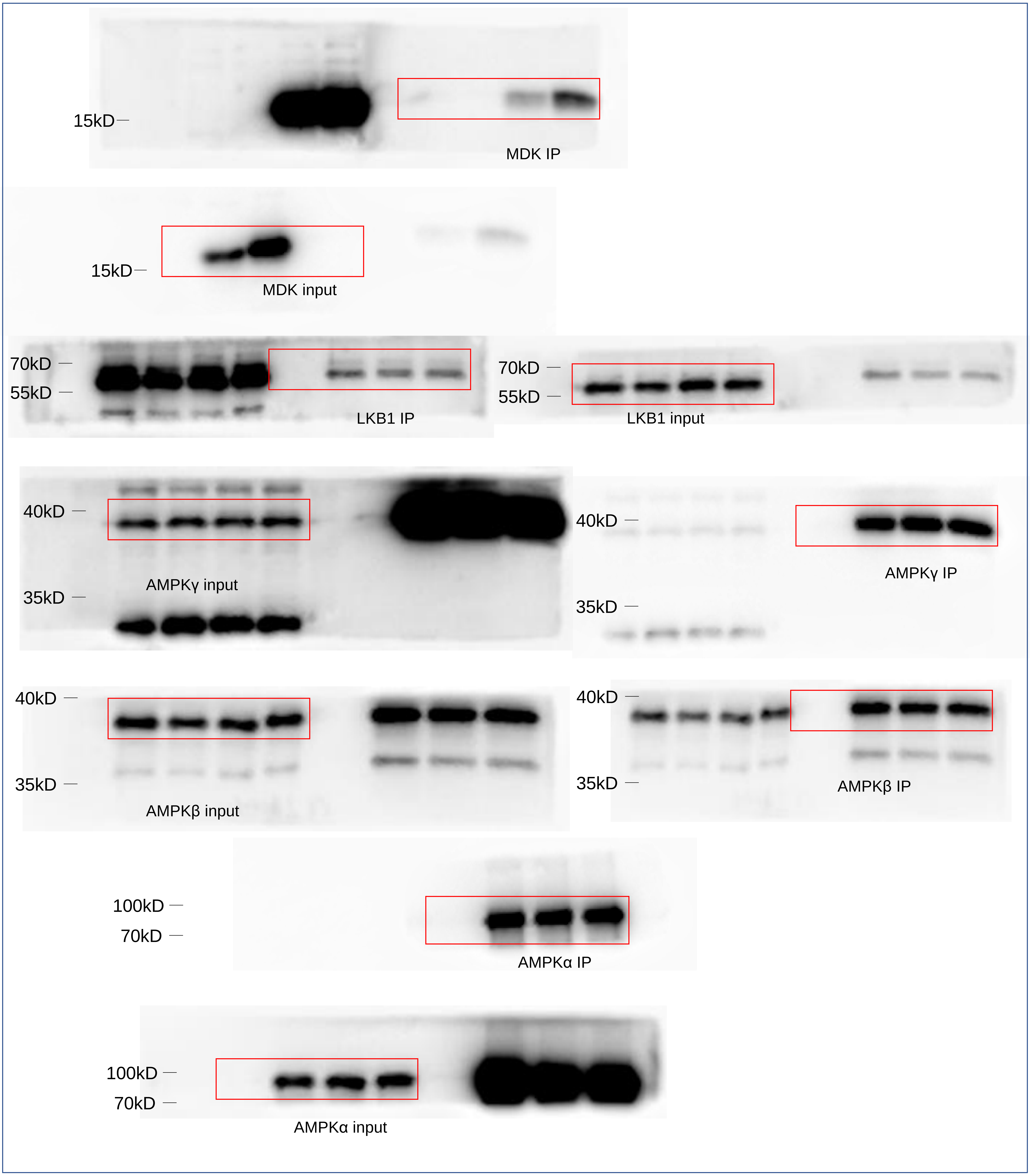

15kD
MDK IP
15kD
MDK input
70kD
55kD
LKB1 IP
70kD
55kD
LKB1 input
40kD
35kD
AMPKγ input
40kD
35kD
AMPKγ IP
40kD
35kD
AMPKβ input
40kD
35kD
AMPKβ IP
100kD
70kD
AMPKα IP
100kD
70kD
AMPKα input

Fig.5C

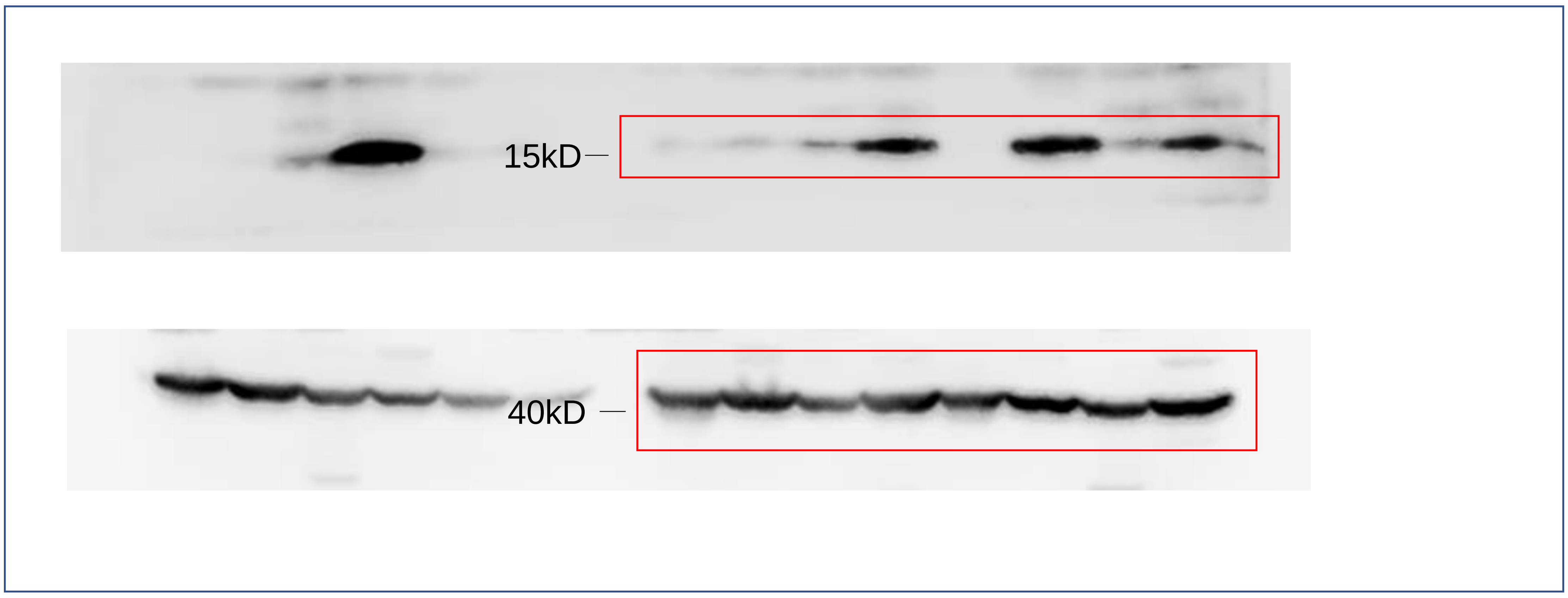

Fig.6A

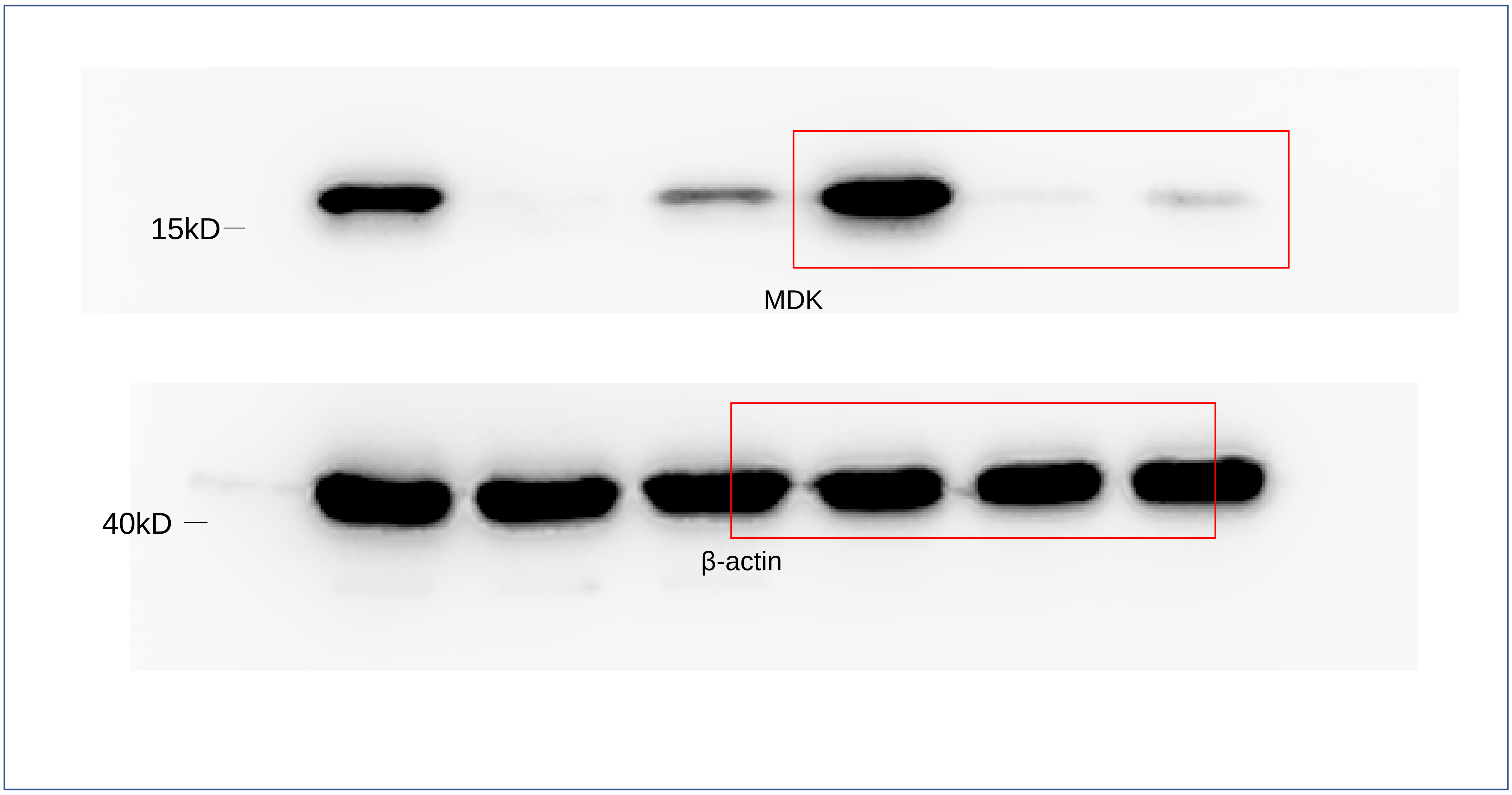


Fig.6C

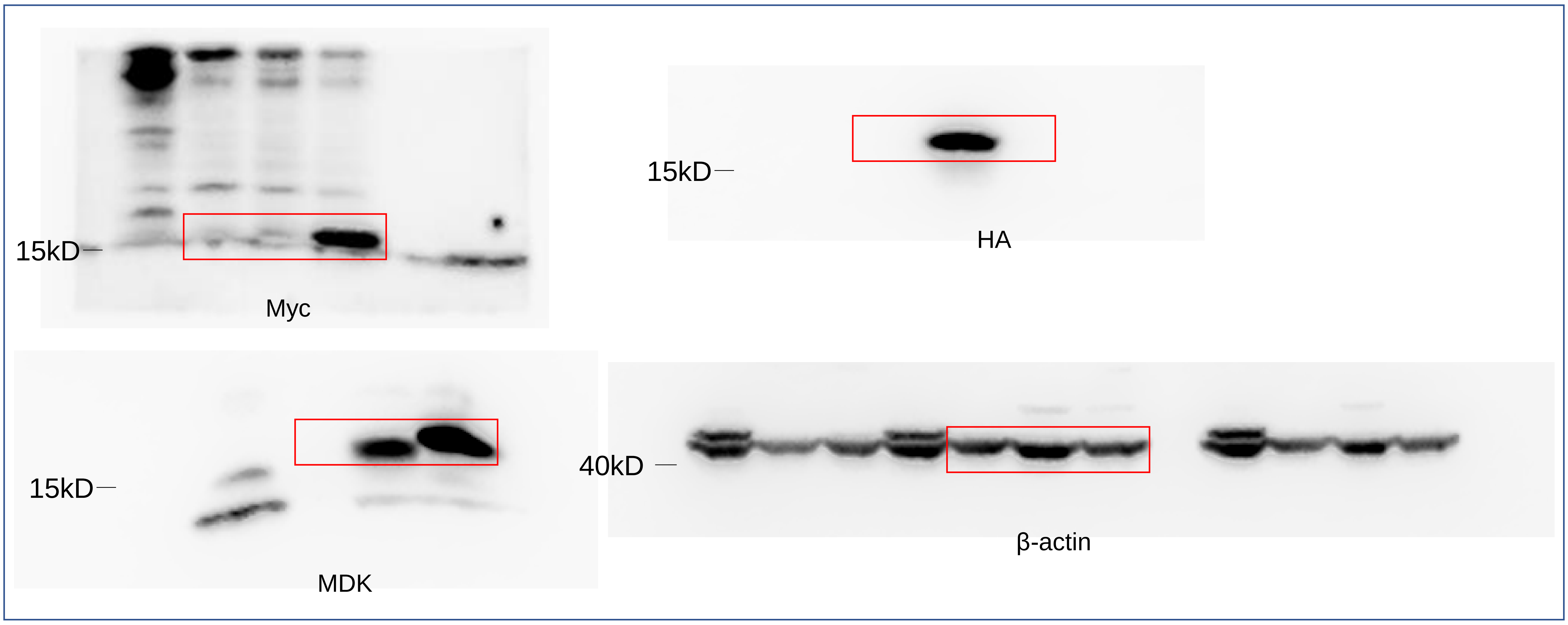


Fig.6E

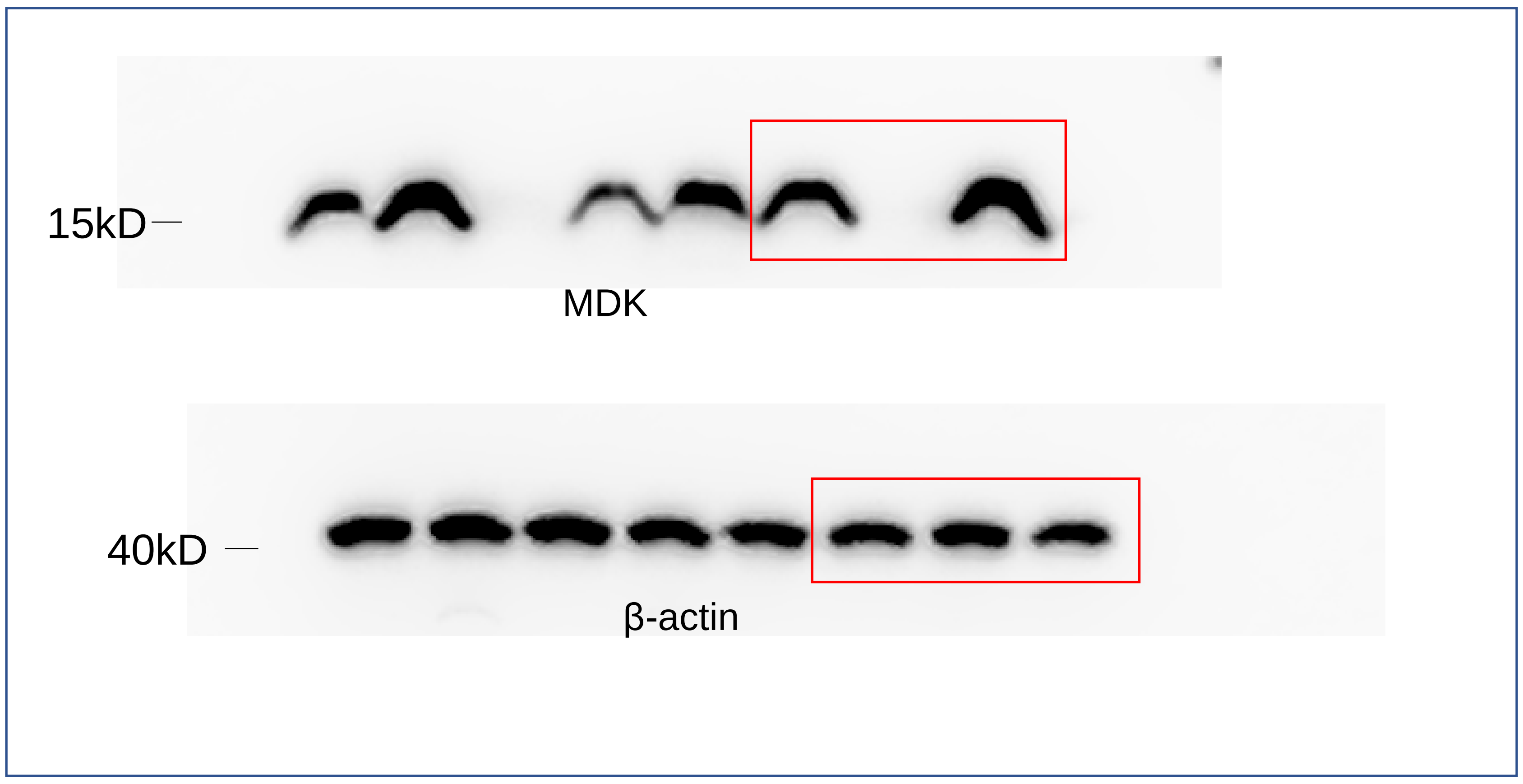

Fig.6G

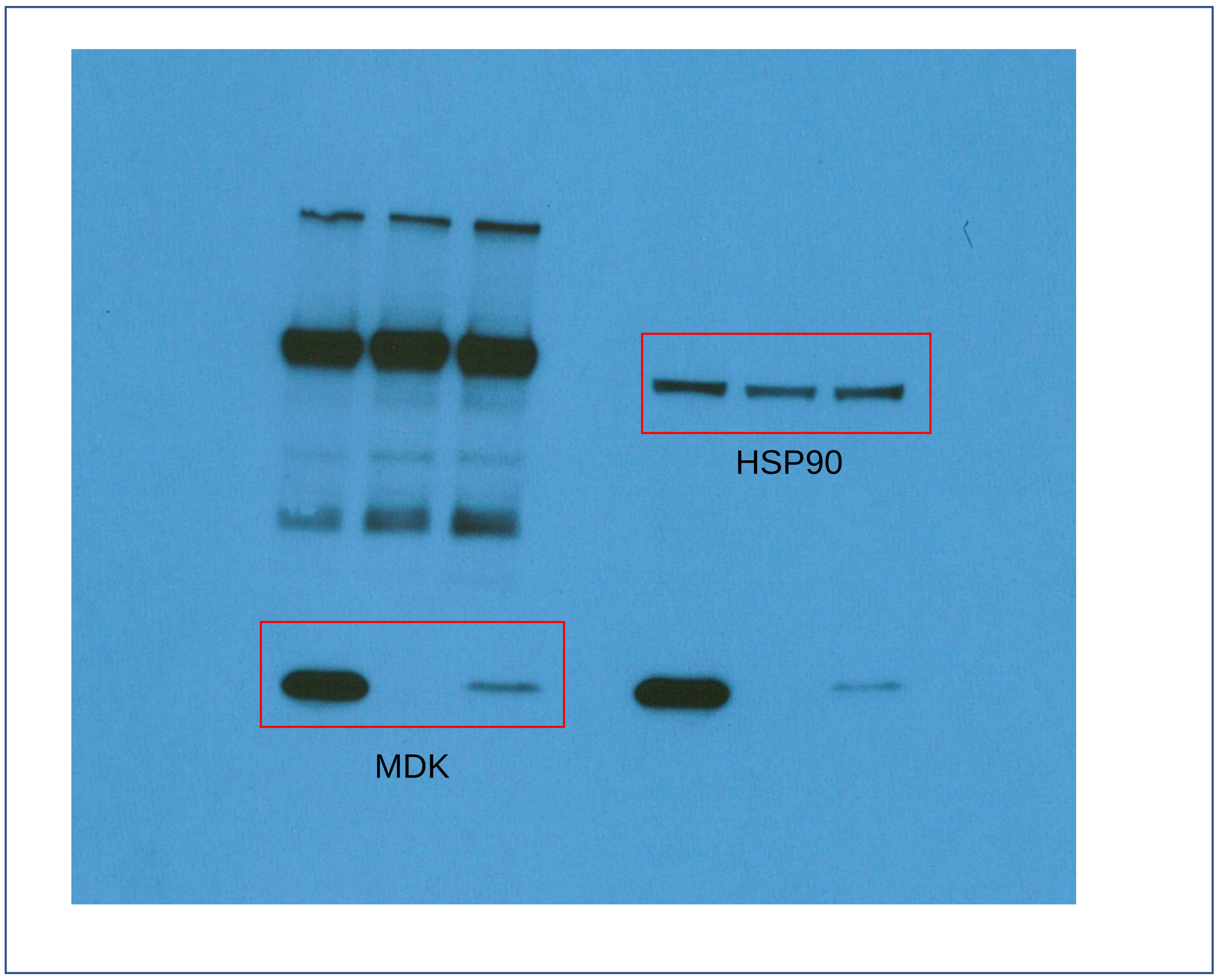


Fig.6H

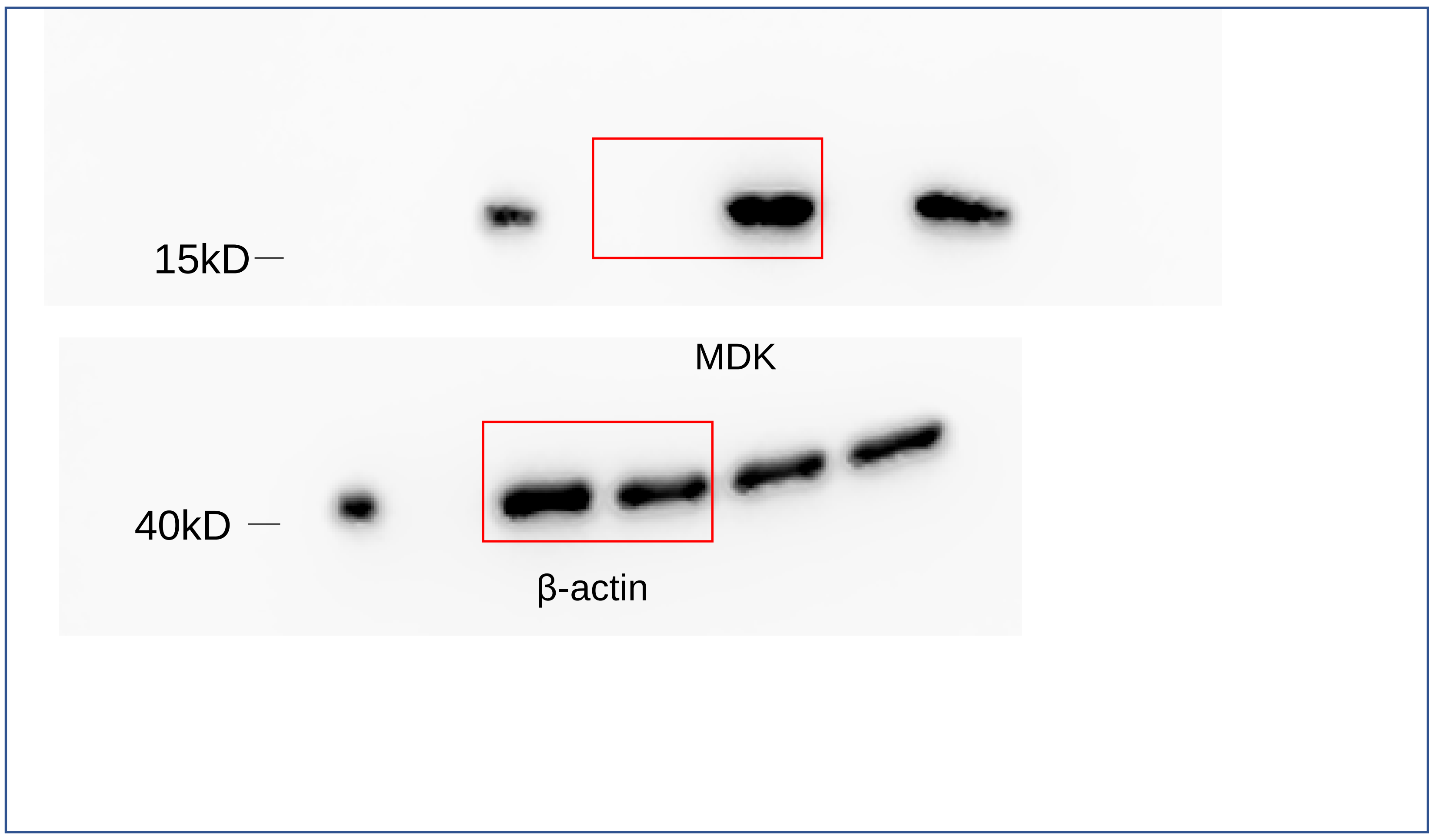


Figure 7a

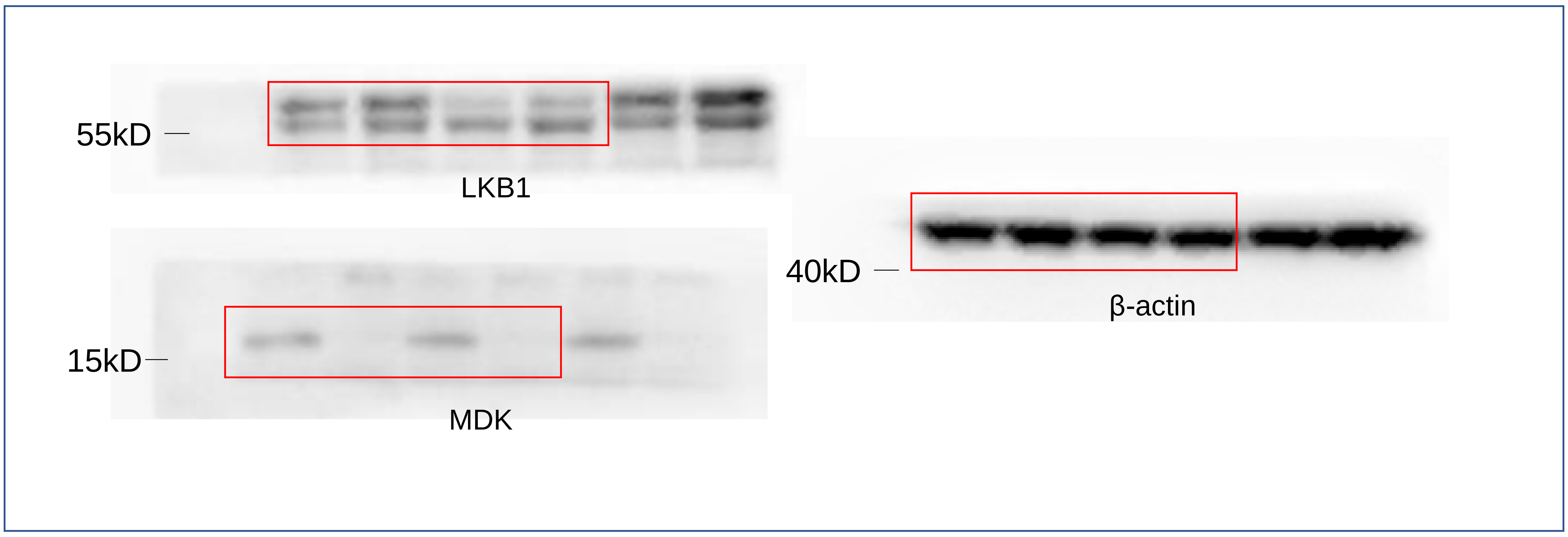

Supplemental Figure 1A,B

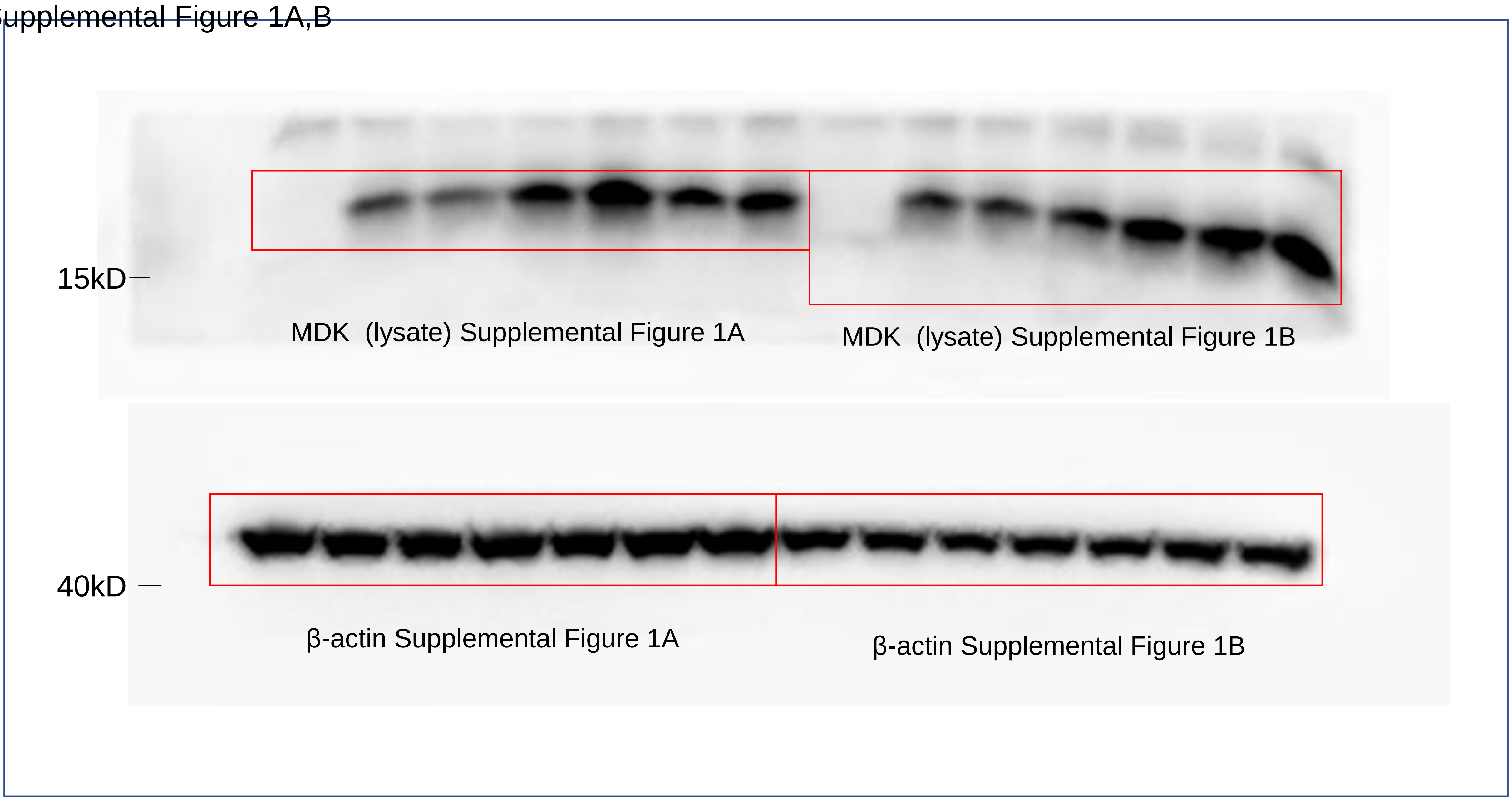


Supplemental Figure 1C

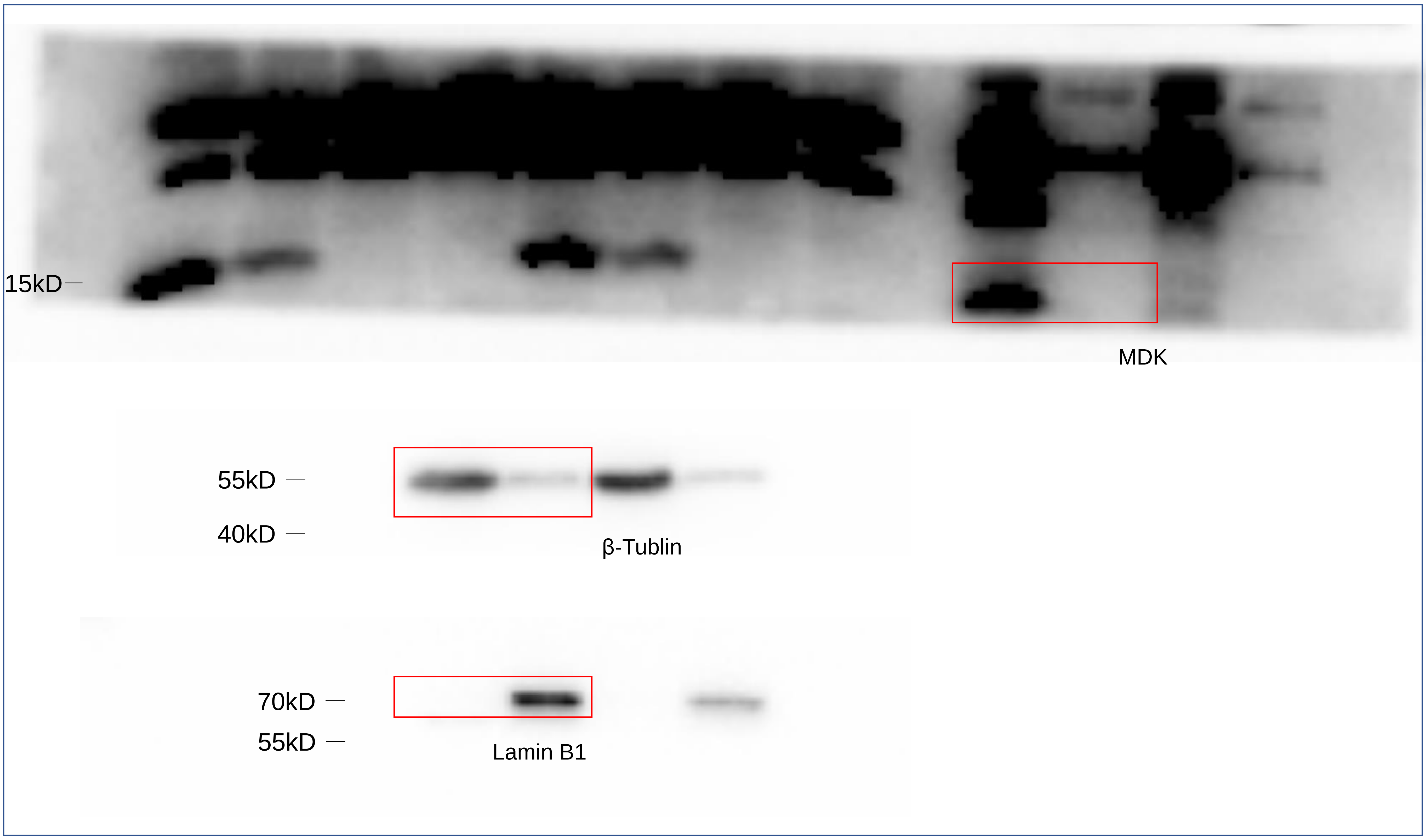

Supplemental Figure 1D

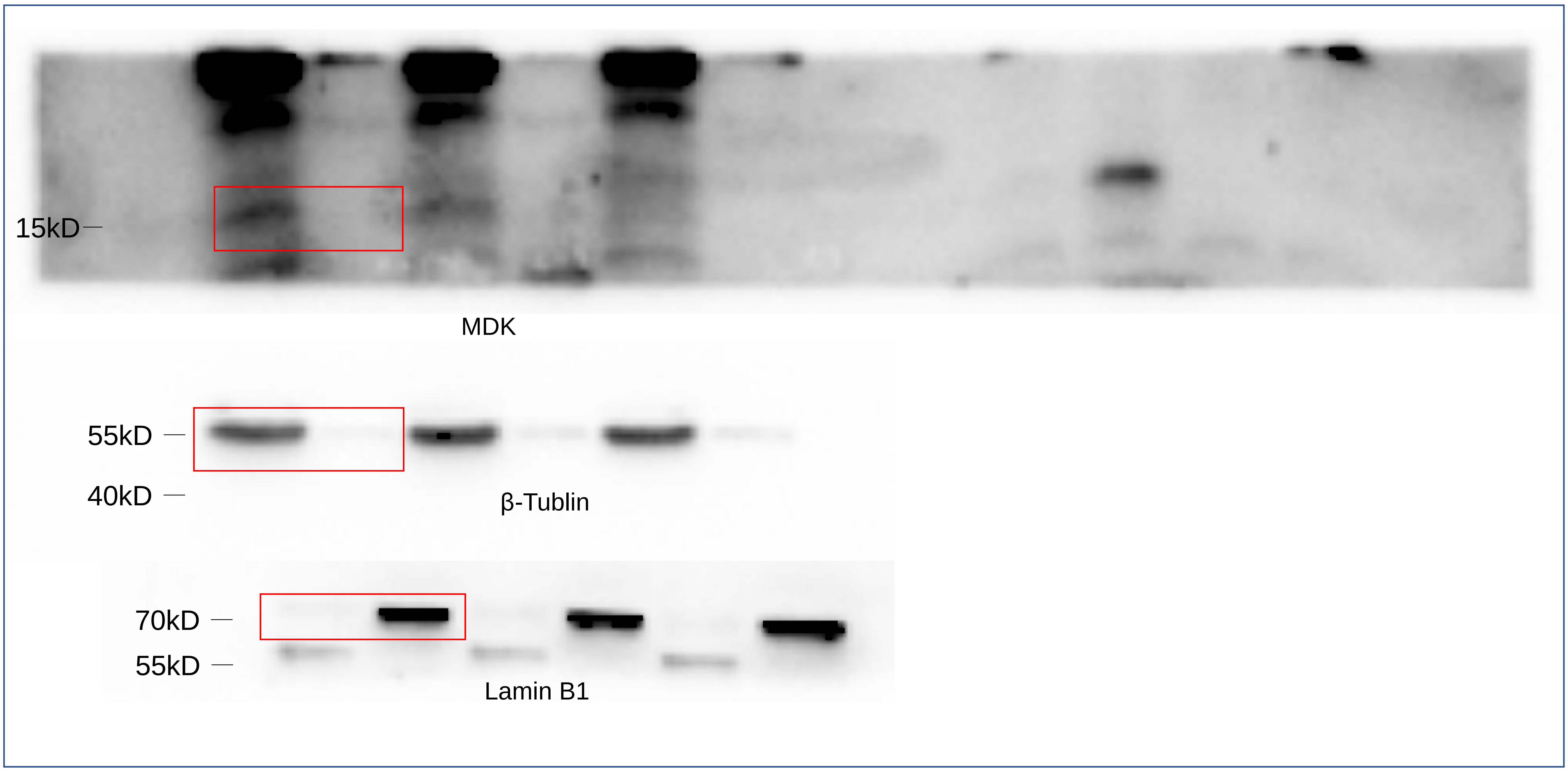


Supplemental Figure 1E

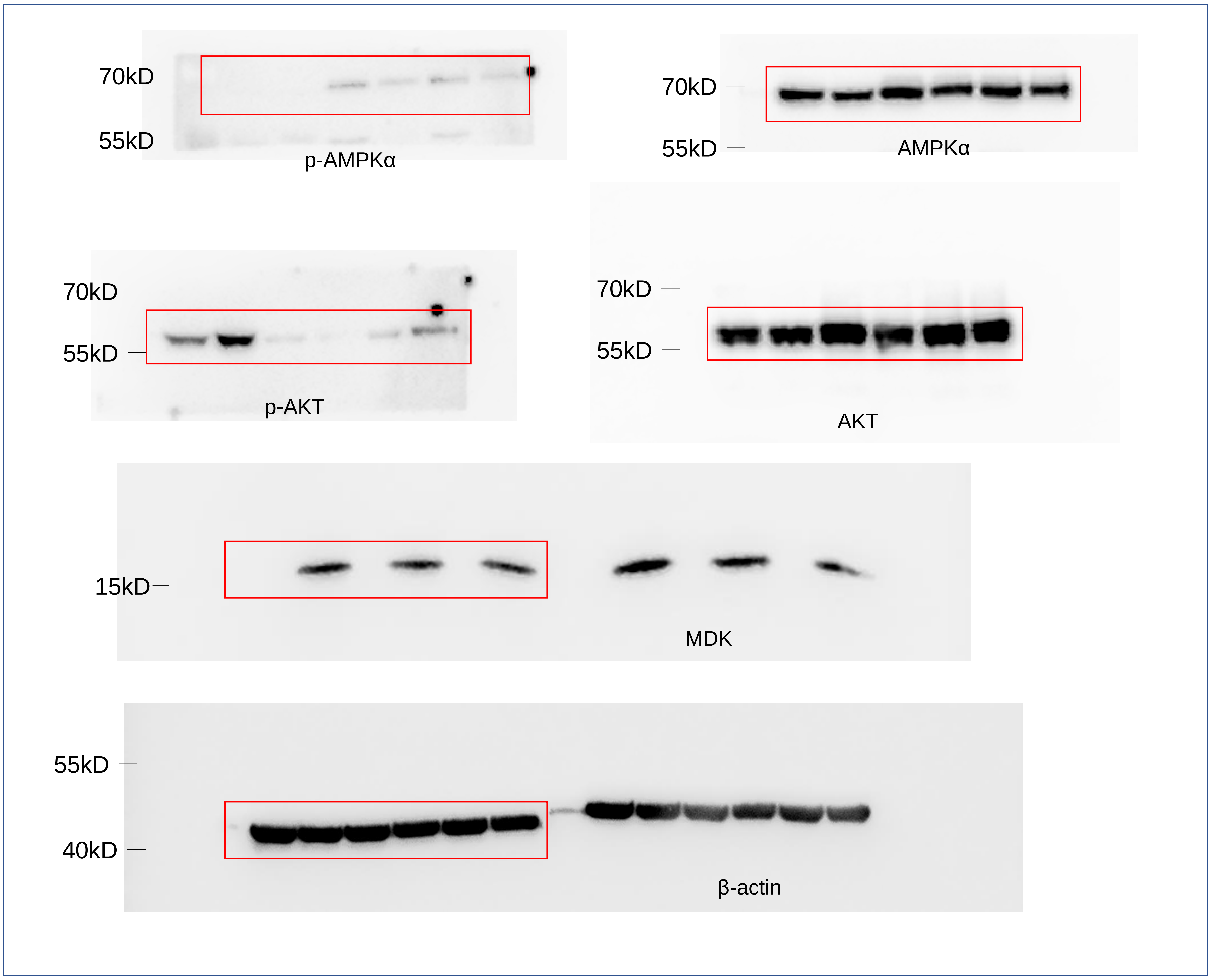

Supplemental Figure 1F

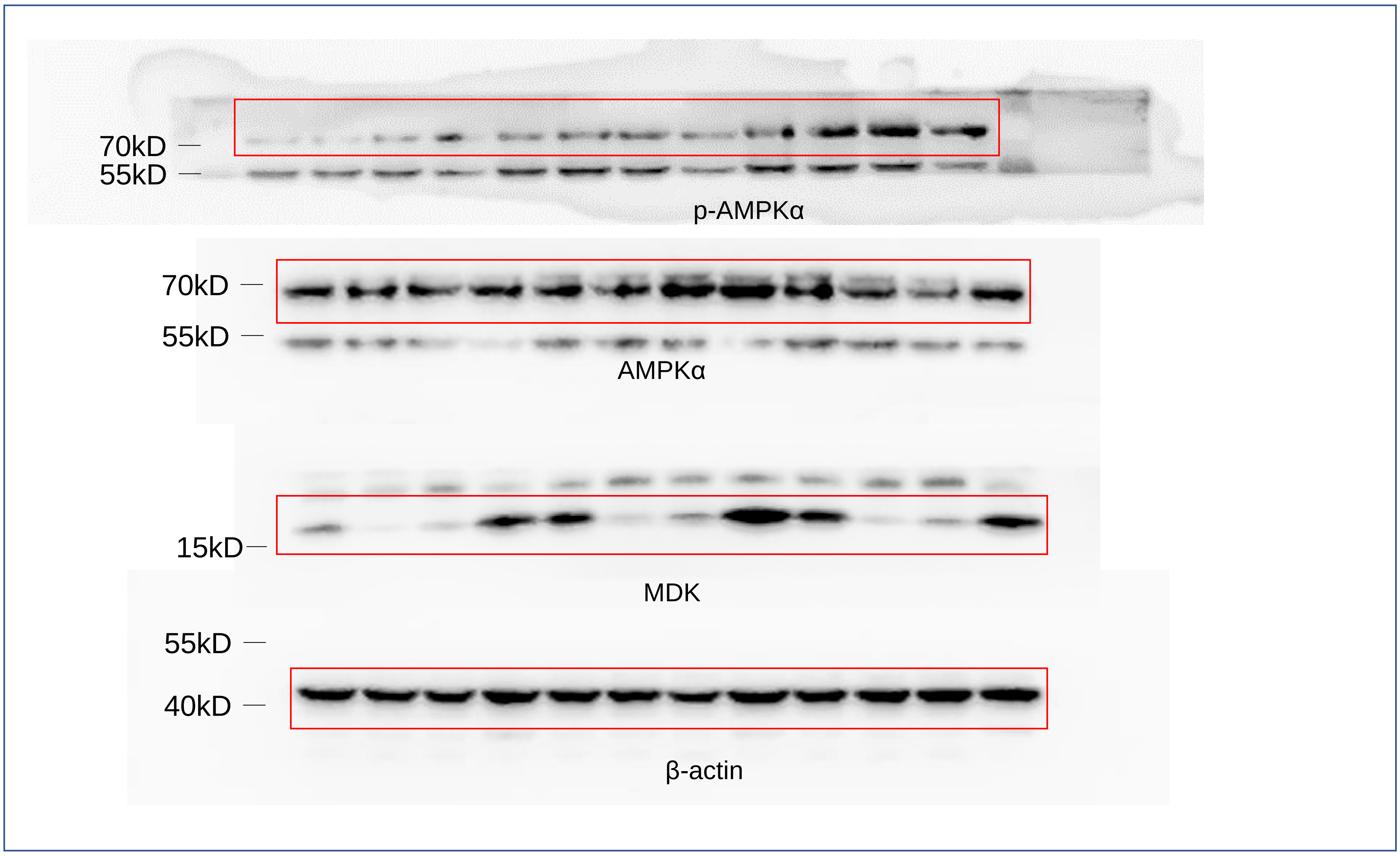


Supplemental Figure 1G

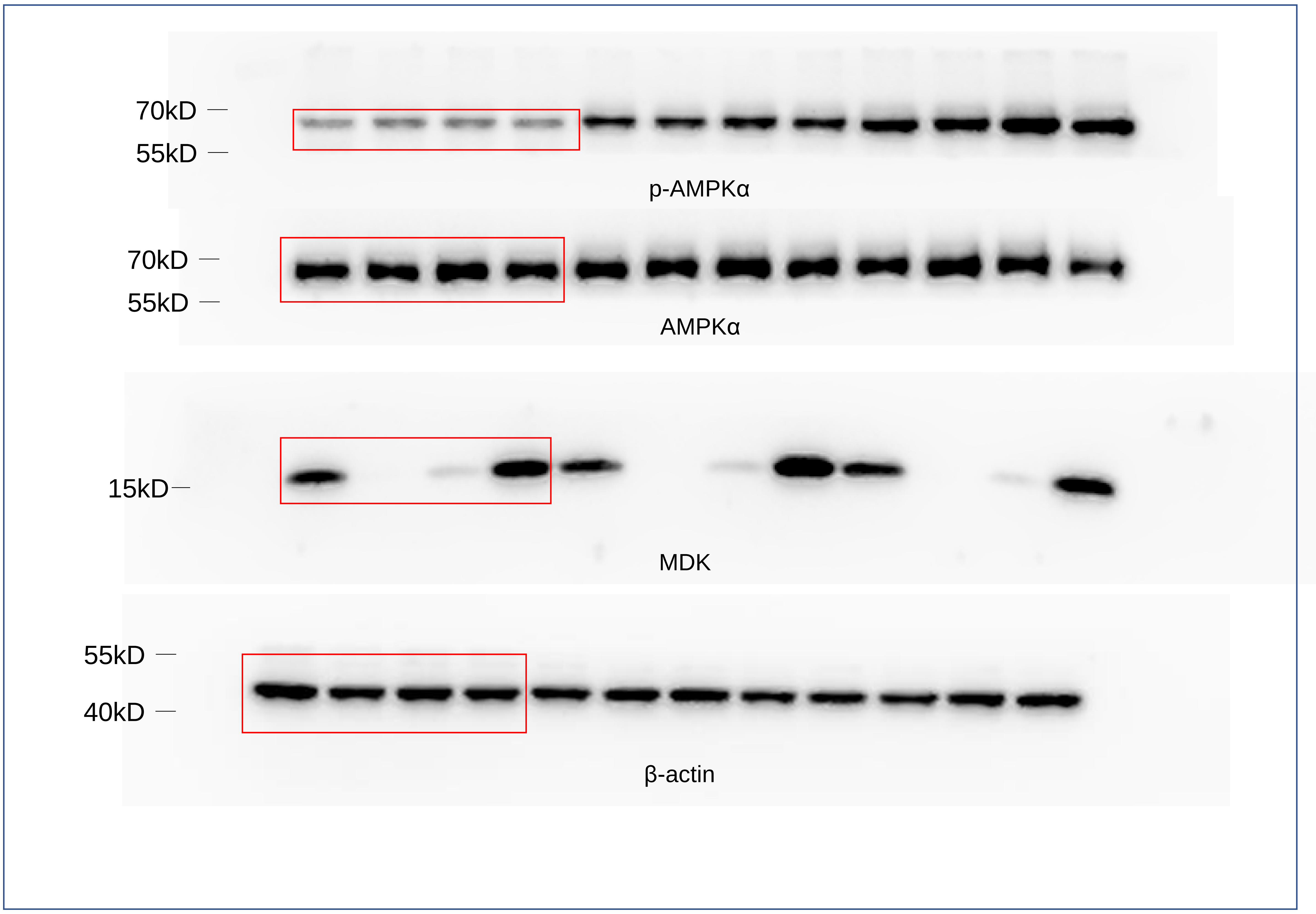

Supplemental Figure 1H

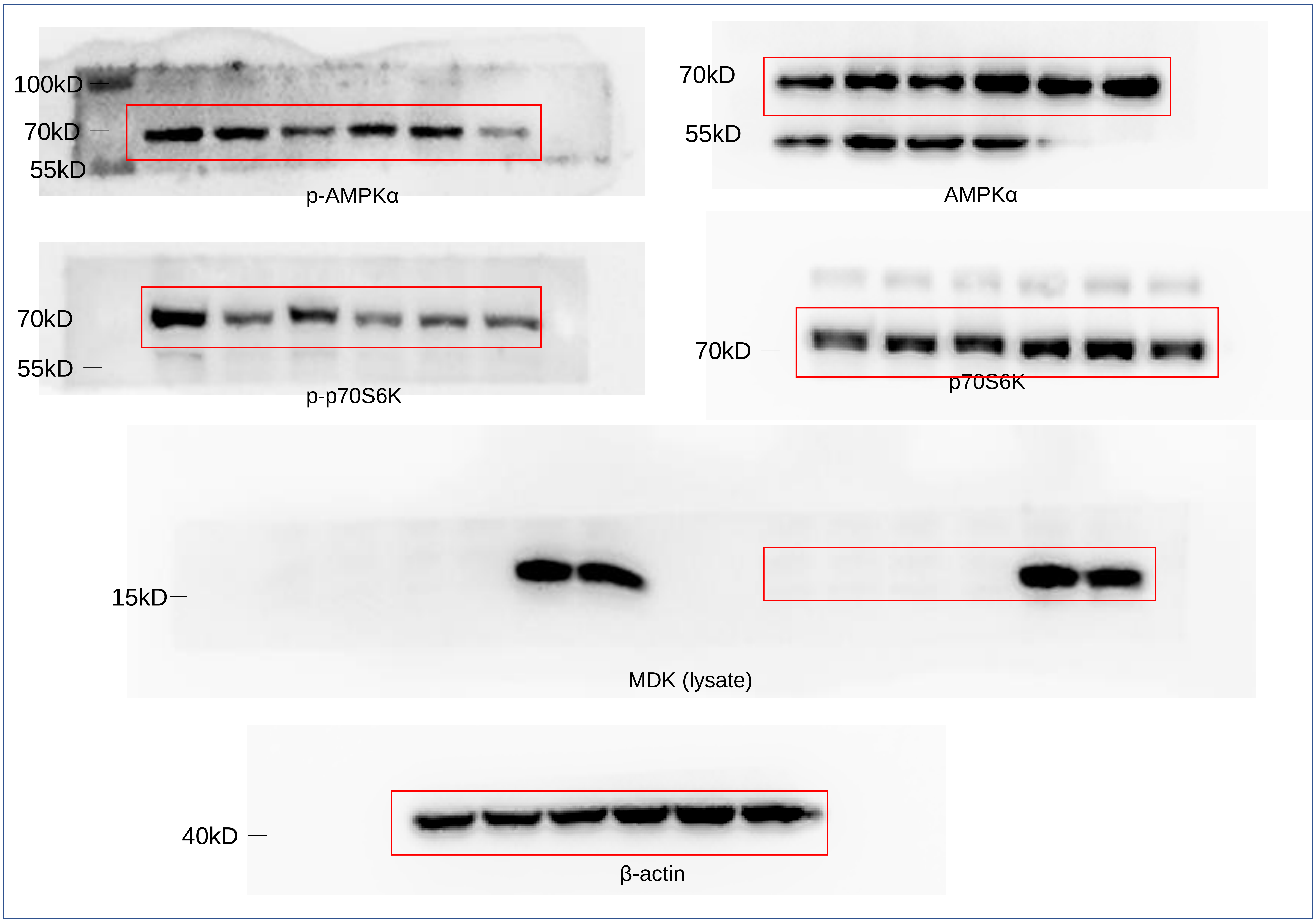


Supplemental Fig.1I

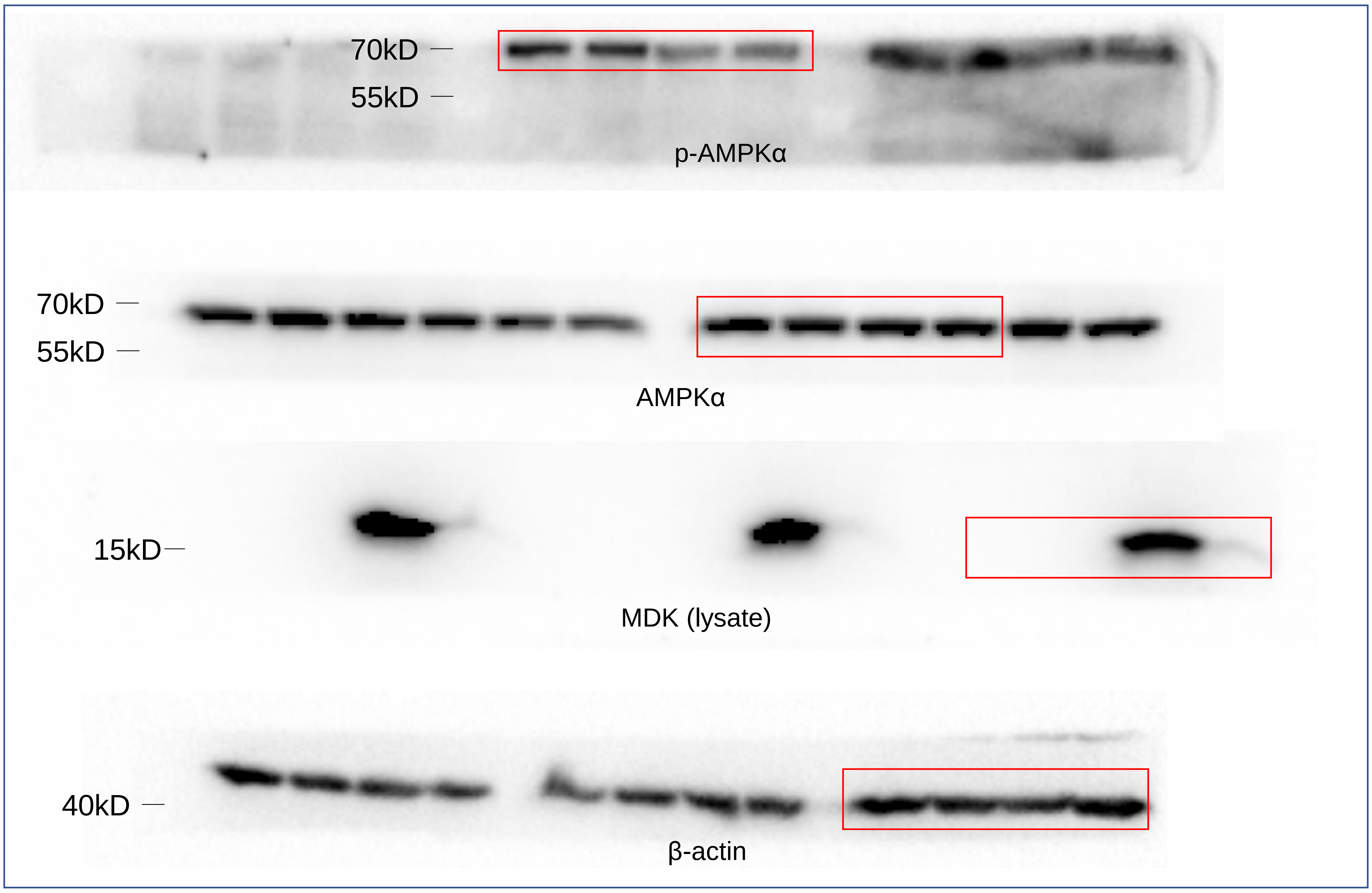

Supplemental Figure 2B

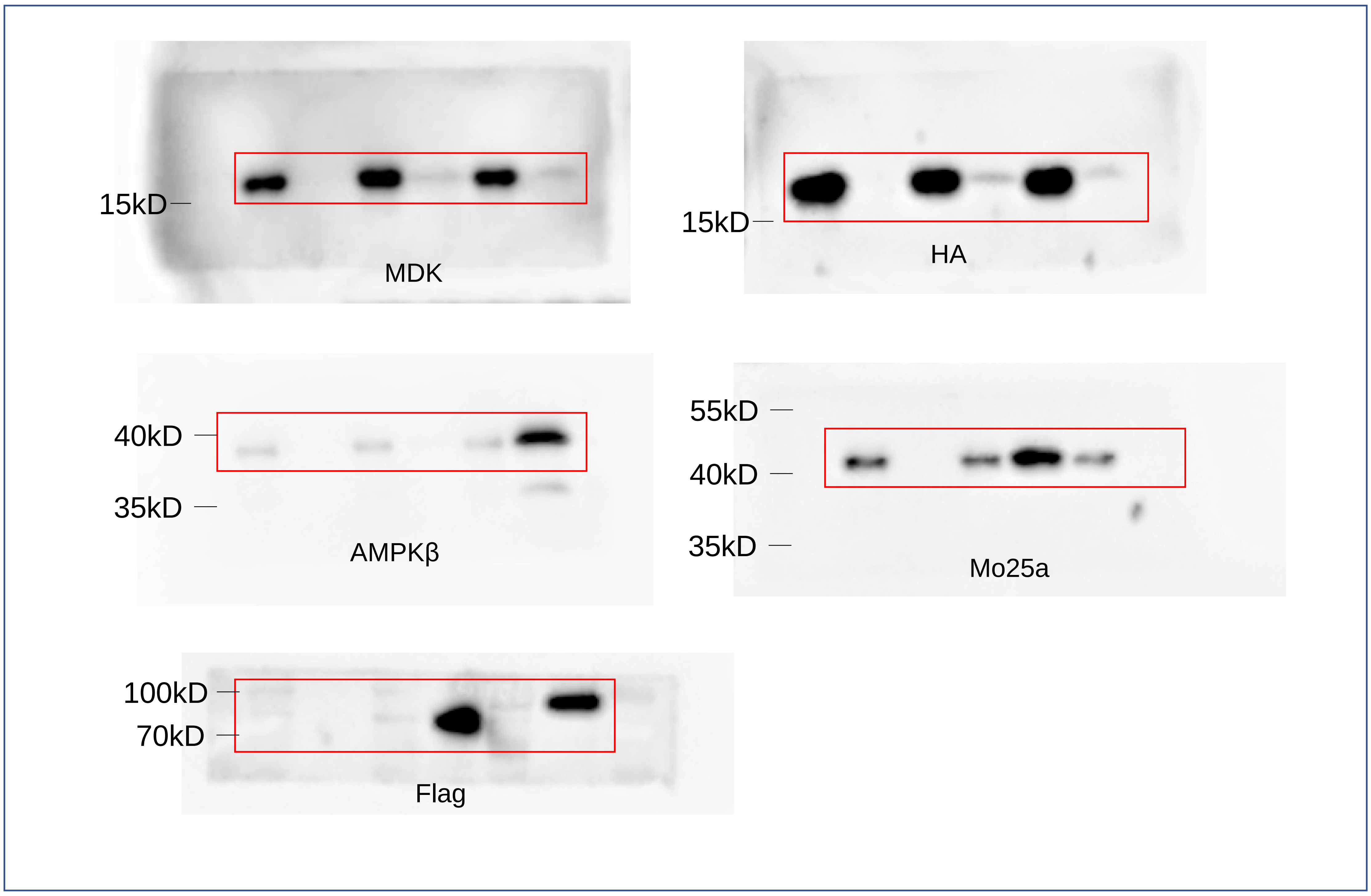


Supplemental Figure 2C

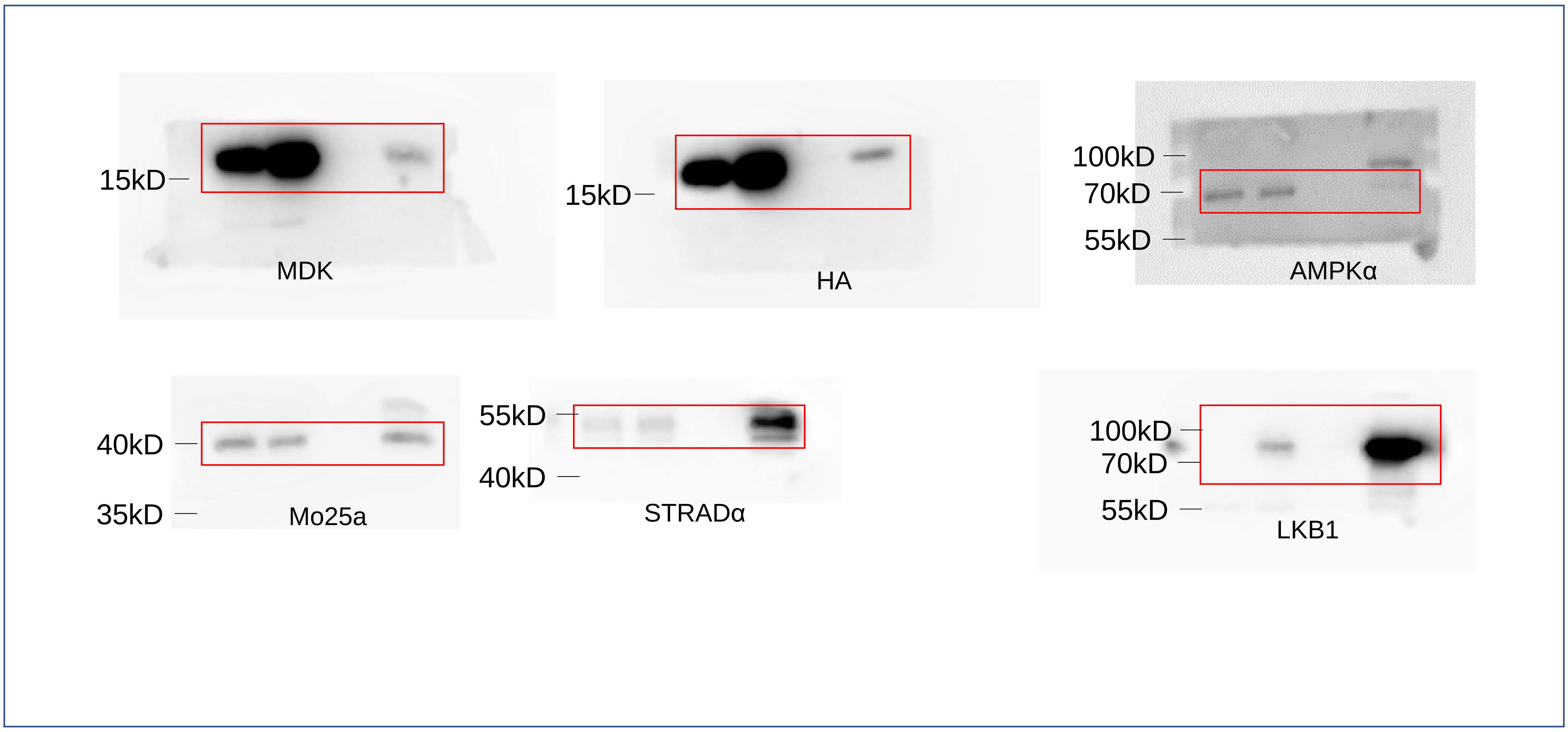

Supplemental Figure 2D

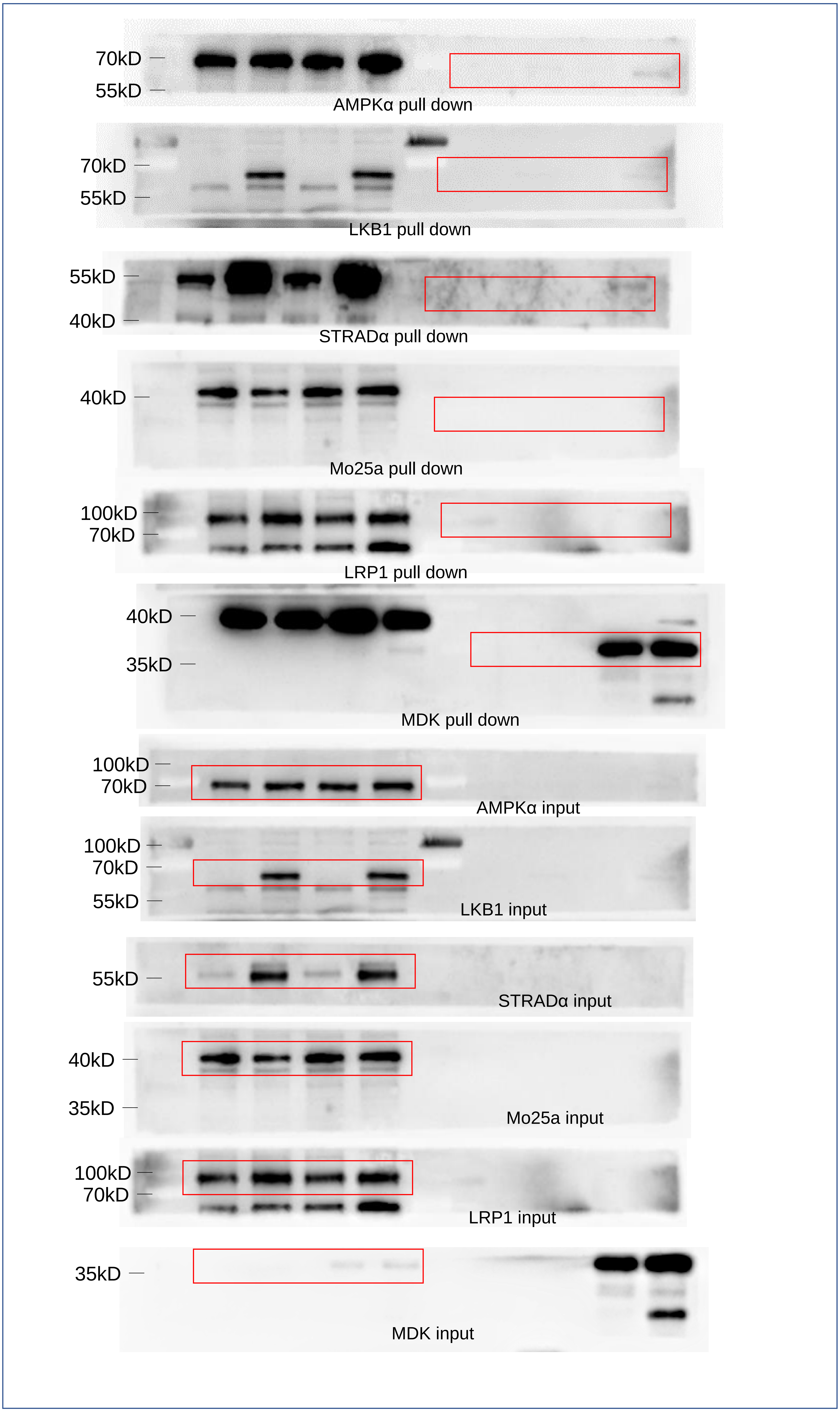

Supplemental Figure 2F

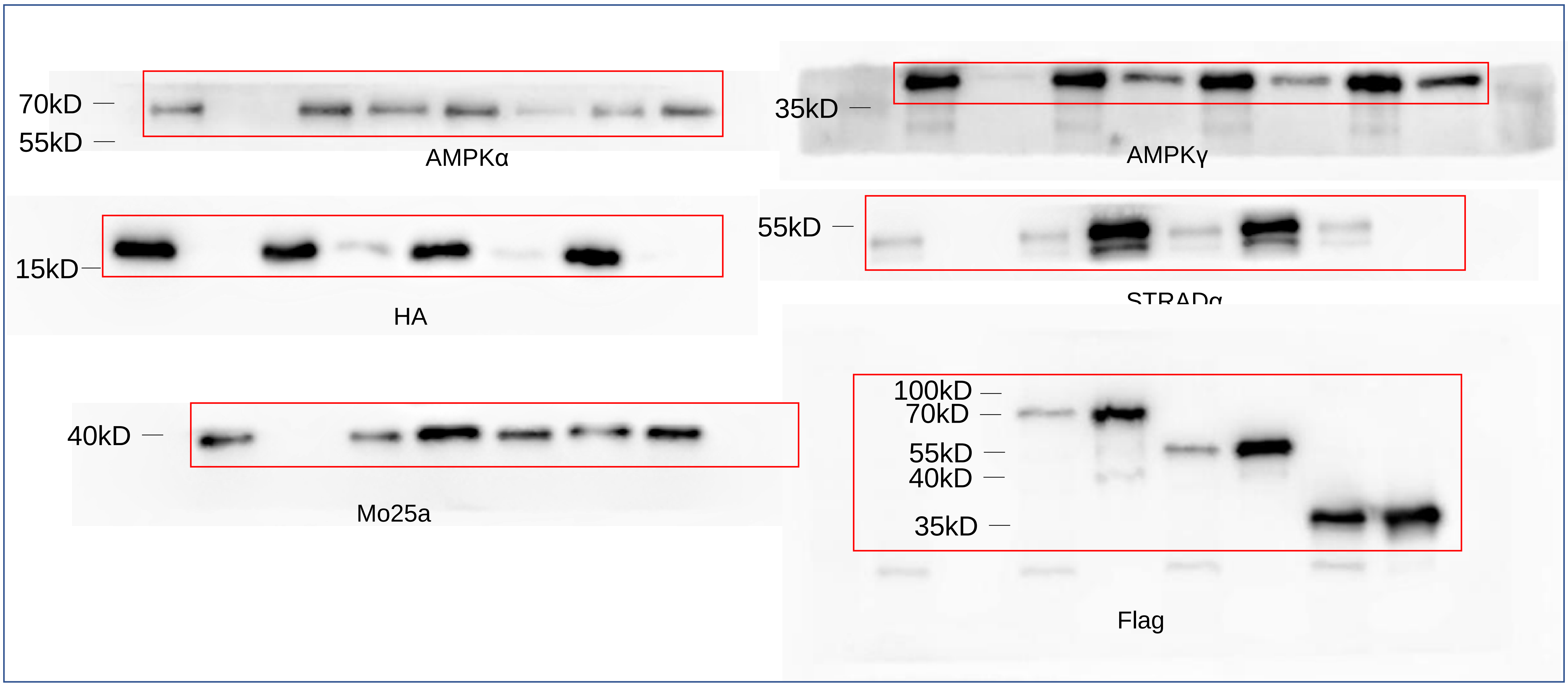


Supplemental Figure 3A

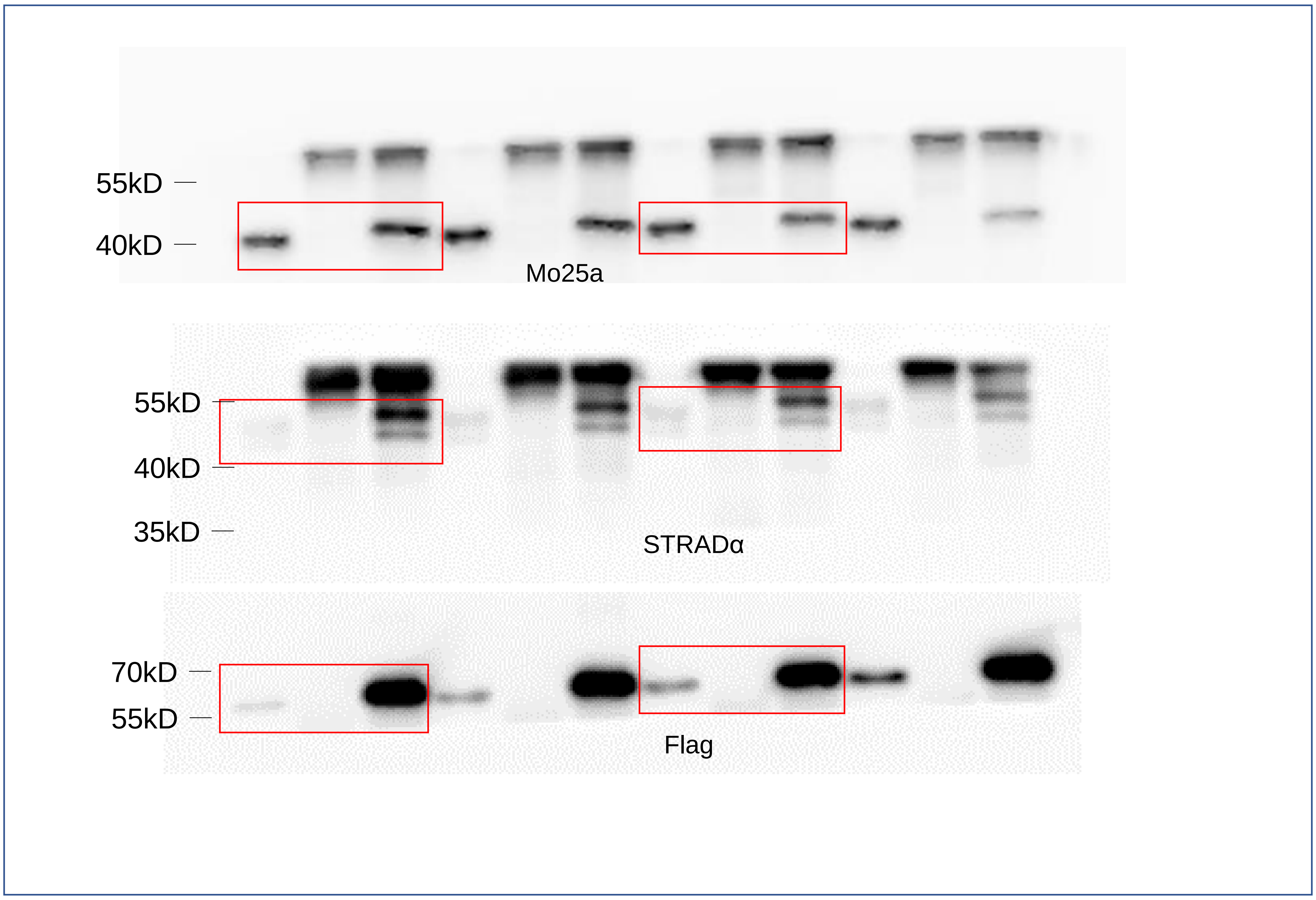

Supplemental Figure 3B

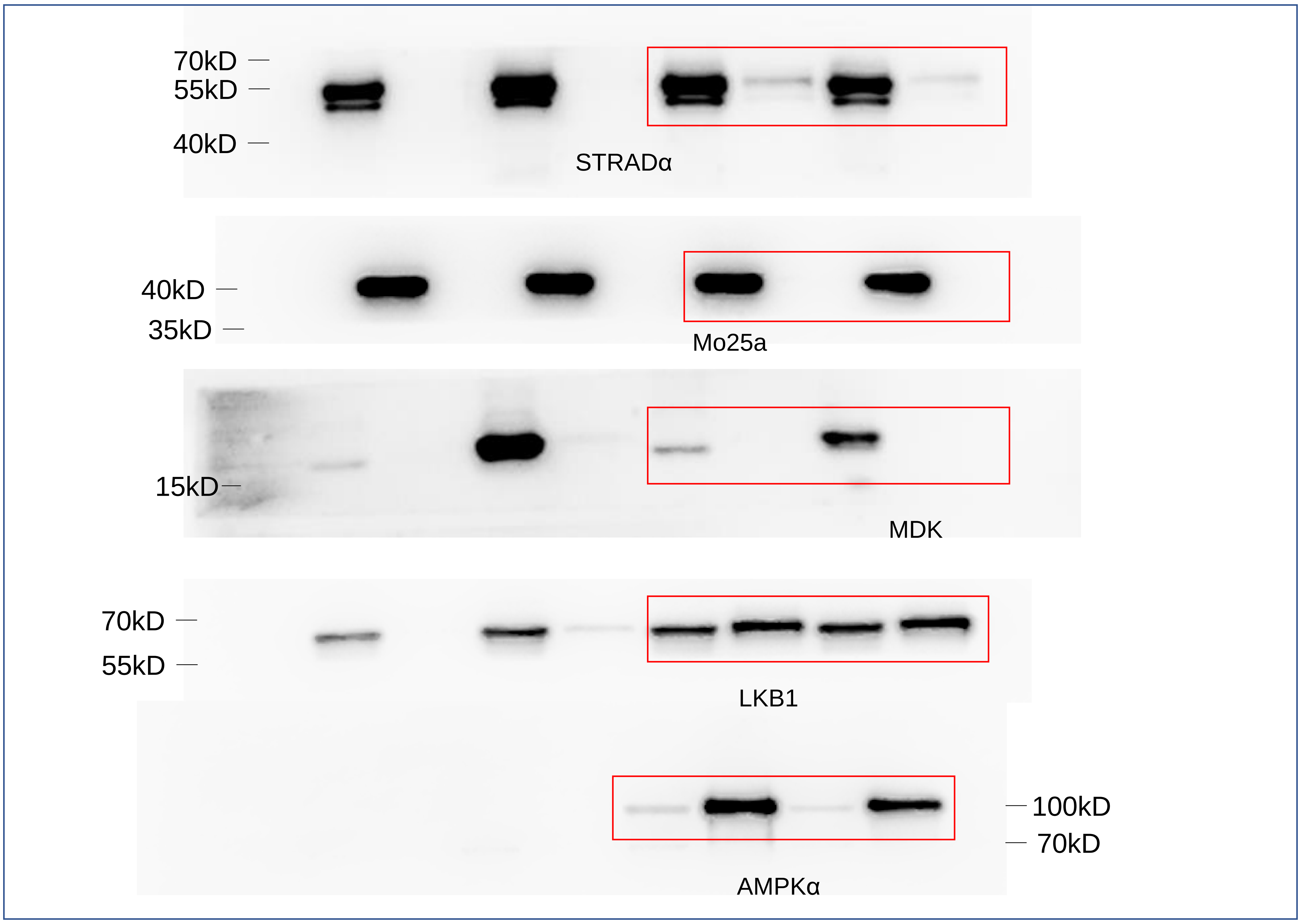

Supplemental Figure 3C

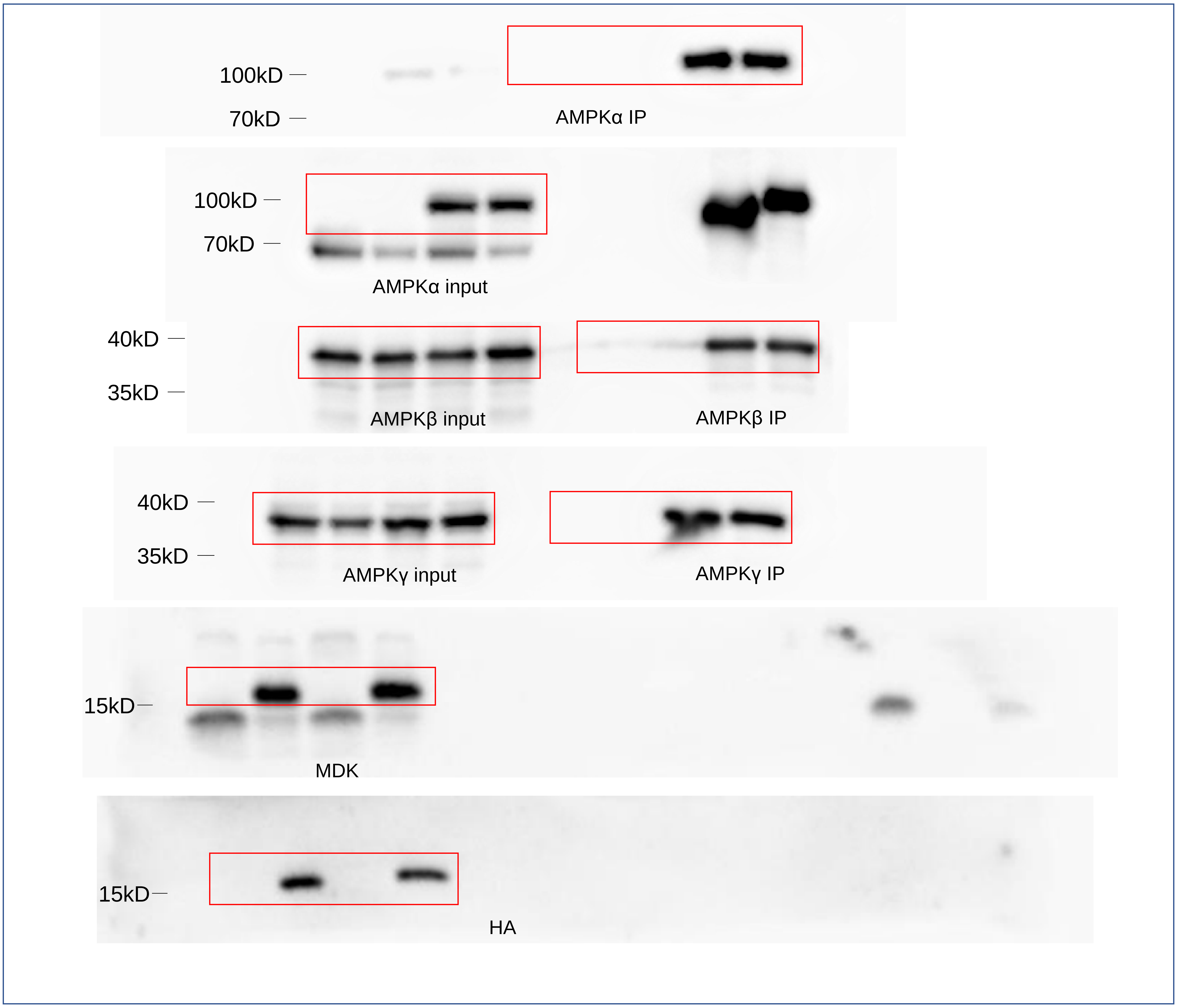

Supplemental Figure 3D

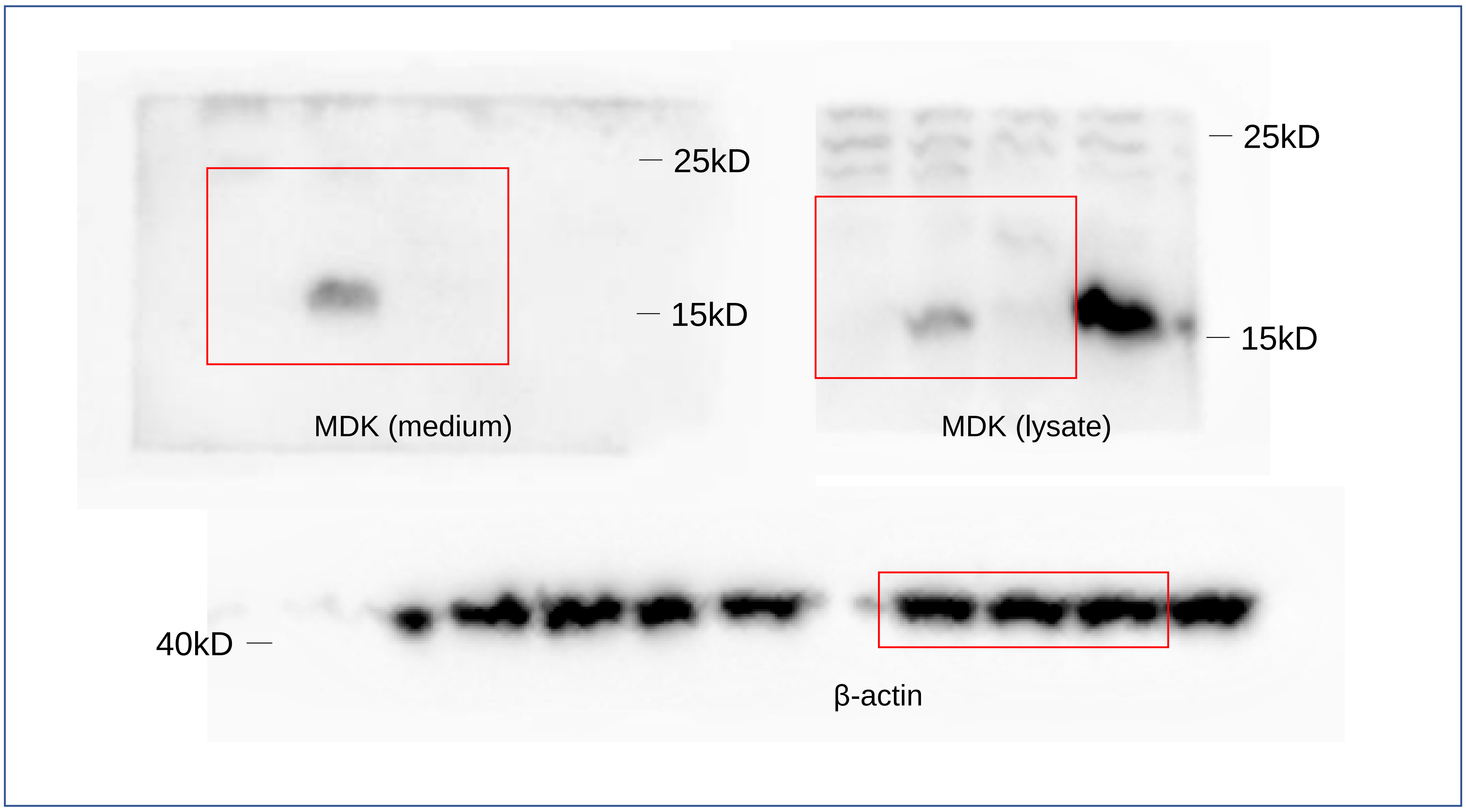


Supplemental Figure 3E

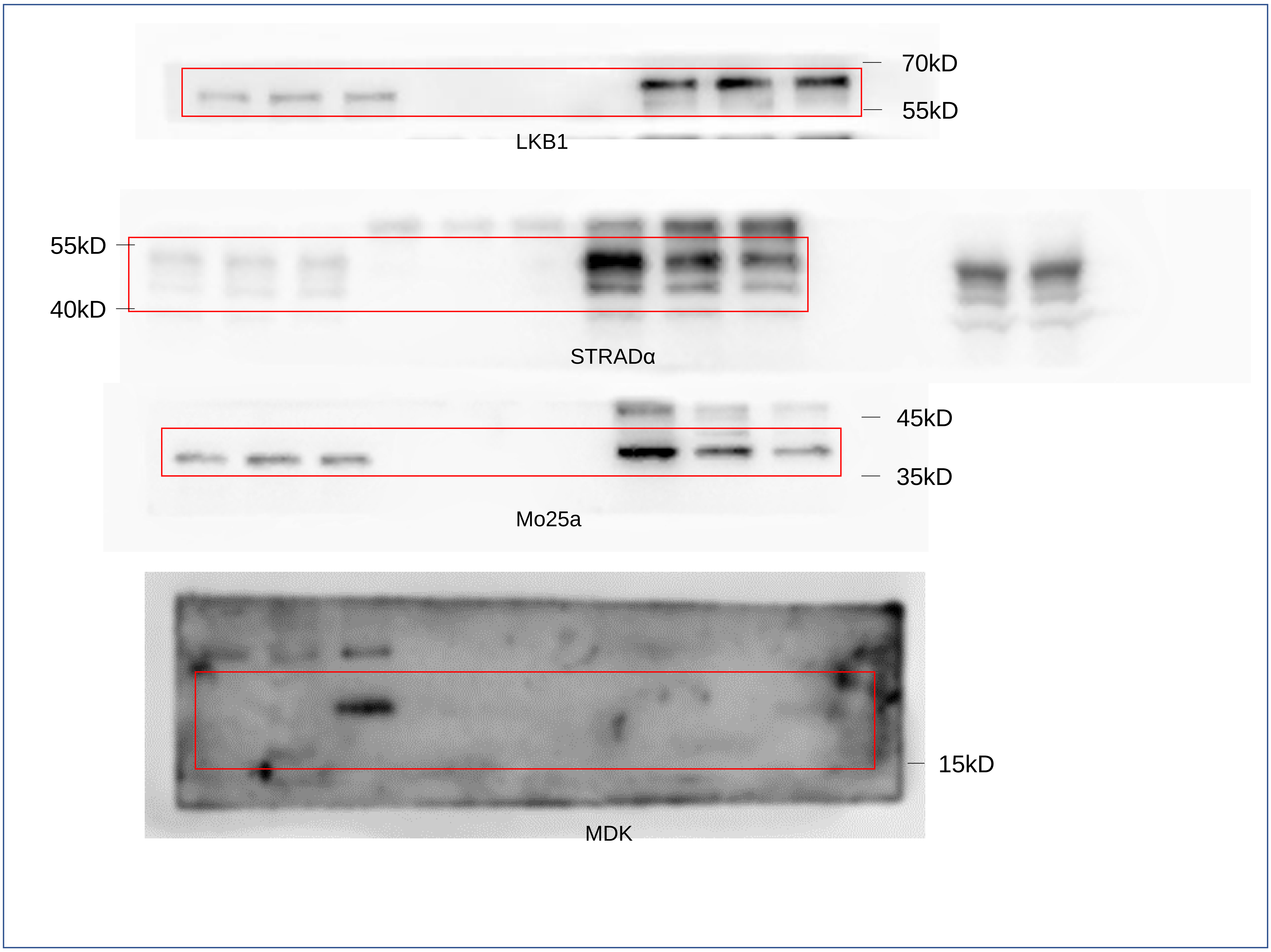

Supplemental Figure 3F

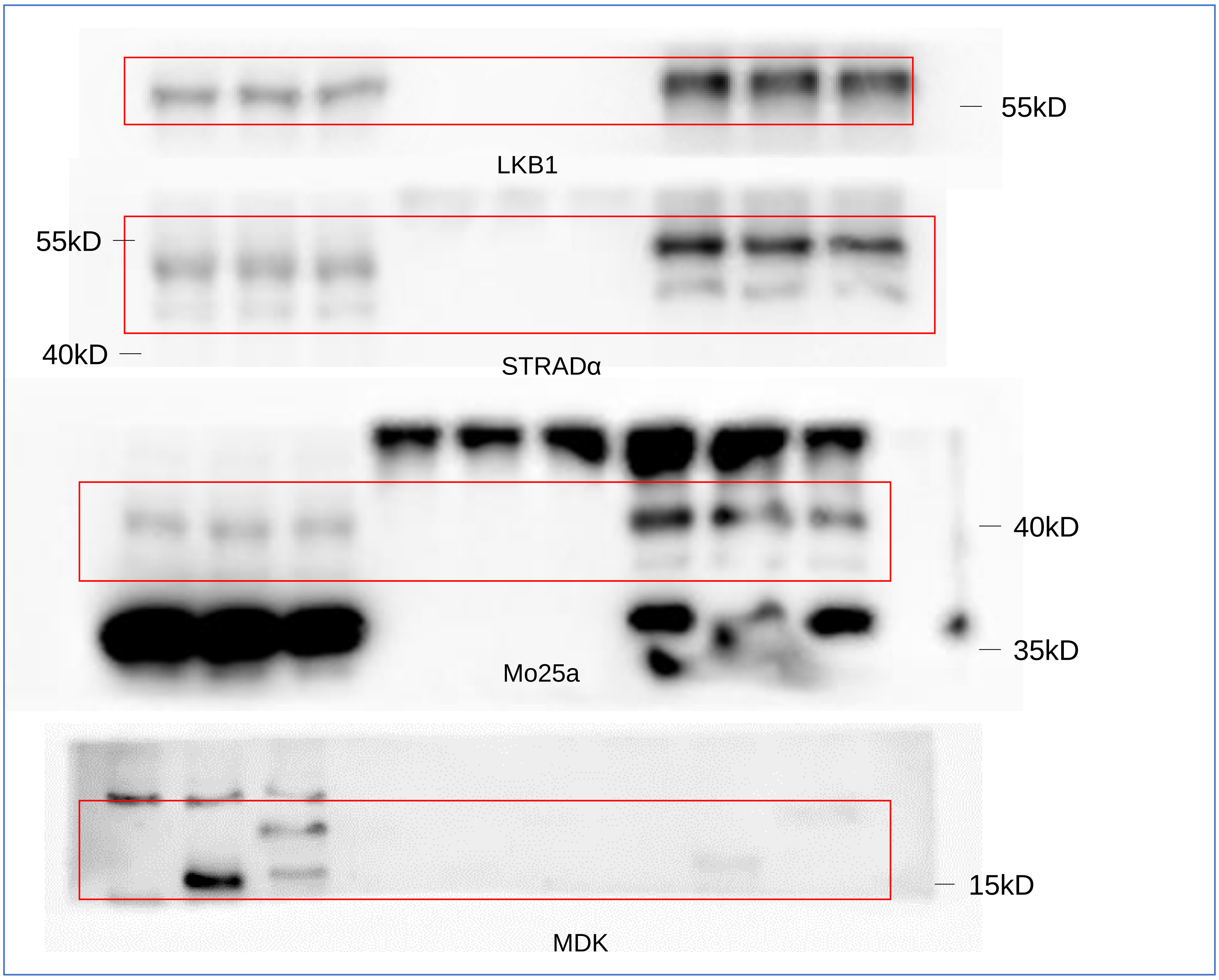


Supplemental Figure 3G

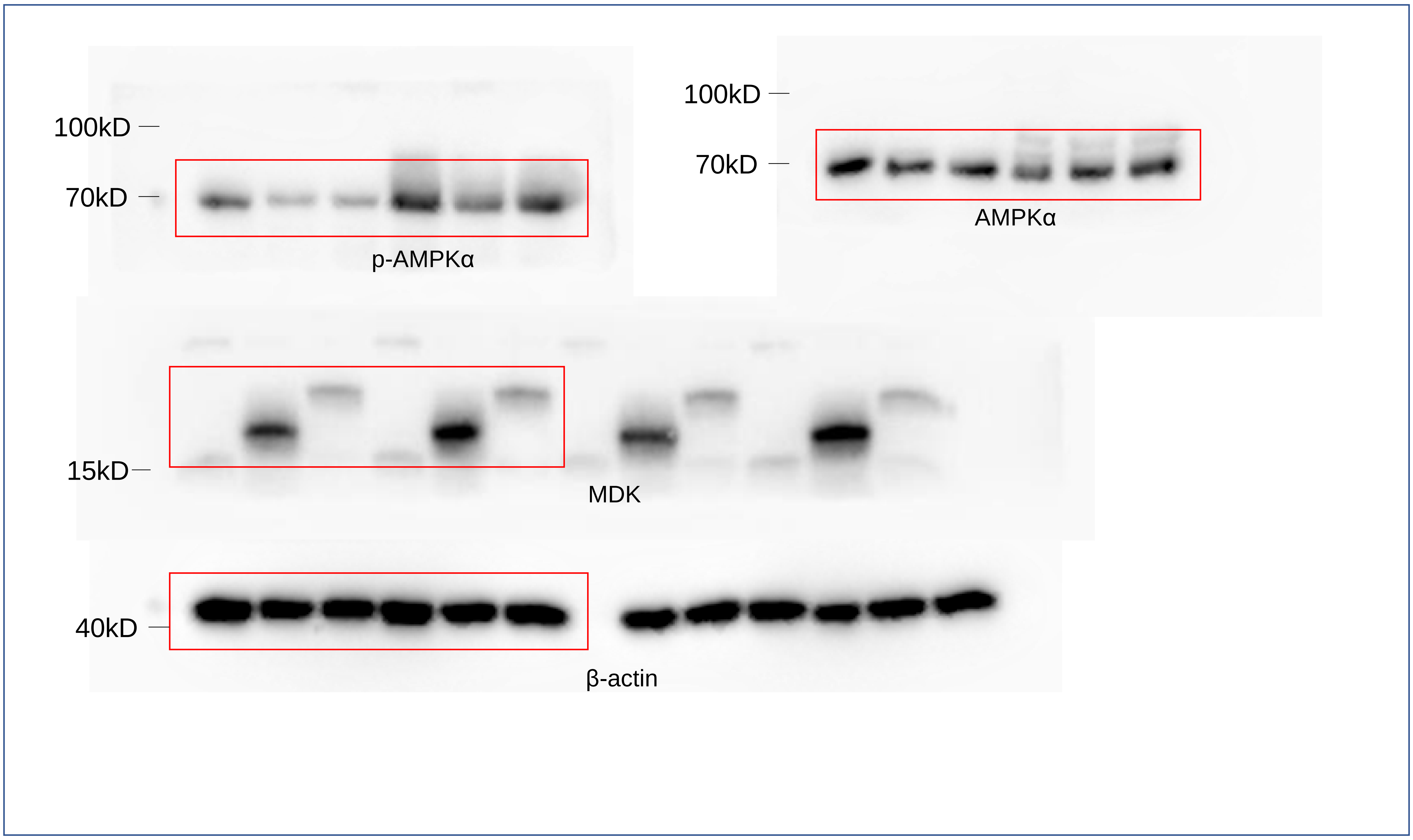

Supplemental Figure 4C

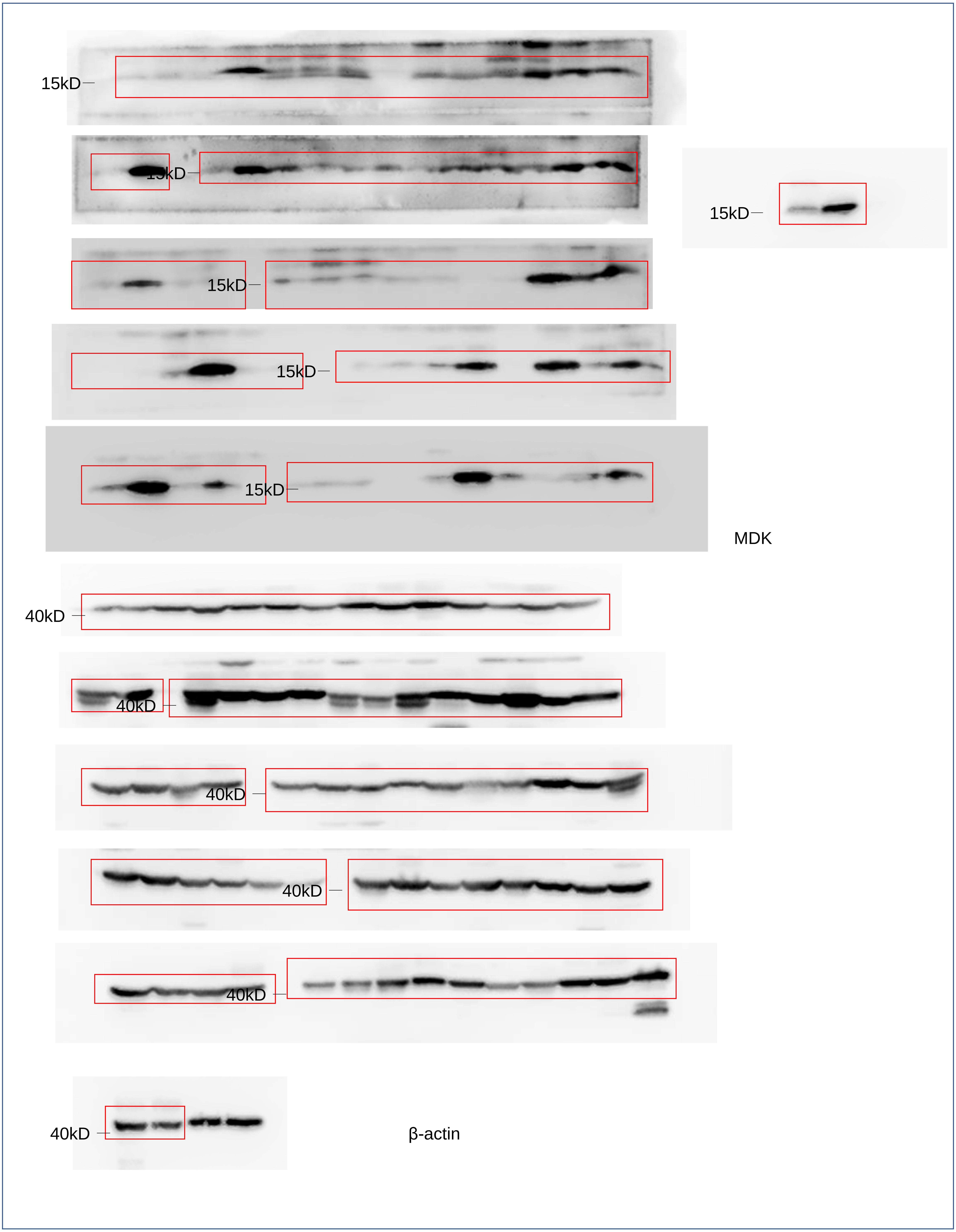

Supplemental Figure 5A

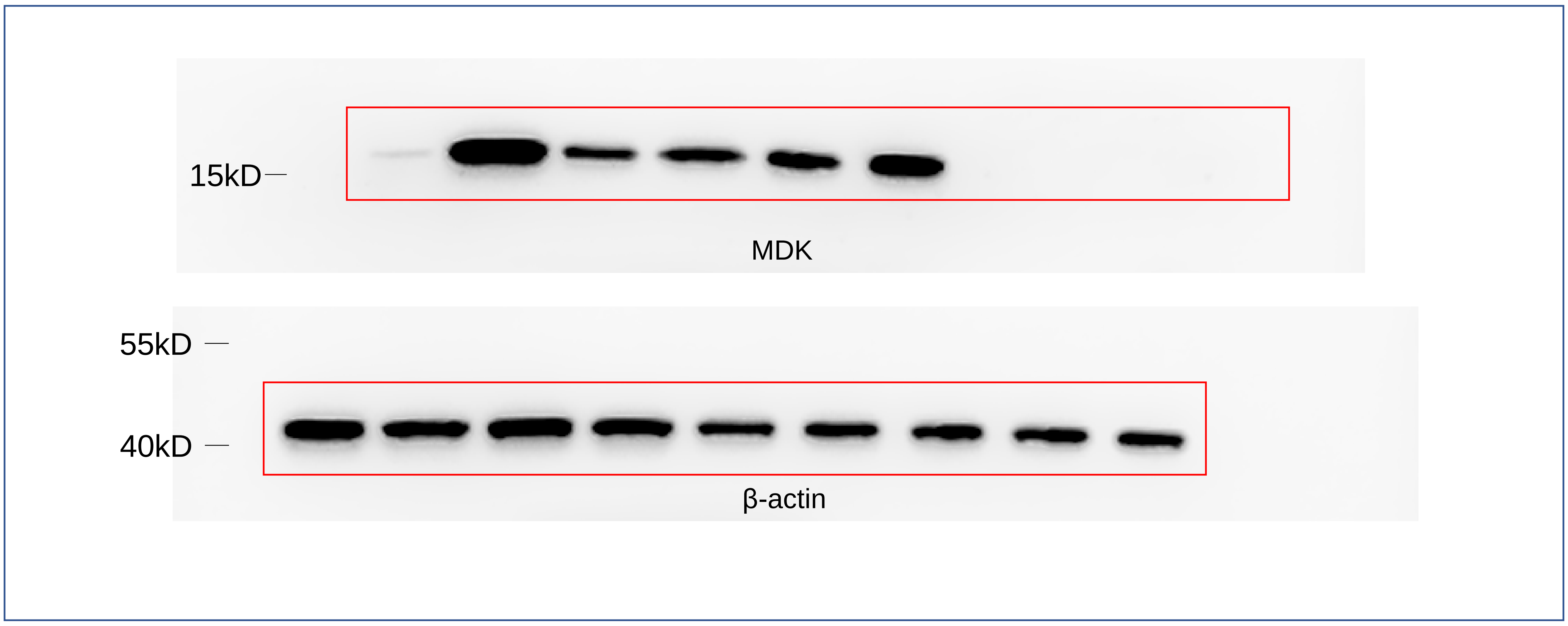


Supplemental Figure 5B

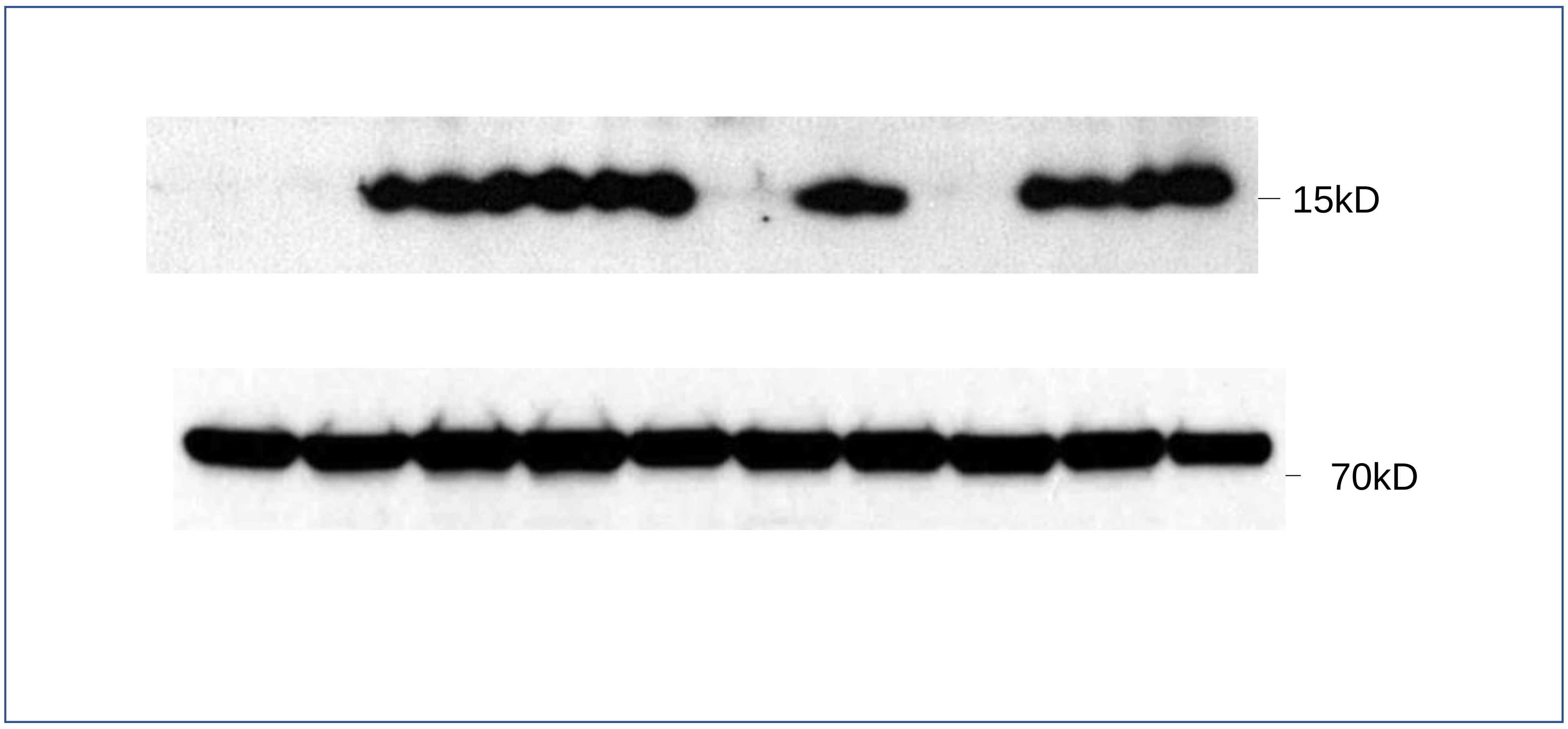


Supplemental Figure 5D

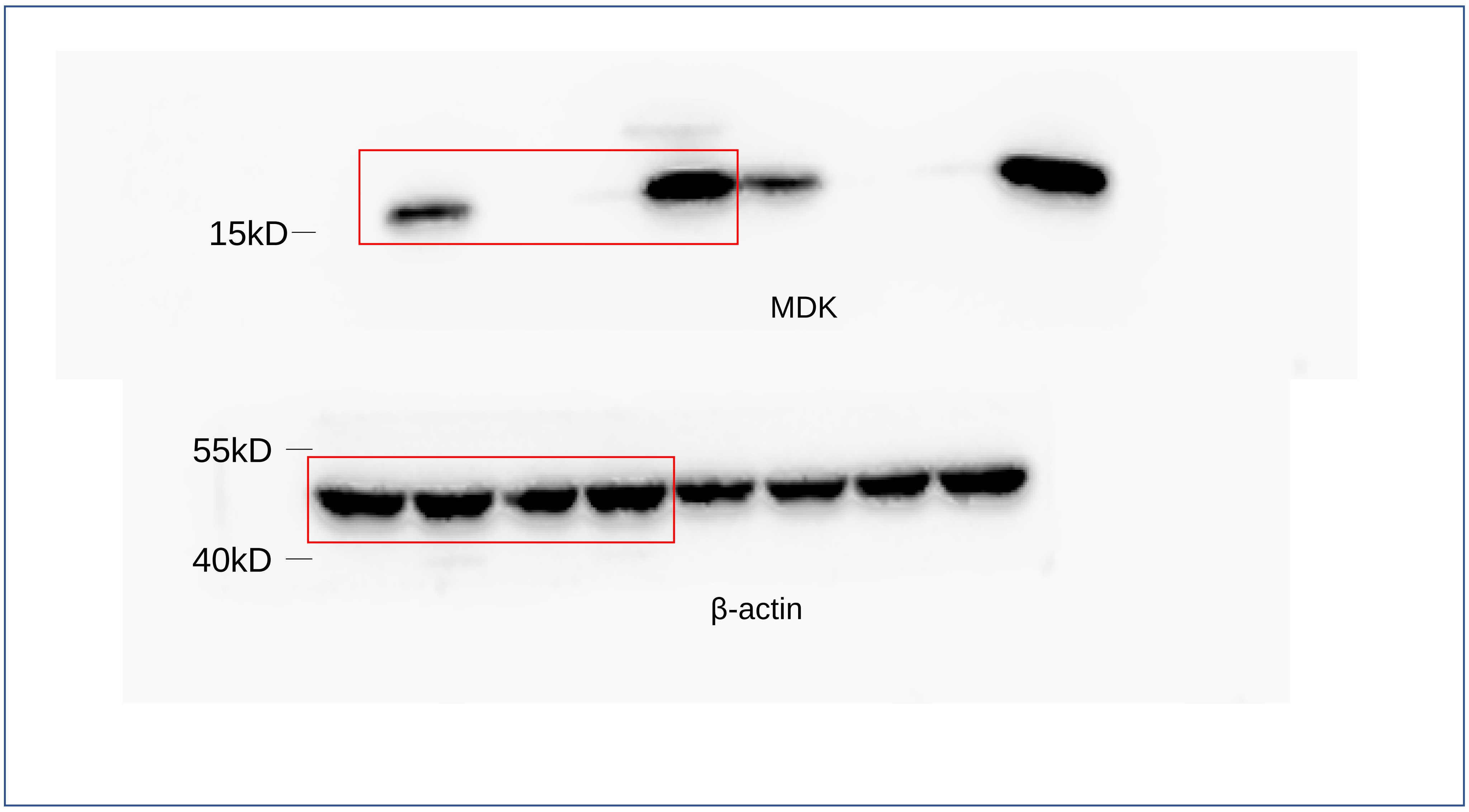

Supplemental Figure 5G

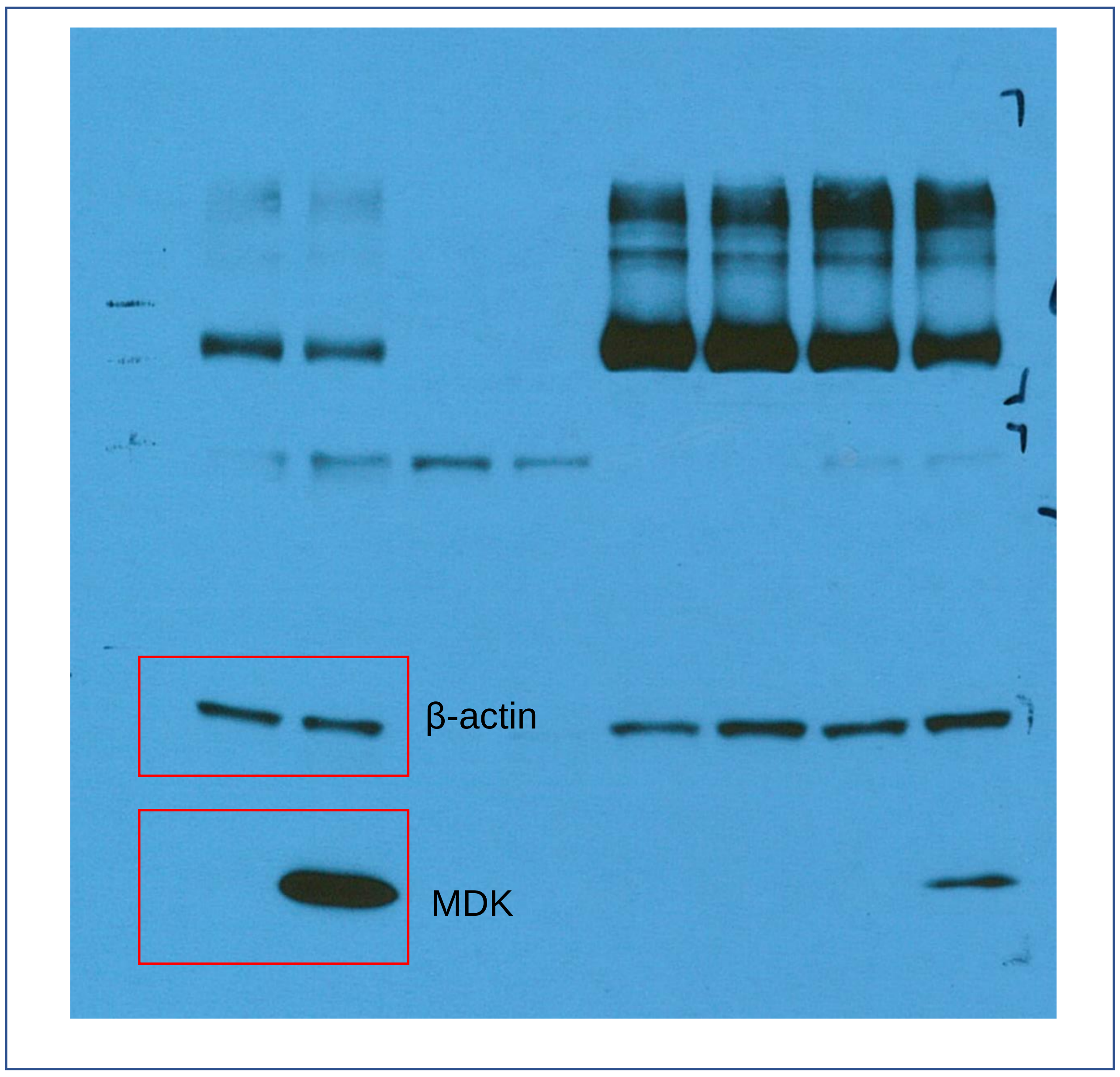


Supplemental Figure 5J

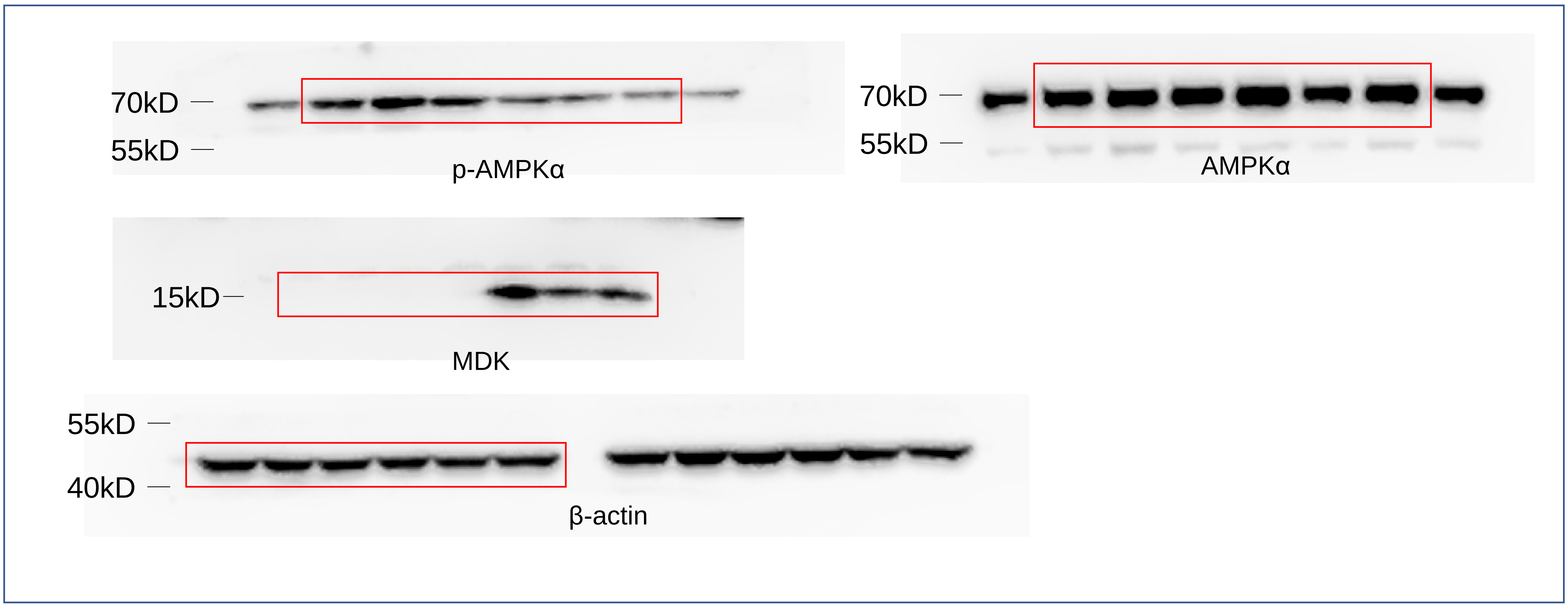

Supplemental Figure 6e

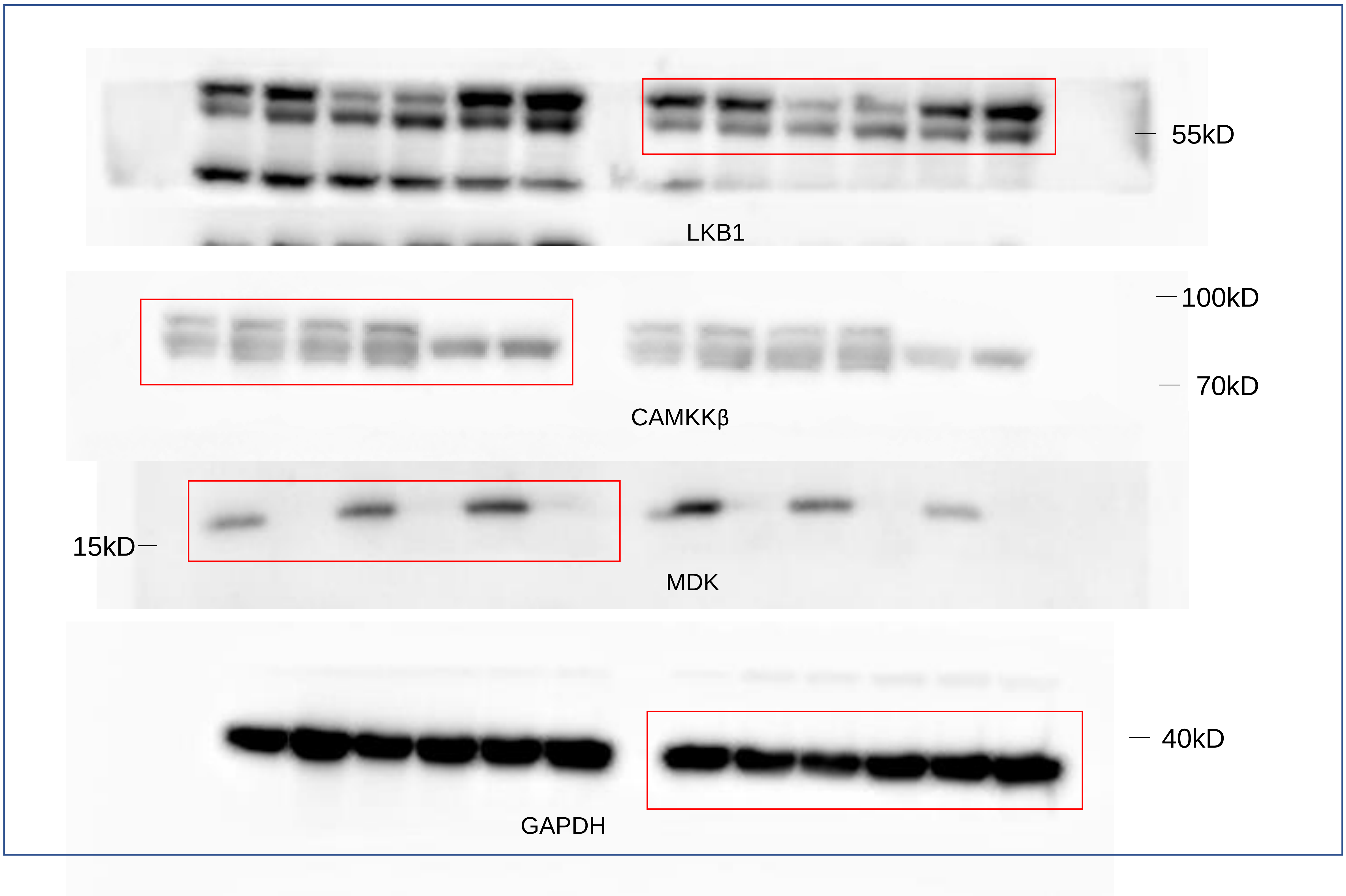


Supplemental Figure 6h

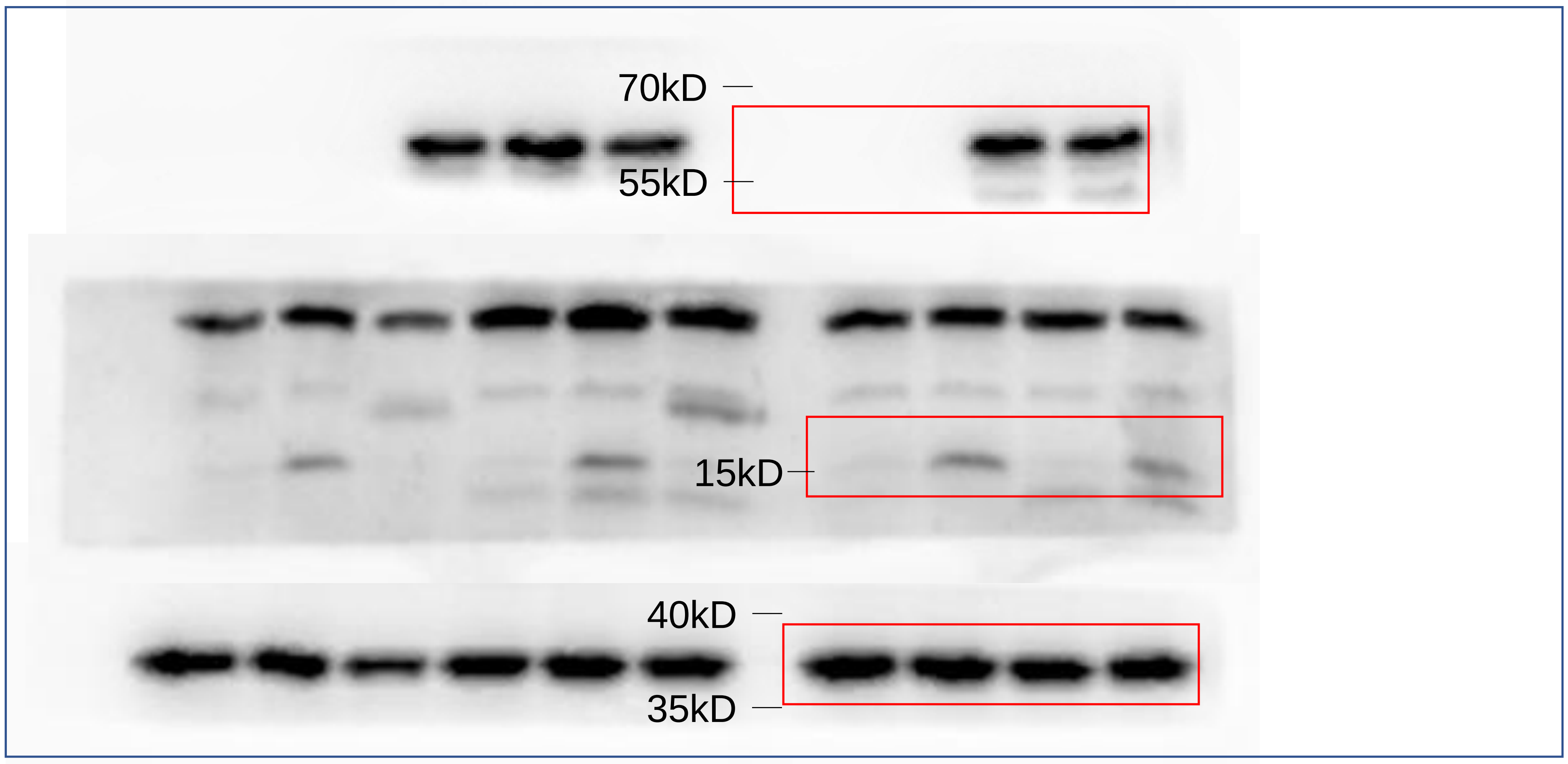

Supplement: Supplementary file 9 — Original WB images [file 41419_2022_4801_MOESM9_ESM.pdf]
